# Basic Promotors Impact Thermodynamics and Catalyst Speciation in Homogeneous Carbonyl Hydrogenation

**DOI:** 10.1021/jacs.2c00548

**Published:** 2022-04-27

**Authors:** Wenjun Yang, Tejas Y. Kalavalapalli, Annika M. Krieger, Taras A. Khvorost, Ivan Yu. Chernyshov, Manuela Weber, Evgeny A. Uslamin, Evgeny A. Pidko, Georgy A. Filonenko

**Affiliations:** † Inorganic Systems Engineering Group, Department of Chemical Engineering, Faculty of Applied Sciences, Delft University of Technology, Van der Maasweg 9, 2629 HZ Delft, The Netherlands; ‡ TheoMAT Group, ChemBio Cluster, ITMO University, Lomonosova 9, St. Petersburg 191002, Russia; § Institute of Chemistry and Biochemistry, Freie Universität Berlin, Fabeckstraße 34/36, Berlin D-14195, Germany

## Abstract

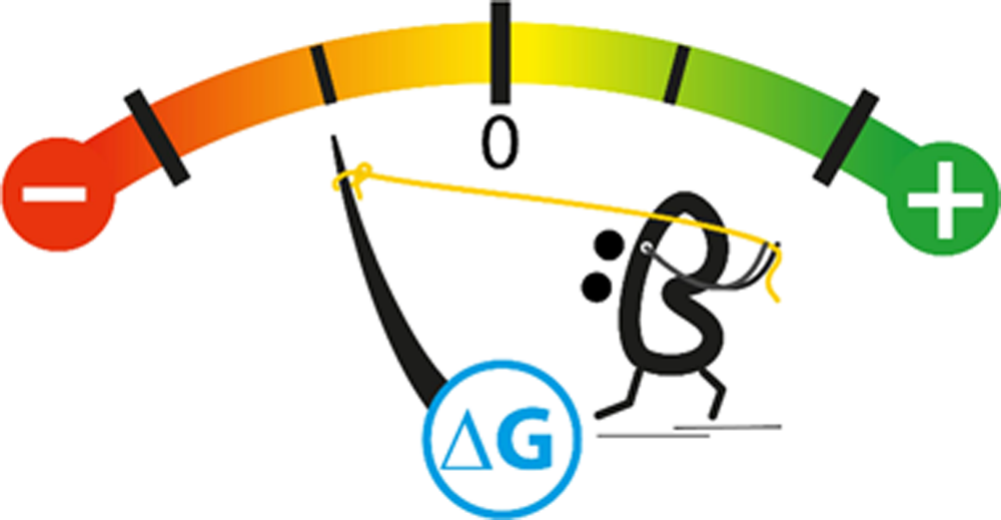

Homogeneously catalyzed reactions often make use of additives and promotors that affect reactivity patterns and improve catalytic performance. While the role of reaction promotors is often discussed in view of their chemical reactivity, we demonstrate that they can be involved in catalysis indirectly. In particular, we demonstrate that promotors can adjust the thermodynamics of key transformations in homogeneous hydrogenation catalysis and enable reactions that would be unfavorable otherwise. We identified this phenomenon in a set of well-established and new Mn pincer catalysts that suffer from persistent product inhibition in ester hydrogenation. Although alkoxide base additives do not directly participate in inhibitory transformations, they can affect the equilibrium constants of these processes. Experimentally, we confirm that by varying the base promotor concentration one can control catalyst speciation and inflict substantial changes to the standard free energies of the key steps in the catalytic cycle. Despite the fact that the latter are universally assumed to be constant, we demonstrate that reaction thermodynamics and catalyst state are subject to external control. These results suggest that reaction promotors can be viewed as an integral component of the reaction medium, on its own capable of improving the catalytic performance and reshaping the seemingly rigid thermodynamic landscape of the catalytic transformation.

## Introduction

**Figure 1:**
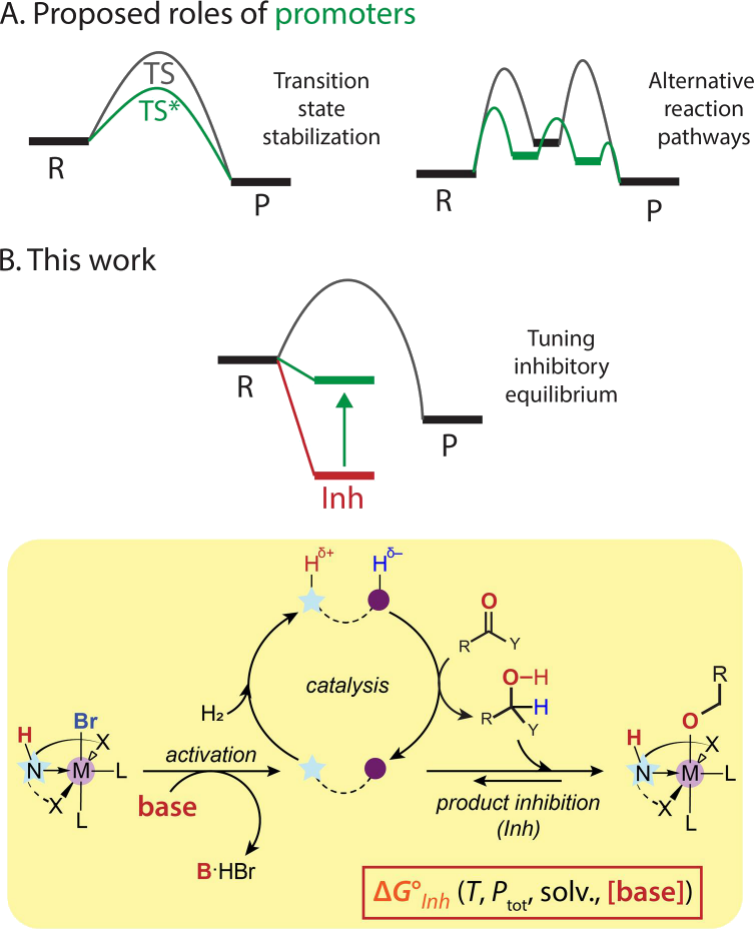
Proposed functions of promotors in catalysis (A) and the environmental role of basic promotors (B) described in this work for homogeneous ester hydrogenation.

The use of additives and promotors is a common strategy for improving rates and yields of catalytic transformations^1^ and because of the complexity of real catalysis, the roles of promotors are challenging to investigate. Promotors and additives are proposed to take on various roles in catalysis with the majority of studies suggesting their direct participation in the steps of the catalytic cycle. Namely, additives can give rise to new reaction pathways or enhance the catalytic performance by stabilizing the transition states of established pathways (Figure 1A). This *stoichiometric* view of promotors remains dominant in catalysis as it has observable experimental manifestations. Recent precedents, however, offer an alternative view on the role of reaction additives and, in contrast to the stoichiometric reactivity, catalytic promotors were found to tune the properties of the reaction medium and affect the catalytic transformations indirectly. The first mechanistically resolved example of such *environmental* promotion was recently described by Liu, Lercher and co-workers who showed that water in zeolite catalysts could increase the chemical potential of reactants and reduce the reaction barriers through variations of ionic strength. While not involving specific chemical reactivity, the use of water additive has perturbed the catalytic environment and led to a dramatic increase of reaction rates.^2^

In this work we establish the first precedent of such environmental promotion effects in homogeneous hydrogenation. Comprising a vast class of reactions, catalytic hydrogenations have high industrial relevance.^3^ Utilizing molecular hydrogen with an appropriate catalyst, these reactions can convert unsaturated functional groups in a variety of substrates to their saturated counterparts. Out of numerous functional groups that can be reduced in this way, esters pose a significant challenge for direct catalytic hydrogenation and their conversion have been explored using various transition metals,^4^ ranging from ruthenium, iridium, and osmium to iron, cobalt, and manganese.^5−7^ Most of these catalysts are known to rely on an alkoxide base promotor typically used in superstoichiometric amounts with respect to the catalyst, far beyond those necessary for precatalyst activation per se.^5a−5h,5j^ Manganese catalysts are particularly reliant on the high base loadings with 10-fold excess of alkoxide base with respect to catalyst being commonly used for ester substrates.^8^

The necessity of alkoxide base additives was suggested in two proposals: (1) the coordination of the alkali cation of the base promotor to metal amido complexes can facilitate the H_2_ activation^9^ step and (2) replacement of the N–H moiety of metal hydride species with N–K enabled by the base promotor that can enhance the reactivity of metal hydride species in the hydrogen transfer reaction.^9,7n^ Although these hypothesis provide mechanistic rationale, their direct involvement into the catalytic performance has not been quantitatively assessed, making the alkoxide base a ubiquitous promoter with poorly understood mechanism of action.

In this work we demonstrate that alkoxide bases can affect the thermodynamics of homogeneous ester hydrogenation with manganese(I) pincer catalysts and adopt a role of nonstoichiometric environmental promotor. We show that ester hydrogenation suffers from profound product inhibition caused by the reversible interaction of catalytically competent species with alcohol products (Figure 1B). Using a combination of operando spectroscopy,^10^ density functional theory (DFT) calculations and reactivity studies, we directly identify that the primary function of the base promotor is to suppress this inhibition process and prolong the lifetime of reactive catalyst species. Strikingly, this implies that the alkoxide promotor is not chemically involved in the inhibitory equilibrium, but can tune its standard thermodynamic parameters and make it unfavorable via the perturbation exerted on solvation medium. This introduces an entirely new parameter to be considered when examining the thermodynamics of catalytic reactions, apart from the typical ones, e.g., reactants, products, temperature and solvent (Figure 1). Having confirmed its generality for Mn-promoted hydrogenations, we highlight the use of environmental promotors in homogeneous hydrogenations as an entirely new strategy to rationalize and improve the catalytic performance.

## Results and Discussion

### Synthesis and Catalytic Activity of Mn-bis-N-Heterocyclic Carbene Amino Pincers

**Figure 2:**
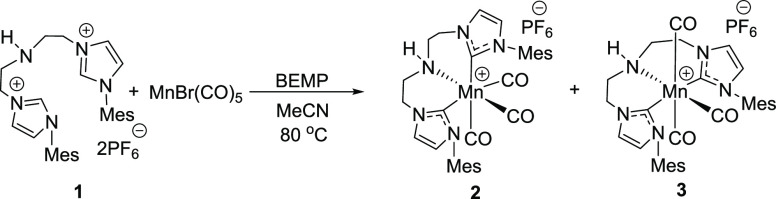
Synthesis of Mn(I) complexes **2** and **3**.

To study the role of the base promotor, we developed new Mn pincer catalysts for ester hydrogenation that were both catalytically competent and easy to track using spectroscopic methods. We based our model catalyst design on bis-N-heterocyclic carbene amino (CNC) pincer ligands that proved to be a versatile ligand motif for transition-metal hydrogenation catalysts.^5f,11^ The representative ligand **1** readily underwent complexation with Mn(CO)_5_Br in the presence of the phosphazene base BEMP (2-*tert*-butylimino-2-diethylamino-1,3-dimethylperhydro-1,3,2-diazaphosphorine) in acetonitrile at 80 °C yielding Mn complexes **2** and **3** (Figure 2) that can be isolated individually.

**Figure 3:**
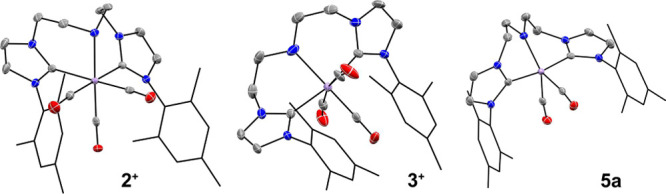
Molecular structure of complexes **2**, **3**, and **5a** in the crystal with thermal ellipsoids drawn at 50% probability. Hydrogen atoms and PF_6_ anions in cationic **2** and **3** are omitted for clarity.

As evidenced by X-ray diffraction data, the CNC ligand in these complexes adopts *facial* and *meridional* configurations for **2** and **3,** respectively (Figure 3). Exposure to ambient light slowly converted complex **2** to **3** in solution implying that **2** might be a kinetic product of the complexation.^8a^ Both complexes are cationic tricarbonyl species that are readily distinguished by ^1^H nuclear magnetic resonance (NMR) and infrared (IR) spectroscopy (see Section S2 of Supporting Information). The reaction of these complexes with KO^t^Bu converts both **2** and **3** to the dicarbonyl Mn amido species **5a** (Figure 3) with its base adduct **5b** (see Sections S9.1–S9.4 for the DFT-supported (PBE0-D3(SMD_THF_)/6–311++G(d,p)) detected by IR spectroscopy in small amounts (Figures S21 and S22). Upon exposure to H_2_, a mixture of **5a** and **5b** converts to pure **5a** with no detectible amounts of Mn hydride species, allowing to suggest that both **2** and **3** will exhibit similar catalytic activity (Figure S23).

Both Mn-CNC complexes **2** and **3** are active in ester hydrogenation. As implied by their reactivity, in the hydrogenation of the ethyl hexanoate benchmark substrate both **2** and **3** gave nearly identical conversion confirming the catalytic equivalency of these precatalysts (see Table S1) and prompting us to use complex **2** in all further studies. The most peculiar feature of the catalytic system with **2** is its reliance on the base promotor for remaining active. While only trace amounts of the alcohol product were obtained with 1 mol % KO^*t*^Bu, increasing the base amount to 10 and 20 mol % significantly increased the hexanol yield to 41 and 46%, respectively (Table 1, entries 1–3). An increase in the catalyst loading and temperature (Table 1, entries 4–6) proved beneficial for catalytic performance with 96% yield reached at 100 °C with 0.2 mol % complex **2**. Further increase of the reaction temperature to 110 °C however furnished hexanol in slightly lower yield (84%) suggesting possible catalyst degradation (entry 7). With 10 mol % KO^*t*^Bu, nearly quantitative hexanol yield could be reached in a prolonged run at 100 °C (entries 8–9).

**Table 1:**
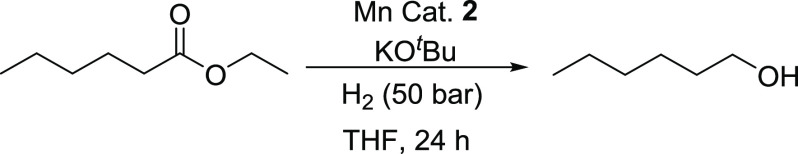
Hydrogenation of Ethyl Hexanoate with **2** under Varied Reaction Conditions^a^

entry	*T* (°C)	**2** (mol%)	KO^*t*^Bu (mol%)	conv. (%)	yield (%)
1	80	0.1	1	<1	<1
2	80	0.1	10	63	41
3	80	0.1	20	67	46
4	80	0.2	20	77	65
5	90	0.2	20	92	87
6	100	0.2	20	96	96
7	110	0.2	20	93	84
8	100	0.2	10	93	75
9^b^	100	0.2	10	99	98

a Conditions: ethyl hexanoate (1.25 mmol), Mn catalyst **2**, KO^*t*^Bu, THF (0.5 mL), *P* = 50 bar H_2_, *t* = 24 h. Conversion and yield determined by GC analysis with dodecane as the internal standard.

b Reaction was run for 48 h

While catalyst **2** was highly efficient in converting model substrates, the dependence of its activity on the base concentration prompted a further investigation into the role of the base promoter in catalysis. Given the previously proposed interactions between alkoxide base and catalytic species,^10^ we initially assumed the role of the base (Table 1, entries1–3) to be purely kinetic with the base concentration affecting the initial hydrogenation rate. Our kinetic data, however, refutes that assumption. The results presented in Figure 5A,B reveal nearly identical initial rates for hexyl hexanoate hydrogenation in the presence of **2** and 10 or 2 mol % KO^t^Bu base, ruling out any kinetically productive interactions between the catalyst and the base promotor. On the other hand, decay of the hydrogenation rate was significantly less rapid in the increased base loading experiment, suggesting that the base can be relevant to the catalyst deactivation.

### Product Inhibition and Effects of the Base Promotor on Hydrogenation Catalysis

**Figure 4:**
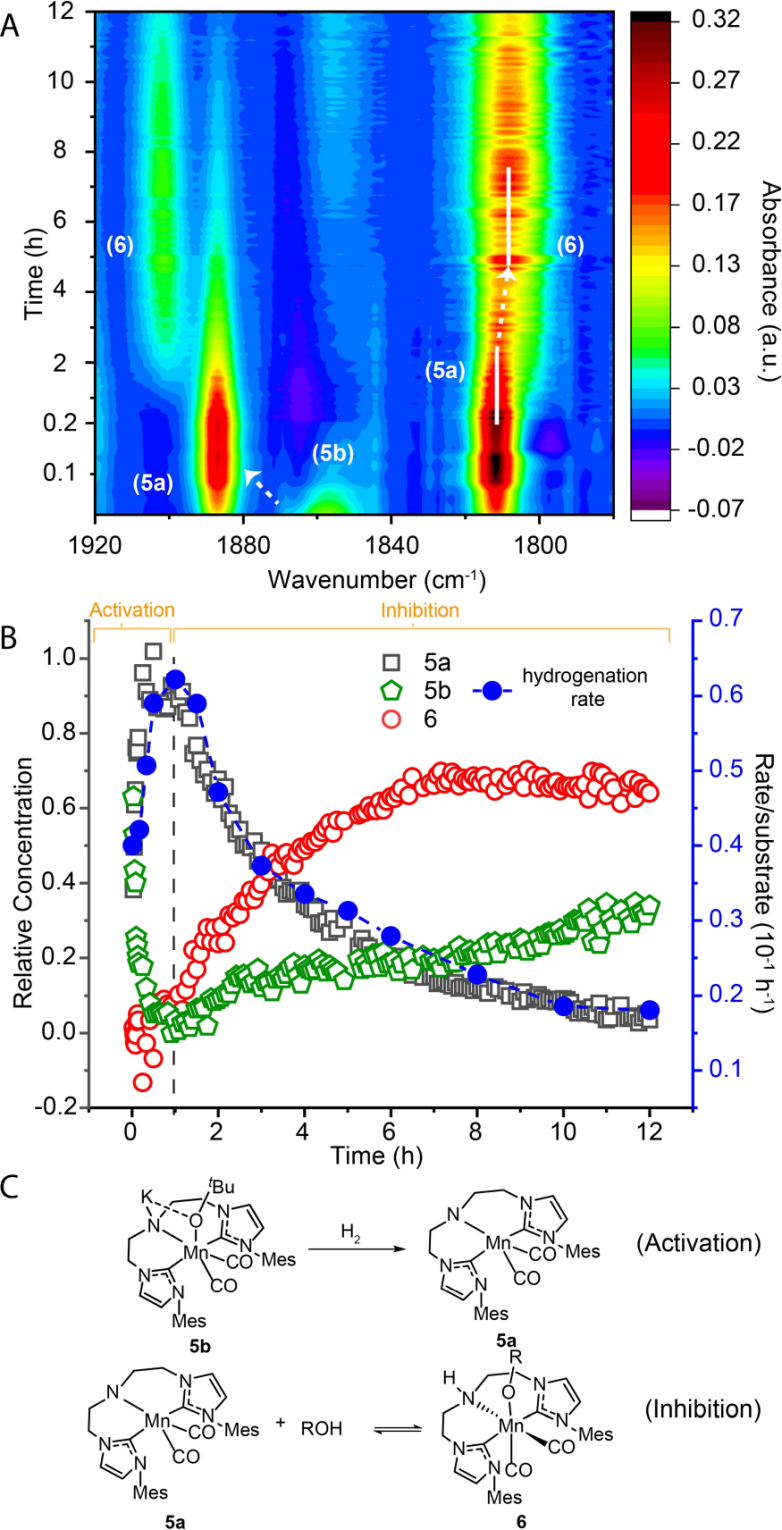
Operando IR data for the hydrogenation of ethyl hexanoate with Mn precatalyst **2** showing the evolution of carbonyl containing species (A) and relation between hydrogenation kinetics and catalyst speciation (B). Observed catalytic intermediates shown in panel C. See Section S7 in Supporting Information for reaction conditions.

**Figure 5:**
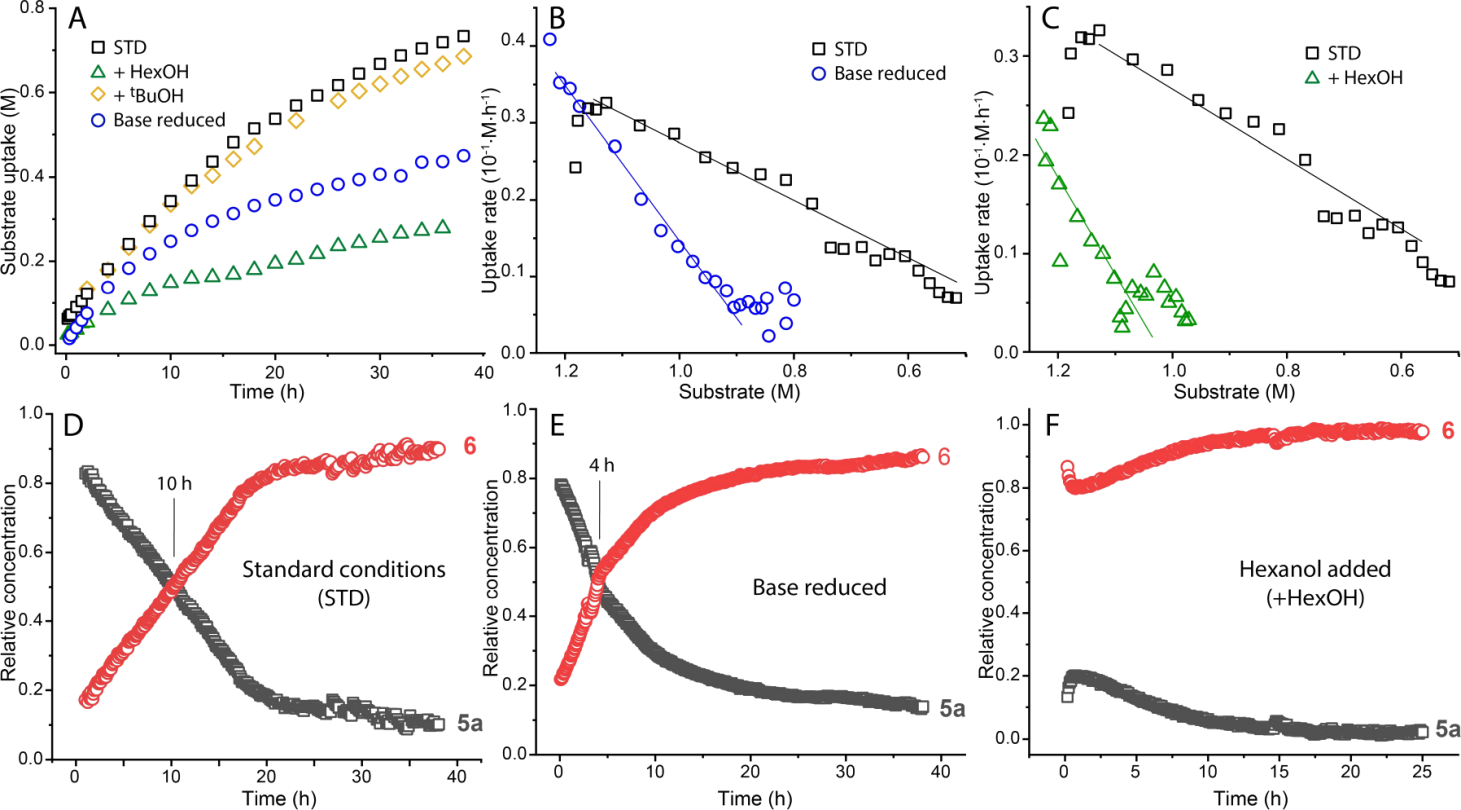
Summary data for kinetics of hexyl hexanoate hydrogenation (A) and hydrogenation rate plots (B,C) under varying base and alcohol concentrations and the operando IR spectroscopy traces (D–F) indicating the extent of product inhibition. Conditions—*standard*: hexyl hexanoate (1.25 M), catalyst **2** (0.1 mol %), KO^*t*^Bu (10 mol %) in THF (8.2 mL), 70 °C, 40 bar H_2_; *reduced base:* KO^*t*^Bu loading lowered to 2 mol %; *hexanol/^t^BuOH added*: extra alcohol added at 1.25 M. *Notes:* ester uptake data in A–C determined by GC analysis, relative concentrations in D–F obtained from FTIR spectroscopy.

To probe the presence of deactivation, we monitored the reaction progress with simultaneous spectroscopic analysis of the reaction mixture composition with IR spectroscopy. A typical dataset produced in this study is depicted in Figure 4 that presents a detailed overview of our assignments. Examining the evolution of the carbonyl ligand bands of **2** in the course of reaction we note that the reaction onset is marked by the fast establishment of the amido complex **5a** as the major species in the reaction mixture (Figure 4). As the hydrogenation progressed and the alcohol product was formed, we observed a gradual consumption of **5a** and the formation of a new species **6** with ν(CO) = 1902 and 1806 cm^–1^ suggesting that **6** is a Mn dicarbonyl complex. Performing an ex-situ test to assign the structure of **6** we found that this complex is the product of the metal–ligand cooperative alcohol addition to the amido complex **5a** (Figure 4C). Using methanol as a model alcohol we could obtain reference Fourier transform infrared (FTIR) and NMR spectra for the alkoxide **6** and establish the reversibility of its formation with the alkoxide being favored at low temperature and the amido complex **5a** favored at elevated temperatures (see Figures S24–27). We assigned an inhibitory role to the alkoxide complex **6** based on our real-time FTIR data (Figures 4 and 5) evidencing the drop in the overall catalytic hydrogenation rate that coincides with the accumulation of **6** (Figure 4B). In particular, product inhibition manifests as the increase of alcohol product concentration leads to the consumption of the kinetically competent species **5a**. We note that the formation of alkoxide complexes similar to **6** is common for metal-catalyzed (Ru, Fe, Os, and Mn) hydride transfer reactions, especially in acceptorless dehydrogenative coupling, although their involvement in catalysis remains under debate.^12^ Bergens and co-workers suggested that the Ru alkoxide could be the catalytically relevant intermediate formed through inner-sphere hydrogenation.^12c^ On the other hand, many authors including Gauvin, Mezzetti, and Morris proposed Mn alkoxide complexes either as off-cycle intermediates or resting states that cause lower reactivity.^12g,12^ The most detailed analysis to date was reported by the Saouma’s group who investigated the relevance of the alkoxide complexes to the hydrogenation catalysis.^12h^ The authors directly measured the equilibria of the formation of Ru alkoxide and concluded the latter to compete with the H_2_ addition to the Ru amido complex.

By performing hydrogenation in the presence of the hexanol ([HexOH]_0_ = 1.25 M at *t* = 0) we confirmed the inhibitory nature of alcohol binding to **5a**. The addition of alcohol strongly impacted the catalytic performance and the reaction mixture composition (Figure 5). First, we observed Mn alkoxide **6** to become the dominant Mn species from the onset of the reaction (Figure 5D vs F). Second, we detected a significant drop in the hydrogenation rate compared to the standard hexanol-free runs at the same substrate concentration (Figure 5C). These experiments confirm the detrimental impact of the product formation on catalysis and constitute a typical case of product inhibition. Interestingly, bulky and less acidic *tert*-butanol (see Figures 5A and S37–38) did not notably inhibit catalysis as suggested by the absence of reactivity of this alcohol with **5a** that we observed ex-situ. In line with the literature discussed above, our data for the Mn-CNC provides spectroscopic and kinetic support to the notion that alcohol adducts are detrimental for catalysis and might either be off-cycle or resting species in this transformation as reported previously.^12k,12l^
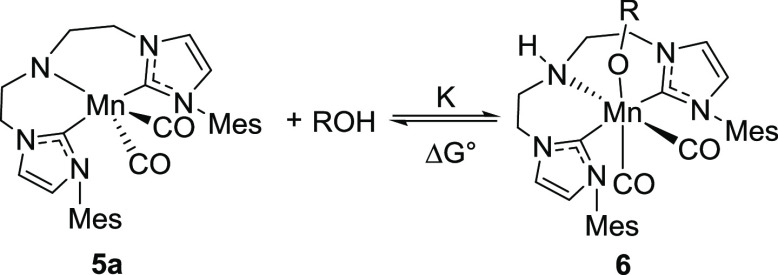
1
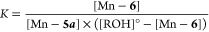
2
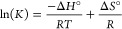
3
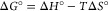
4

**Figure 6:**
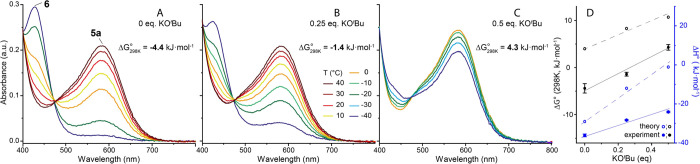
Ultraviolet (UV)–vis spectra describing the dependence of the equilibrium of **5a**–**6** (eq 1) on the concentration of KO^*t*^Bu in THF. Comparison between mixtures of **5a** (0.567 mM) and (A) hexanol (55.6 mM), (B) hexanol (113.4 mM) and KO^*t*^Bu (28.35 mM, 0.25 equiv) and (C) hexanol (113.4 mM) and KO^*t*^Bu (56.7 mM, 0.5 equiv). Reaction free energy (Δ*G*) for reversible alkoxide formation given in A–C and plotted together with theoretical data in panel D (COSMO-RS//PBE0-D3(SMD_THF_)/6-311++G(d,p), see Section S9.5 of Supporting Information for details).

As the reactivity of alcohols strongly depends on their acidity, we expected the magnitude of the inhibition effect to depend on the alcohol in question. Complex **5a** has blue color and a characteristic absorbance peak at 583 nm while its alcohol adduct **6** has a distinct feature at 428 nm (see Section S8 of Supporting Information and Figure 6A for representative spectra). Monitoring the equilibrium between **5a** and **6** (eq 1) with UV–Vis spectroscopy, we could track the temperature dependence of the equilibrium constant (eq 2) and by extension obtain the estimate of reaction free energy Δ*G*_298*K*_^°^ (eqs 3 and 4) for this transformation.

We determined thermodynamic parameters of the reversible alcohol addition for three representative alcohols—*n*-hexanol, methanol, and benzyl alcohol (see Table S8). The results confirmed that more acidic alcohols, Me- and BnOH bind more favorably than hexanol with the measured Δ*G*_298*K*_^°^ being −13.5 and – 11.3 kJ/mol for MeOH and BnOH, respectively, compared to −4.4 kJ/mol for hexanol. These data mimic the trends that we observed in our substrate scope screening (see Table S2) where methyl and benzyl esters consistently provided lower hydrogenation yields compared to their long chain counterparts (substrates **A7**, **A8**, and **A9**, Section S6 of Supporting Information). Similarly, the hydrogenation of aromatic esters (**A7**–**A10**) that produces benzyl alcohol as one of the products resulted in lower yields compared to the aliphatic esters (**A1**–**A6**) despite the latter being less electrophilic and less susceptible to the hydride transfer reactions often invoked as the first step in ester hydrogenation. These observations suggest that the product-induced inhibition might direct the performance of a large number of hydrogenation catalysts or at least impact their productivity. Similar trends favoring esters producing less acidic alcohols upon hydrogenation have been observed in Ru-catalyzed hydrogenations.^5b,5,13^ Importantly, these findings suggest that the outcome of ester hydrogenation is not only defined by the substrate reactivity, but also the capacity of reaction products to inhibit catalysis.

### Base Effects on Inhibitory Equilibria

At this point, we were met with contradiction arising from the UV–Vis data describing the equilibrium between **5a** and **6** in THF. The measured negative Gibbs free energy change implied that in the presence of hexanol, hydrogenation would be strongly inhibited at all times during catalysis that was not the case according to the operando IR data depicted in Figure 5D–F. We assumed that the presence of the alkoxide bases might affect the catalyst inhibition and extended the catalyst lifetime. To probe this, we extended the temperature dependent UV–vis spectroscopy studies to track the **5a**–**6** equilibrium in the presence of the KO^t^Bu additive. As noted by Kempe and co-workers, a superstoichiometric amount of the base may promote further deprotonation of the neutral Mn alkoxide.^7n,14^ We additionally verified that **6** cannot convert in the same manner using NMR spectroscopy where a proton resonance of the N–H group of **6** can be observed even in the presence of manifold excess of KO^t^Bu (see Figures S28–30). Strikingly, we found that the addition of substoichiometric amounts of KO^*t*^Bu with respect to alcohol significantly impacts the equilibrium and catalyst speciation in Mn/alcohol mixtures.

This translates to a substantial change of the standard Gibbs free energy for the **5a–6** transformation (Figure 6) although the base promotor is not involved in this equilibrium directly. Compared to the case of the pure **5a**/alcohol system showing a negative Δ*G*_298*K*_^°^ of −4.4 kJ·mol^–1^, the addition of 0.25 equivalents of KO^t^Bu with respect to the alcohol elevates the Gibbs free energy by approximately 3 kJ·mol^–1^ to −1.4 kJ·mol^–1^, which increases further to 4.3 kJ·mol^–1^ upon the elevation of the base contents to 0.5 equivalents (Figure 6). These values remain valid even when we incorporate a likely exchange reaction between *tert*-butoxide and free hexanol in our calculation. Such a correction affects the obtained Δ*G*_298*K*_^°^ values by no more than 1 kJ·mol^–1^ suggesting a large magnitude of the alkoxide addition effects in perturbing the equilibria responsible for the catalyst inhibition. Since the addition of alkoxide bases affects the catalyst speciation, it also has a direct impact on catalysis. The operando IR follow-up of the ester hydrogenation confirmed that the inhibition onset in hydrogenations with reduced base loading (Figure 5D vs E) occurs at lower alcohol concentrations, while higher base loadings allow for delaying this inhibition and extending the catalyst lifetime.

**Figure 7:**
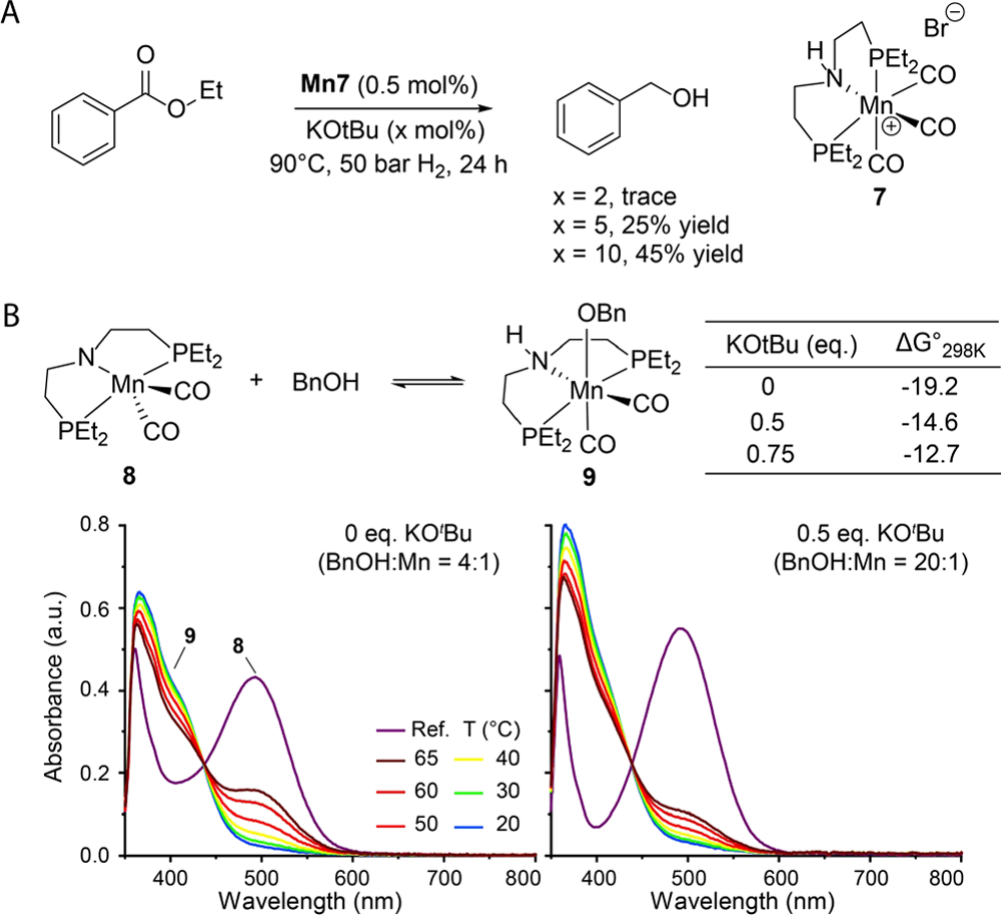
Ester hydrogenation with Mn-PNP **7** (A) and thermodynamic analysis for alcohol addition to this complex (B). Hydrogenation conditions: ethyl benzoate (1.25 mmol), **7** (0.5 mol %), KO^*t*^Bu, THF (0.5 mL), 50 bar H_2_, 90 °C, 24 h. See Section S8 in Supporting Information for UV–vis spectroscopy conditions.

We expected our findings to be general since the alcohol addition to the amido pincers is a common reaction and can affect the performance of many catalysts. Indeed, the identical effect of the base promotor can be found for other Mn catalysts, e.g., Mn-PNP (**7**) pincer reported recently by Beller and co-workers (Figure 7).^8a^ We found that increasing the base loading from 2 to 10 mol % could gradually improve the performance of Mn-PNP (**7**) catalyzed ethyl benzoate reduction with alcohol yields rising from <1 to 45% (Figure 7A). This improvement in the catalytic performance can be traced back to the same inhibitory process as we observed for Mn-CNC. Namely, deprotonated Mn-PNP amido complex **8** was found to bind benzyl alcohol forming the corresponding adduct **9**. The changes to the base promotor concentration strongly affected the standard Gibbs free energy change Δ*G*_298*K*_^°^ of this transformation (Figure 7B) and impacted the catalyst speciation dramatically. Similar to the case of Mn-CNC, the data depicted in Figure 7 highlight that Mn-PNP largely exists in the inhibited form throughout catalysis.

Taking our data together, we conclude that standard thermodynamic parameters universally assumed constant are, in fact, condition dependent. Interestingly, this dependence can be reflected on the level of theory when examined using DFT calculations. In particular, we utilized the COSMO-RS method that allows calculating chemical potentials and their concentration dependences in real solutions based on the DFT data (see Section S9.4 for the detailed methodology and results).^15^ We analyzed the condition-dependencies of the thermodynamics of alkoxide formation in the presence of a base and found that the addition of an alkoxide base can indeed affect the standard thermodynamic constant Δ*G*^°^ of the reaction, which does not formally involve this base as a reactant. The magnitude of this effect is sufficient to perturb the reaction Gibbs free energy change by 4–8 kJ·mol^–1^ in line with our experimental observations depicted in Figure 6. We found that the addition of the base mainly affects the chemical potential of the alcohol component rather than the metal complexes (see Table S15). Indeed, in aprotic solvents, one would expect the alcohol component to be affected stronger by the interaction with ionic alkoxide bases thus making this behavior sensible from the molecular standpoint. Nevertheless, the magnitude of this effect and its impact on catalysis are novel and entirely unexpected.

## Conclusions

In summary, this work describes two features of early metal-based catalysts that have profound influence on the outcome of the catalytic ester hydrogenation. First is the pronounced product inhibition developing throughout catalysis, caused by the reversible binding of the alcohol product to the catalyst. Demonstrating its capacity to severely diminish the steady state concentration of the catalytically competent species, we expect this inhibitory pathway to be highly relevant for early transition metal catalysts that tend to form more stable alkoxide complexes compared to their noble metal counterparts. The case of manganese pincers demonstrates that even at a low reaction extent, these well-defined complexes largely exist in an inhibited state if no base promotor is used.

More importantly, we found that common alkoxide bases can counter this by affecting the inhibitory equilibrium and its standard thermodynamic parameters. While the latter is often assumed to be ironclad, we show that the thermodynamic favorability of steps in a catalytic cycle is defined by the reaction medium and can be tuned by promotors and additives that do not participate in any specific chemical transformation. We, therefore, stress the necessity to view promotors as an integral component of the reaction medium rather than a stoichiometric reagent.

Finally, we conclude by noting that complexity uncovered in this work can impact any catalytic transformation involving reversible alcohol binding. Having demonstrated the generality of our findings for Mn-catalyzed hydrogenations, we expect that the rational use of promotors can become a powerful tool for designing catalytic reactions where the favorability of elementary steps is no longer a perceived constant, but can be tuned and manipulated at will.

## Supporting Information Available

Full synthetic procedure descriptions, characterization data, and catalytic test methodology (PDF)
Chemical structures of computed complexes (ZIP)

## Accession Codes

CCDC 2099206–2099208 contain the supplementary crystallographic data for this paper. These data can be obtained free of charge via www.ccdc.cam.ac.uk/data_request/cif, or by emailing data_request@ccdc.cam.ac.uk, or by contacting The Cambridge Crystallographic Data Centre, 12 Union Road, Cambridge CB2 1EZ, UK; fax: +44 1223 336033.
