# Supplementary material for: Basic Promotors Impact Thermodynamics and Catalyst Speciation in Homogeneous Carbonyl Hydrogenation
Source: J Am Chem Soc. 2022 Apr 27;144(18):8129–37. doi: 10.1021/jacs.2c00548 (PMC9100671; doi:10.1021/jacs.2c00548)
Supplement: Supplementary file 1 — ja2c00548_si_001.pdf [file ja2c00548_si_001.pdf]

SUPPORTING INFORMATION

Basic Promotors Impact Thermodynamics and Catalyst Speciation in Homogeneous Carbonyl Hydrogenation

Wenjun Yang,[a] Tejas Y. Kalavalapalli,[a] Annika M. Krieger,[a] Taras A. Khvorost,[b] Ivan Yu. Chernyshov,[b] Manuela Weber,[c] Evgeny A. Uslamin,[a] Evgeny A. Pidko,*,[a] Georgy A. Filonenko*,[a]

[a] Inorganic Systems Engineering group, Department of Chemical Engineering, Faculty of Applied Sciences, Delft University of Technology, Van der Maasweg 9, 2629 HZ, Delft, The Netherlands

[b] TheoMAT Group, ChemBio cluster, ITMO University, Lomonosova 9, St. Petersburg, 191002, Russia

[c] Institute of Chemistry and Biochemistry, Freie Universität Berlin, Fabeckstraße 34/36, Berlin, D-14195, Germany

Corresponding authors: Georgy A. Filonenko (G.A.Filonenko@tudelft.nl)

Evgeny A. Pidko (E.A.Pidko@tudelft.nl)

Dataset for this publication is available from 4TU.Research data under DOI: 10.4121/19323839

Contents

S1 – General Considerations 3

S2 – Synthetic Procedures and Characterization Data. 4

S3 –Activation Study. 18

S4 – Catalytic Hydrogenation Details. 25

S5 – Optimization of Reaction Conditions. 27

S6 – Substrate scope 28

S7 – *Operando* spectroscopy studies. 29

S8- UV-vis Studies 35

S9 – Computational Studies. 44

S9.1 Interconversion of polycarbonyl Mn(CNC) complexes 45

S9.2 Conformational screening and assignment of the deprotonated Mn$(CO)_2$CNC isomers 46

S9.3 The interaction of deprotonated Mn$(CO)_2$CNC with alkoxide base and assignment of 5b 48

S9.4 Comparison of experimental and calculated spectra. 50

S9.5 Analysis of base effects on chemical potentials of components of inhibitory equilibrium 52

S10 – Crystal Structure Analysis Details. 56

S11 – References. 58

S1 – General Considerations

All manipulations were, unless stated otherwise, performed under inert atmosphere in an argon filled glovebox (INERT) or using standard Schlenk techniques. Anhydrous solvents were either dispensed from an Inert PureSolv solvent purification system or dried using 3/4 Å molecular sieves and were degassed before use. Chemicals were purchased from Sigma-Aldrich, Strem, abcr, or TCI. Liquid hydrogenation substrates were degassed and dried by molecular sieves before use. Air and/or moisture sensitive materials were stored in the glovebox. Deuterated solvents were purchased from Eurisotop, dried using molecular sieves, degassed and stored in the glovebox.

NMR spectra were recorded on an Agilent 400-MR DD2 400 MHz spectrometer equipped with a 5 mm ONE NMR probe. All ^{13}C and ^{31}P NMR spectra were recorded with ^{1}H decoupling. All chemical shifts were referenced to residual solvent peaks [$CDCl_3$: 7.26 ppm (^{1}H), 77.2 ppm (^{13}C); $(CD_3)_2SO$: 2.50 ppm (^{1}H), 39.5 ppm (^{13}C); THF-*d8*: 1.72/3.58 ppm (^{1}H), 67.6/25.4 ppm (^{13}C)]. Proton and carbon assignments were made on basis of combined gCOSY and gHSQC spectra. FTIR (ATR and transmittance modes) was measured on a Bruker Alpha II spectrometer. The solutions of the complexes in THF (0.05 M) were filled in an IR-cell with two CaF_2 windows and an optical path length of 0.1 mm which was purged with nitrogen prior to use. The cell was stabilized at 25 °C. The spectra were taken with a resolution of 2 cm^{-1}. Elemental analyses were performed by Mikroanalytisches Laboratorium Kolbe, Oberhausen, Germany.

S2 – Synthetic Procedures and Characterization Data.

Synthesis of Ligand 1[1]:

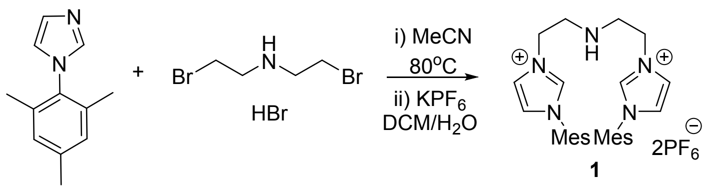

In a Schlenk tube was prepared suspension of bis(2-bromoethyl)amine hydrobromide (3.09 g, 10 mmol) in dry MeCN (15 mL). The mixture was heated to 80°C, and 5.58 gram of mesityl imidazole (30 mmol) was added by three portions in three hours. After heating overnight, cooled down the mixture and removed solvent on rotary evaporator. The residue was dissolved in water, neutralized with Na_2CO_3 till PH 9 (white solid precipitated out) and washed by diethyl ether for three times to recover unreacted imidazole. The aqueous phase was stirred vigorously with KPF_6 for 1 h to form a turbid emulsion followed by extraction with dichloromethane. Combined organic phases were dried over anhydrous Na_2SO_4 and evaporated to dryness to produce viscous oil. Co-evaporated the oil with toluene to afford dry product as white foam solid without further purification.

^{1}H NMR (400 MHz, $CDCl_3$, 297 K) δ 8.51 (s, 2H), 7.70 (d, J = 1.9 Hz, 2H), 7.12 (d, J = 1.9 Hz, 2H), 6.98 (s, 4H), 4.37 (t, J = 5.6 Hz, 4H), 3.18 (t, J = 5.6 Hz, 4H), 2.32 (s, 6H), 1.99 (s, 12H); ^{31}P{^{1}H}NMR (162 MHz, CD_2Cl_2, 297 K) δ -144.4 (hep, $^1J_{FP}$ 714.4 Hz); ^{13}C {^{1}H}NMR (101 MHz, $CDCl_3$, 297 K) δ 141.3, 136.3, 134.4, 129.8, 128.2, 123.7, 123.5, 49.8, 48.0, 21.1, 17.0; HRMS (m/z): $[M\text{-}_2PF_6]^{2+}$ Calcd. for $C_{28}H_{37}N_5^{2+}$, 221.6519; found 221.6515.

Synthesis of complex 2:

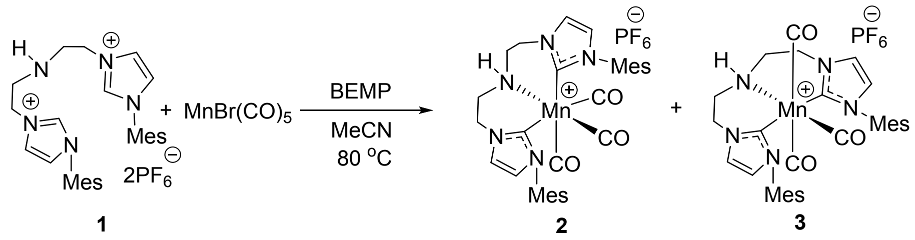

To the orange suspension of ligand **1** (733.0 mg, 1 mmol) and [$MnBr(CO)_5$] (275.0 mg, 1 mmol) in MeCN (5 mL) was added 2-tert-Butylimino-2-diethylamino-1,3-dimethylperhydro-1,3,2-diazaphosphorine solution (BEMP, 1M in hexane, 2.5 mL) and stirred at 80 °C for 24 h under Ar atmosphere and darkness. The resulting reaction mixture was then cooled down to room temperature and evaporated to dryness. The yellow solid residue was washed with MeOH to afford mixture of **2** (soluble in THF) and **3** (insoluble in THF). Complex **2** was fully extracted with 80 mL THF. The solution was filtered, evaporated to dryness and further purified by crystallization (MeOH vapor diffusion into solution in acetone) under darkness as light yellow solid in 15 % yield (108.5 mg). The remaining crude

3 was dissolved in minimal amount of DMSO, placed under light for a week, and further purified by crystallization (Et_2O vapor diffusion into solution in DMSO) as orange solid in 11 % yield (78.5 mg). Note: Complex **2** in solution was slightly sensitive to light and could be slowly isomerized to **3**, so the above workup should be performed immediately.

Complex **2**: ^{1}H NMR (400 MHz, DMSO-d_6, 297 K) δ 7.68 (d, J = 1.9 Hz, 2H, *CH*-imidazole), 7.26 (d, J = 1.8 Hz, 2H, *CH*-imidazole), 6.91 (s, 2H, C*H*-Mes), 6.81 (s, 2H, C*H*-Mes), 5.94 (s, 1H, N*H*), 4.36 (d, J = 15.1 Hz, 2H, CH_2-a), 3.78 (s, 2H, CH_2-a), 3.11 (d, J = 13.7 Hz, 2H, CH_2-b), 2.88 (s, 2H, CH_2-b), 2.22 (s, 6H, CH_3-Mes), 1.78 (s, 6H, CH_3-Mes), 1.40 (s, 6H, CH_3-Mes); ^{31}P{^{1}H}NMR (162 MHz, DMSO-d_6, 297 K) δ -144.2 (hep, $^{1}J_{FP}$ 711.2 Hz); ^{13}C {^{1}H}NMR (101 MHz,DMSO-d_6, 297 K) δ 218.7 (Mn-*C*O), 216.1 (Mn-*C*O), 187.7 (NHC Mn-*C*), 138.8 (CH*C*CH-f), 137.0 (C*C*C-c), 136.3 (CH*C*C-d), 135.7 (CH*C*C-d), 129.4 (C*C*HC-e), 129.1 (C*C*HC-e), 125.8 (*C*H-imidazole), 125.3 (*C*H-imidazole), 52.6 (CCH_2CH_2-b), 47.4 (CCH_2CH_2-a), 21.0 (CH_3-Mes), 18.0 (CH_3-Mes), 17.2 (CH_3-Mes); IR (solution in THF): ῡ [cm^{-1}] 2011 (s, ῡ CO), 1914 (s, ῡ CO), 1911 (s, ῡ CO); EA: Found (Calcd.) for $C_{31}H_{35}F_6MnN_5O_3$: C: 51.34 (51.32); H: 4.84 (4.86); N: 9.63 (9.65).

Complex **3**: ^{1}H NMR (400 MHz, DMSO-d_6, 297 K) δ 1H NMR (400 MHz, DMSO-d6) δ 7.66 (s, 2H, *CH*-imidazole), 7.18 (s, 2H, *CH*-imidazole), 6.91 (s, 4H, C*H*-Mes), 4.95 – 4.78 (m, 1H, N*H*), 4.41 – 4.26 (m, 2H, CH_2-a), 4.22 – 4.05 (m, 2H, CH_2-a), 3.15 – 2.96 (m, 2H, CH_2-b), 2.62 – 2.42 (m, 2H, CH_2-b), 2.23 (s, 6H, CH_3-Mes), 1.92 – 1.84 (m, 12H, CH_3-Mes); ^{31}P{^{1}H}NMR (162 MHz, DMSO-d_6, 297 K) δ -144.2 (hep, $^{1}J_{FP}$ 712.8 Hz); ^{13}C {^{1}H}NMR (101 MHz,DMSO-d_6, 297 K) δ 222.3 (Mn-*C*O), 216.4 (Mn-*C*O), 214.4 (Mn-*C*O), 189.4 (NHC Mn-*C*), 138.6 (CH*C*CH-f), 136.4 (CH*C*C-d), 136.2 (CH*C*C-d), 135.9(C*C*C-c), 129.0 (C*C*HC-e), 125.4 (*C*H-imidazole), 124.9 (*C*H-imidazole), 50.6 (CCH_2CH_2-b), 47.6 (CCH_2CH_2-a), 21.1 (CH_3-Mes), 18.3 (CH_3-Mes), 18.1 (CH_3-Mes); IR (solution in THF): ῡ [cm^{-1}] 2034 (s, ῡ CO), 1914 (s, ῡ CO), 1904 (s, ῡ CO); EA: Found (Calcd.) for $C_{31}H_{35}F_6MnN_5O_3$: C: 51.31 (51.32); H: 4.85 (4.86); N: 9.65 (9.65).

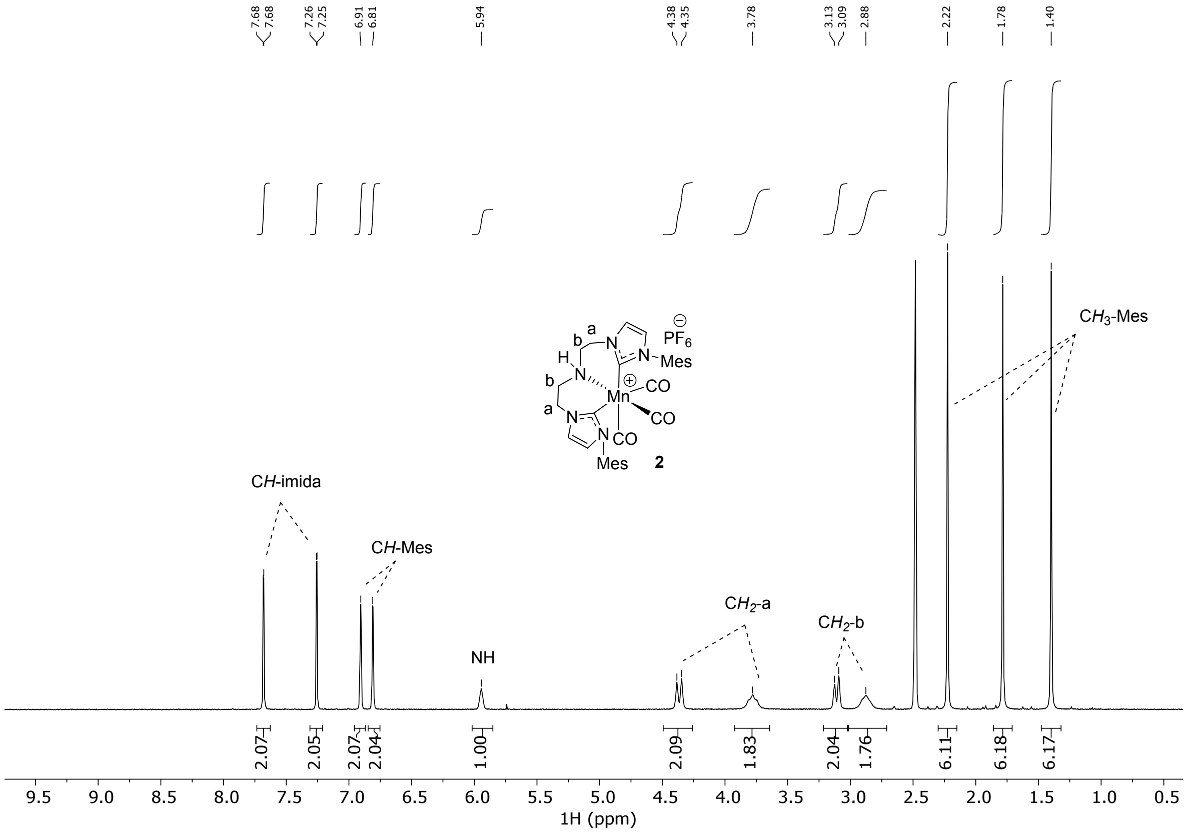

Figure S1. ^{1}H-NMR spectrum of complex **2** in DMSO-d$_6$ (400 MHz).

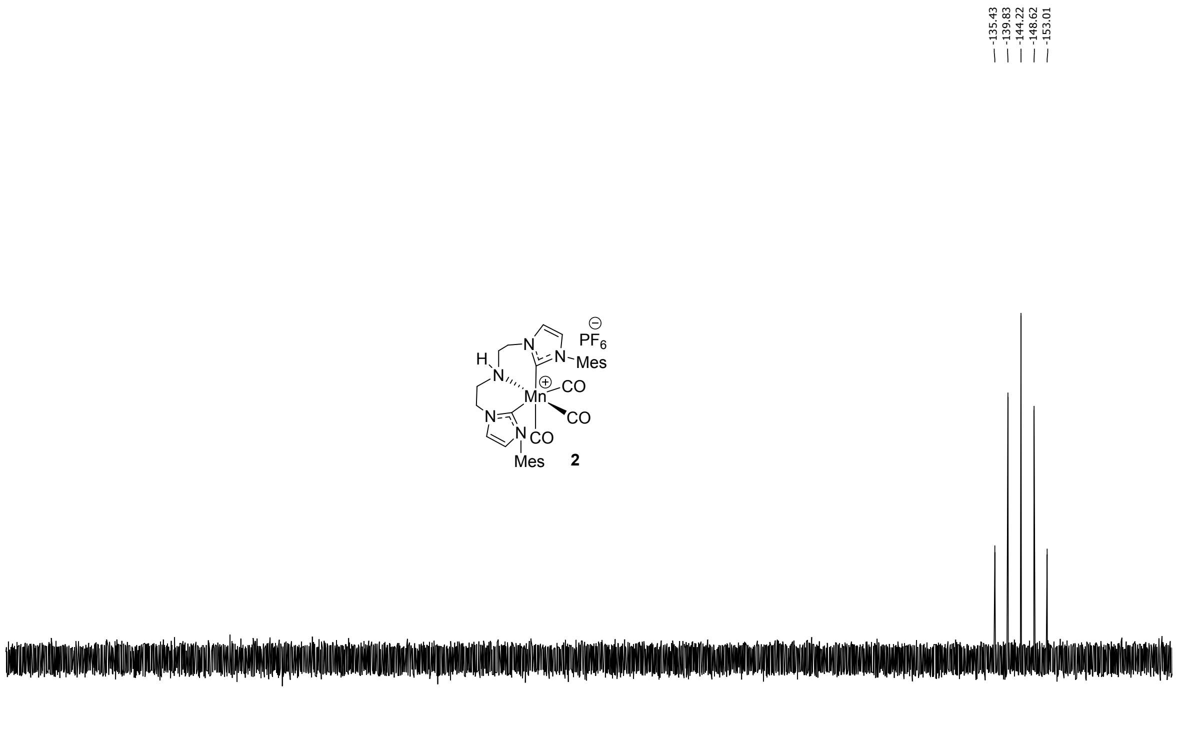

Figure S2. ^{31}P {^{1}H}NMR spectrum of complex **2** in DMSO-d$_6$ (162 MHz).

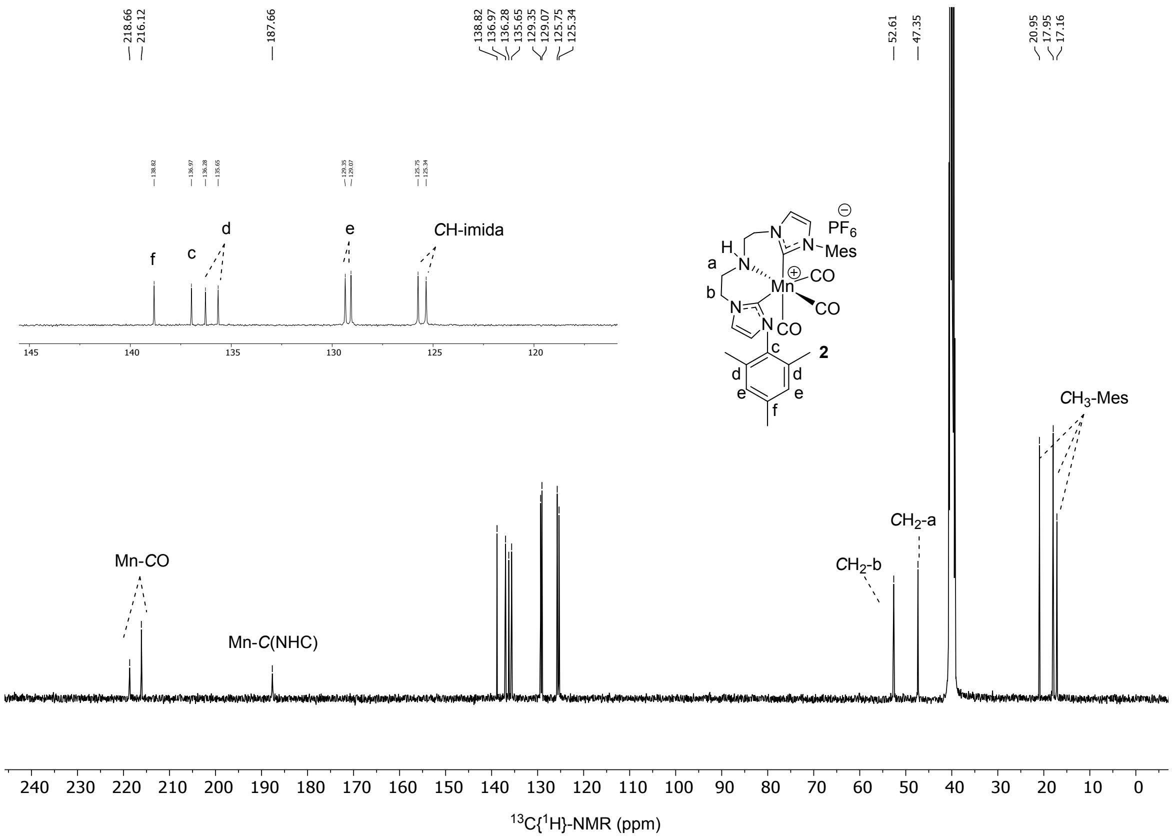

Figure S3. ^{13}C {^{1}H}NMR spectrum of complex **2** in DMSO-d$_6$ (100 MHz).

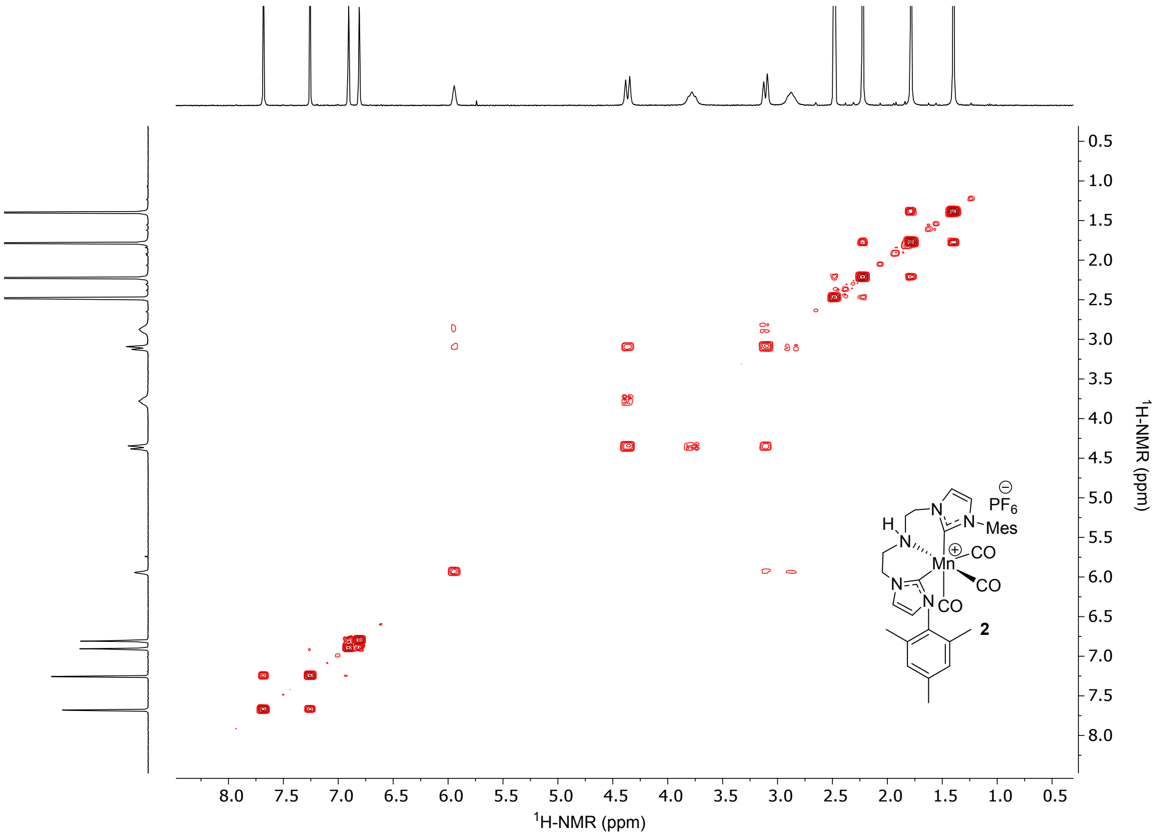

Figure S4. gCOSY spectrum of complex **2** in DMSO-d$_6$.

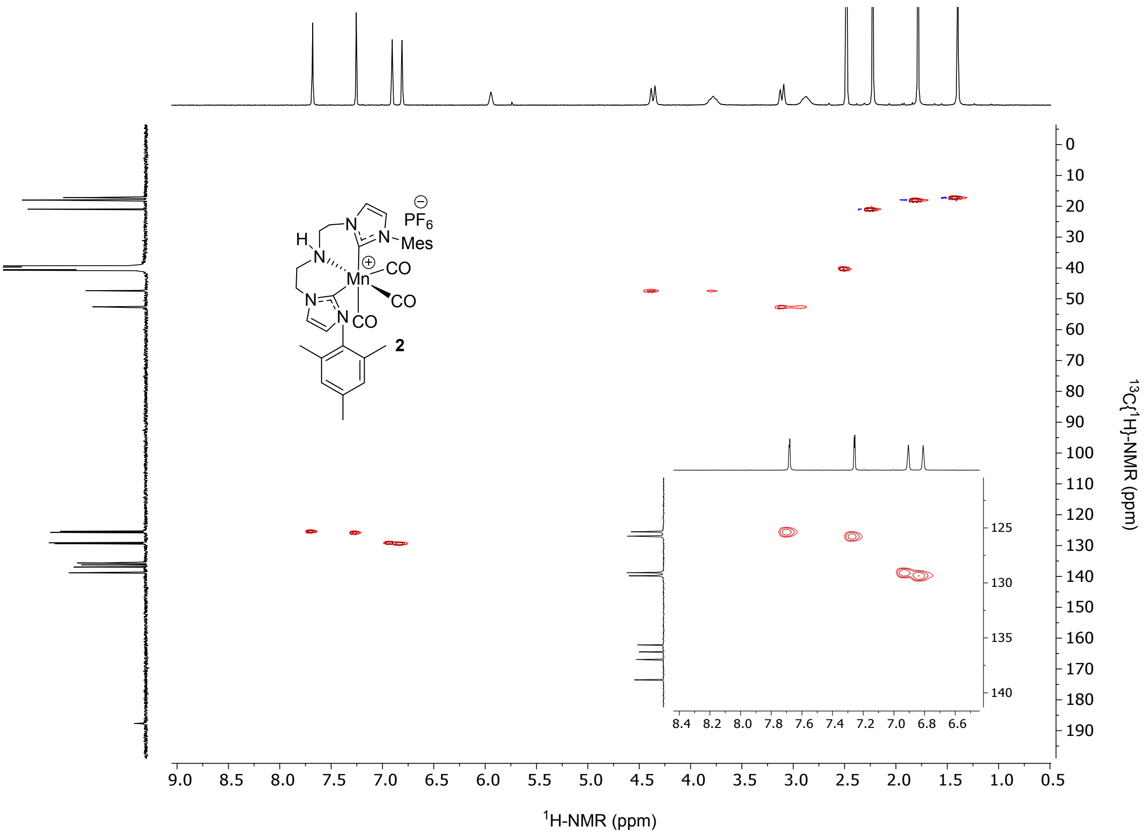

Figure S5. gHMQC spectrum of complex **2** in DMSO-d_6.

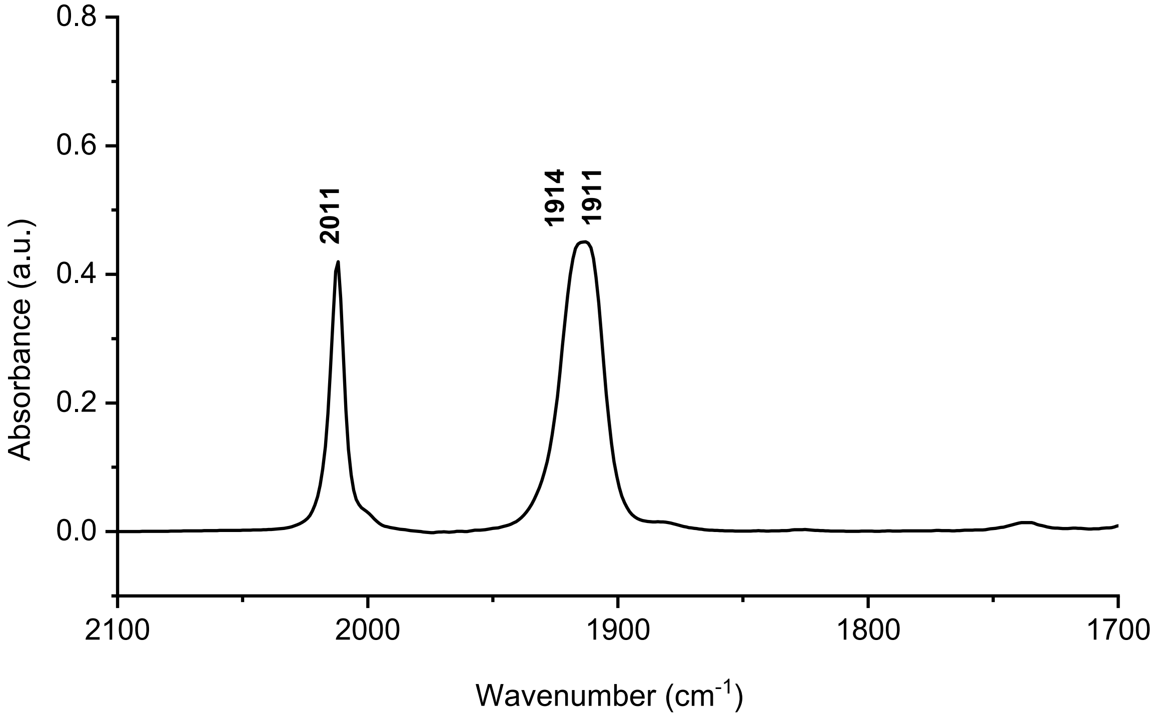

Figure S6. IR spectrum of THF solution of complex **2.**

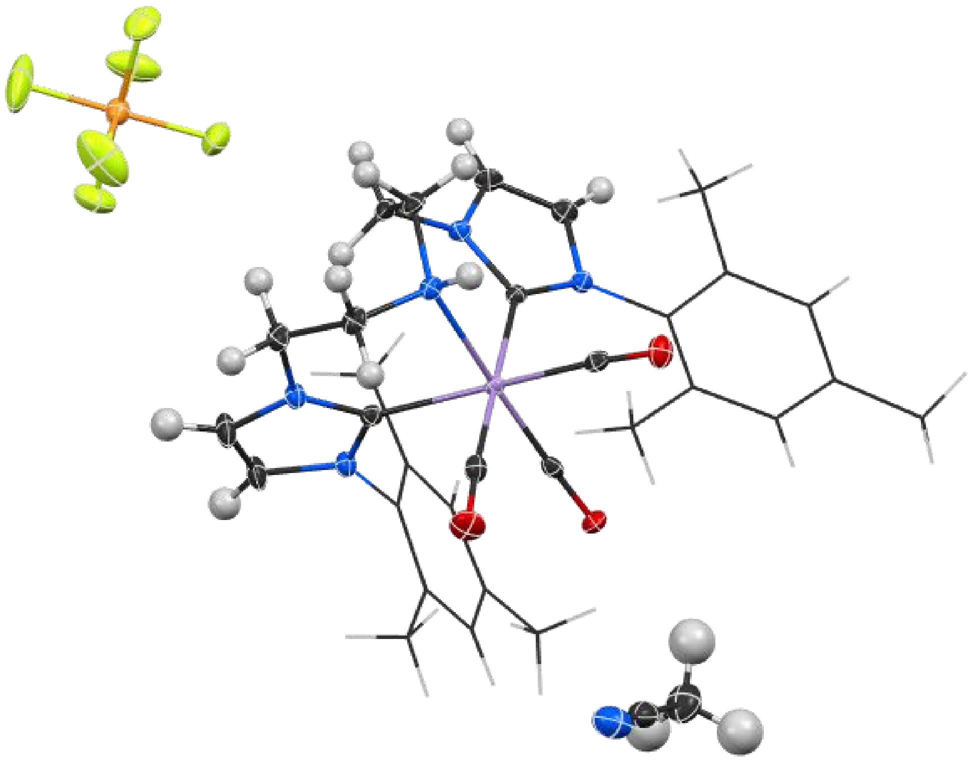

Figure S7. Molecular structure of complex **2** (co-crystallized with MeCN) in crystal with thermal ellipsoids drawn at 50% probability.

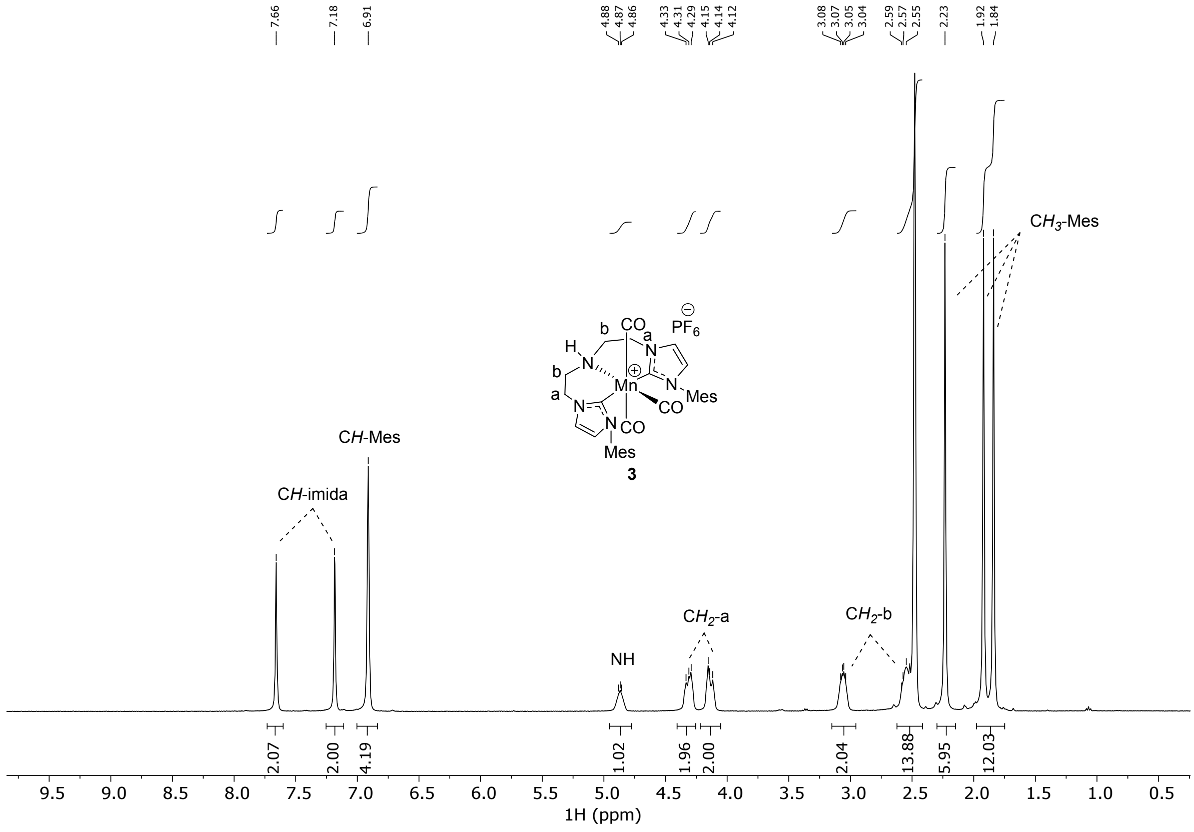

Figure S8. ^{1}H-NMR spectrum of complex **3** in DMSO-d_6 (400 MHz).

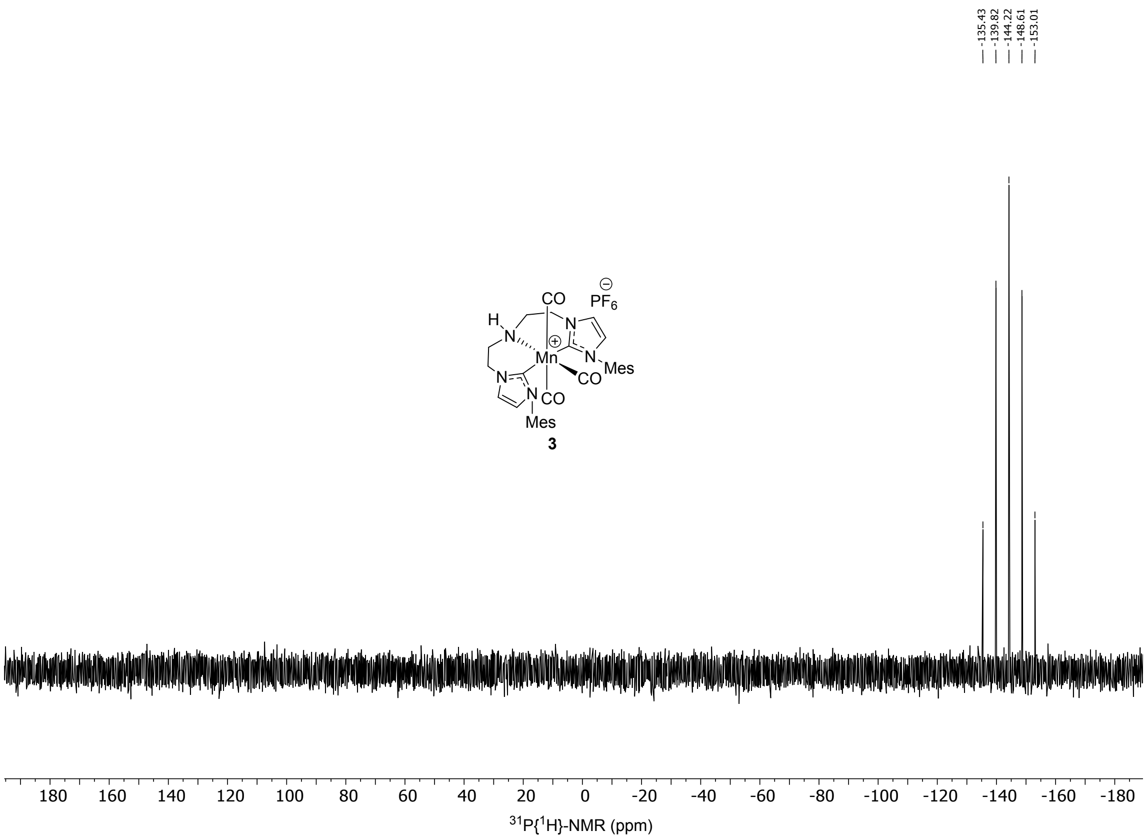

Figure S9. ^{31}P {^{1}H}NMR spectrum of complex **3** in DMSO-d$_6$ (162 MHz).

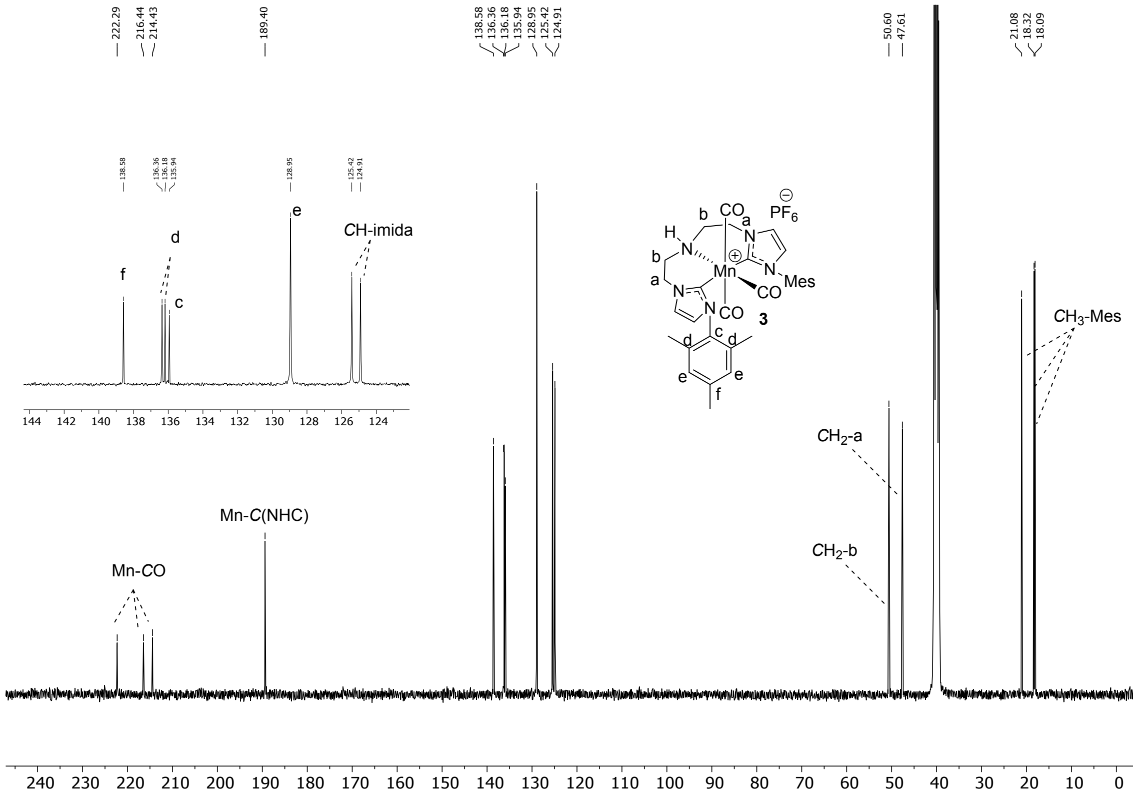

Figure S10. ^{13}C {^{1}H}NMR spectrum of complex **3** in DMSO-d$_6$ (100 MHz).

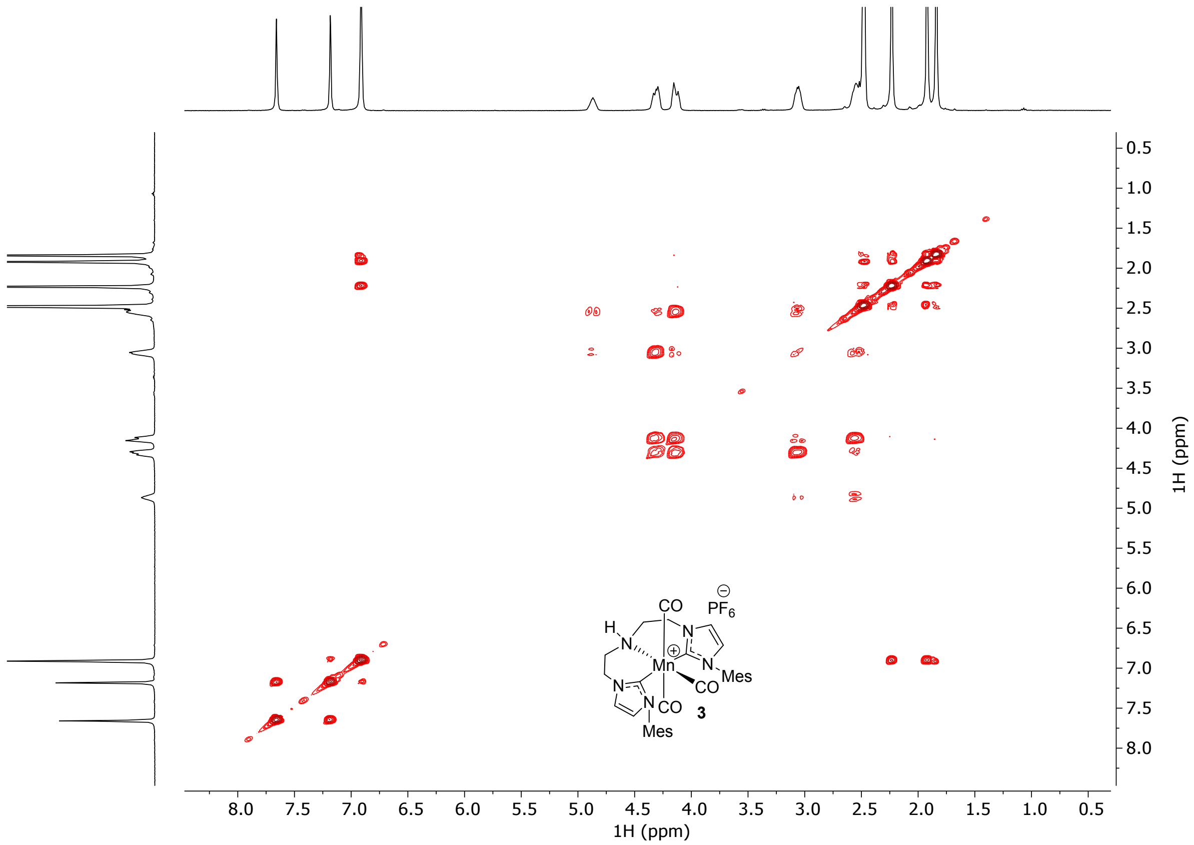

Figure S11. gCOSY spectrum of complex **3** in DMSO-d_6.

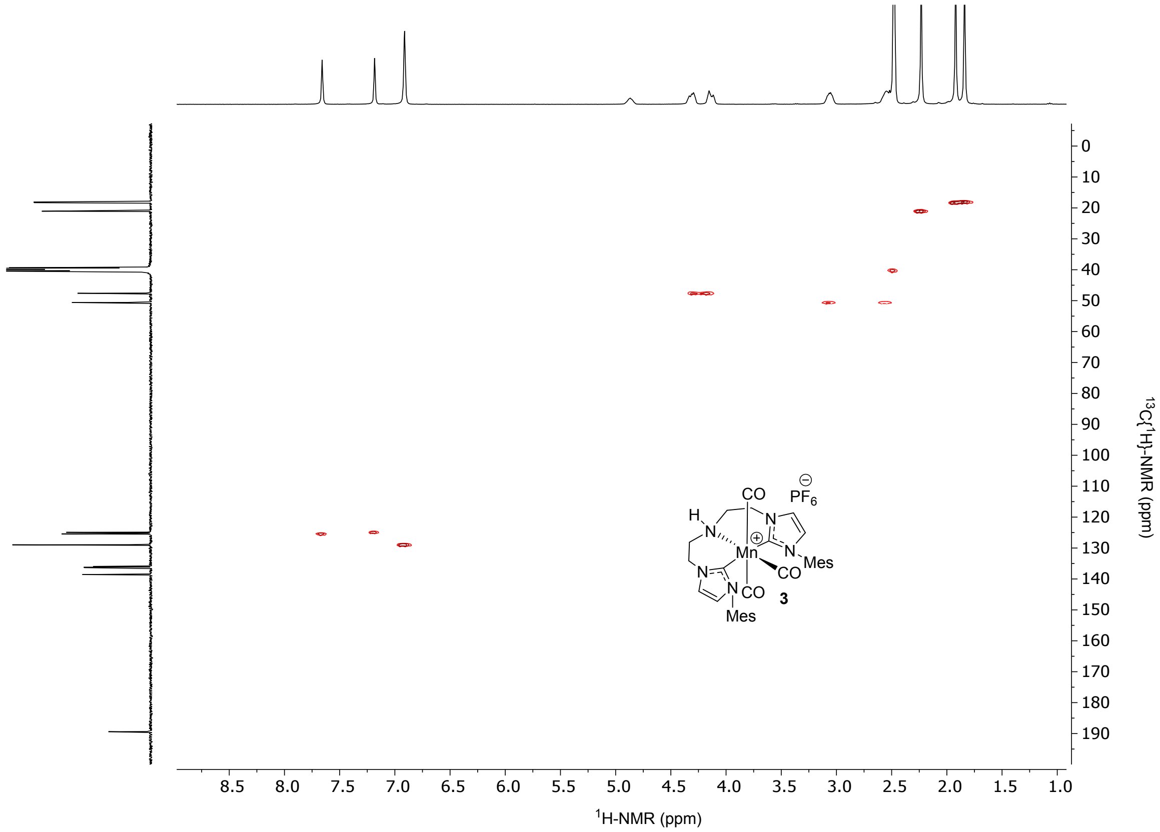

Figure S12. gHMQC spectrum of complex **3** in DMSO-d_6.

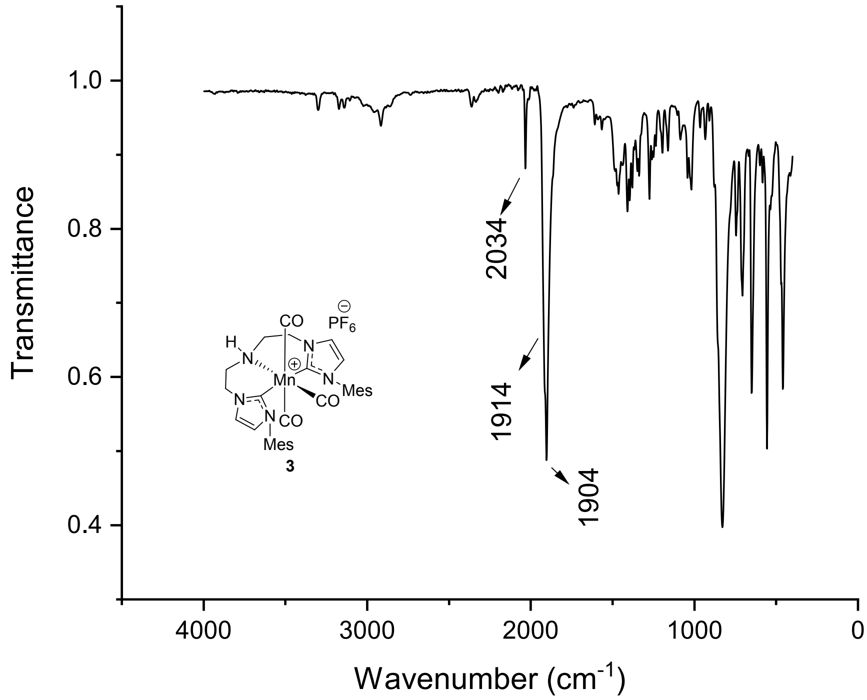

Figure S13. IR spectrum of complex **3**.

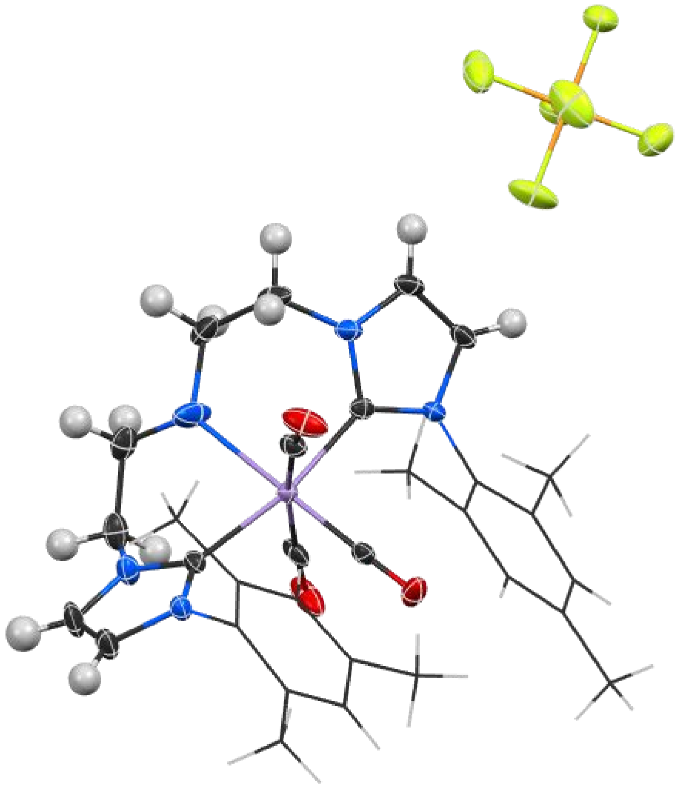

Figure 14. Molecular structure of complex **3** in crystal with thermal ellipsoids drawn at 50% probability.

Synthesis of complex 5a:

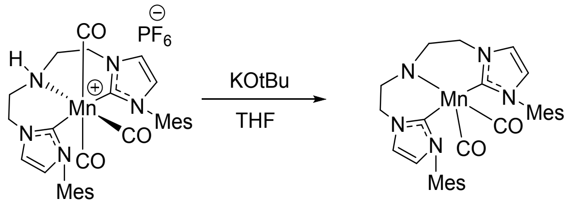

3 **5a**

To the solution of complex **3** (72.5 mg, 0.1 mmol) in THF (0.5 mL) was dropwise added KO^{t}Bu (11.8 mg, 0.105 mmol) in the mixture of 0.5 mL THF and 0.2 mL pentane. After stirring at rt for 1 h, the resulting mixture became dark blue and was filtered through a Celite plug to remove precipitation. The crude was further purified by slow diffusion of pentane into its solution in THF to afford **5a** as blue crystals in 45 % yield (24.6 mg).

^{1}H NMR (400 MHz, THF-d_8, 297 K) δ 7.19 (d, *J* = 1.8 Hz, 2H, C*H*-imidazole), 6.78 (s, 4H, C*H*-Mes), 6.73 (d, *J* = 1.8 Hz, 2H, C*H*-imidazole), 3.97 – 3.94 (m, 4H, CH_2-a), 2.88 – 2.86 (m, 4H, CH_2-b), 2.20 (s, 6H, CH_3-Mes), 1.90 (s, 12H, CH_3-Mes); ^{13}C {^{1}H}NMR (101 MHz, THF-d_8, 297 K) δ 236.2 (Mn-*C*O), 208.3 (NHC Mn-*C*), 137.8 (CH*C*CH-f), 136.8 (C*C*CH-d), 135.9 (C*C*C-c), 128.3 (C*C*HC-e), 120.9, 119.6 (*C*H-imidazole), 57.3 (*C*H_2-b), 52.1 (*C*H_2-a), 20.2, 17.4 (*C*H_3-Mes); IR (solution in THF): ῡ [cm^{-1}] 1886 (s, ῡ CO), 1811 (s, ῡ CO); EA: Found (Calcd.) for $C_{30}H_{34}MnN_5O_2$: C: 65.19 (65.33); H: 6.19 (6.21); N: 12.61 (12.70).

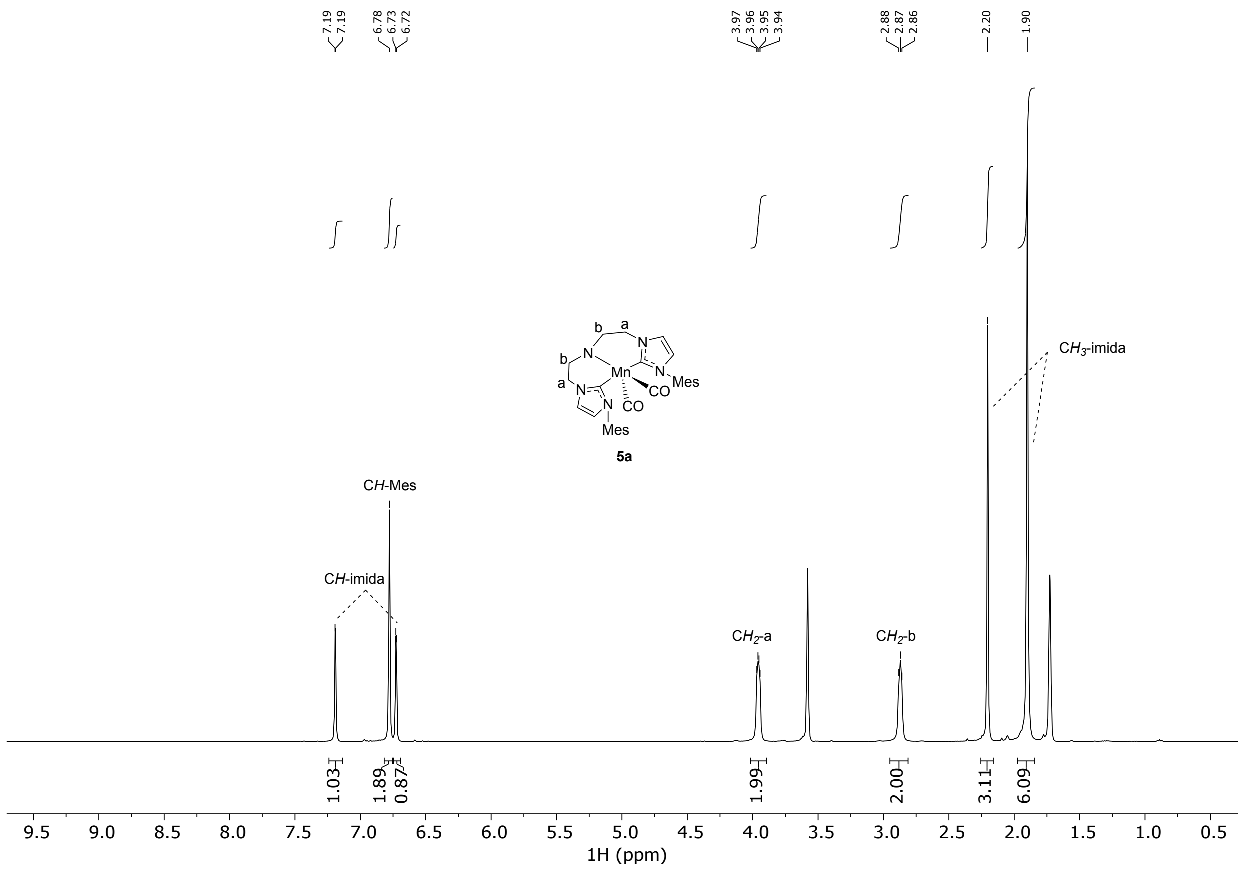

Figure S15. ^{1}H-NMR spectrum of complex **5a** in THF-d_8 (400 MHz).

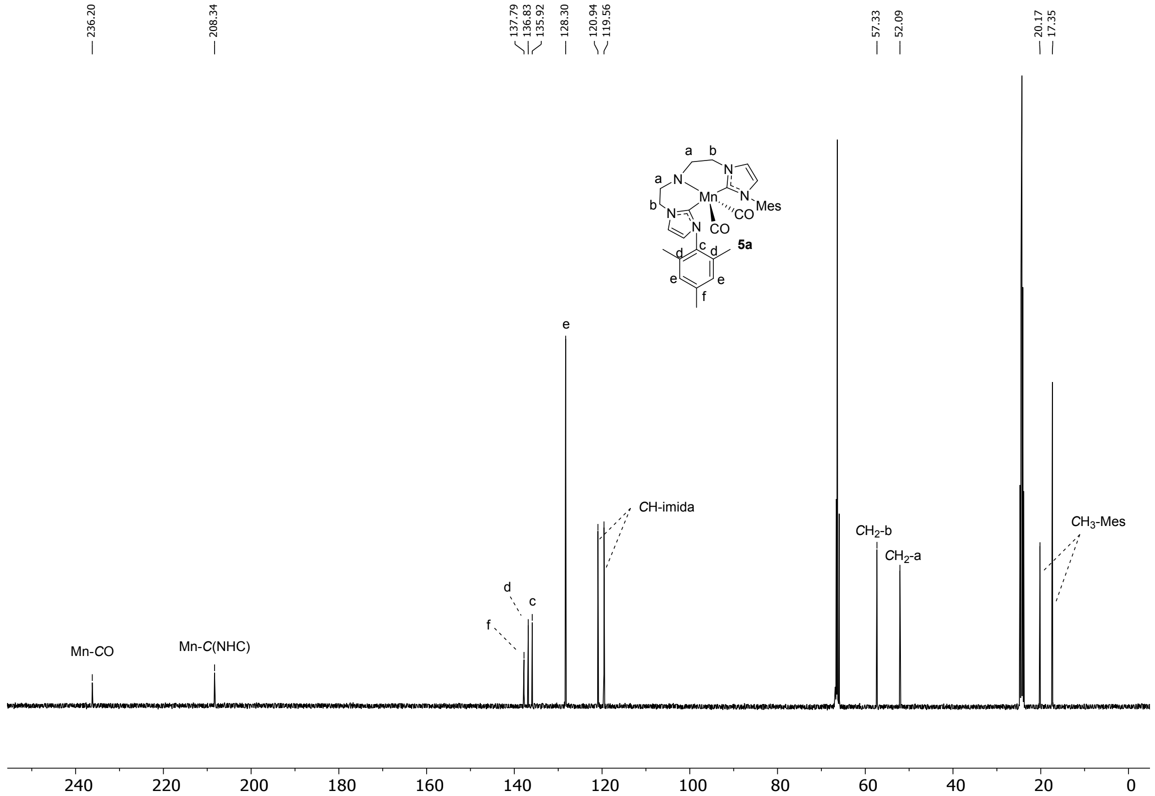

Figure S16.^{13}C {^{1}H}NMR spectrum of complex **5a** in THF-d_8 (100 MHz).

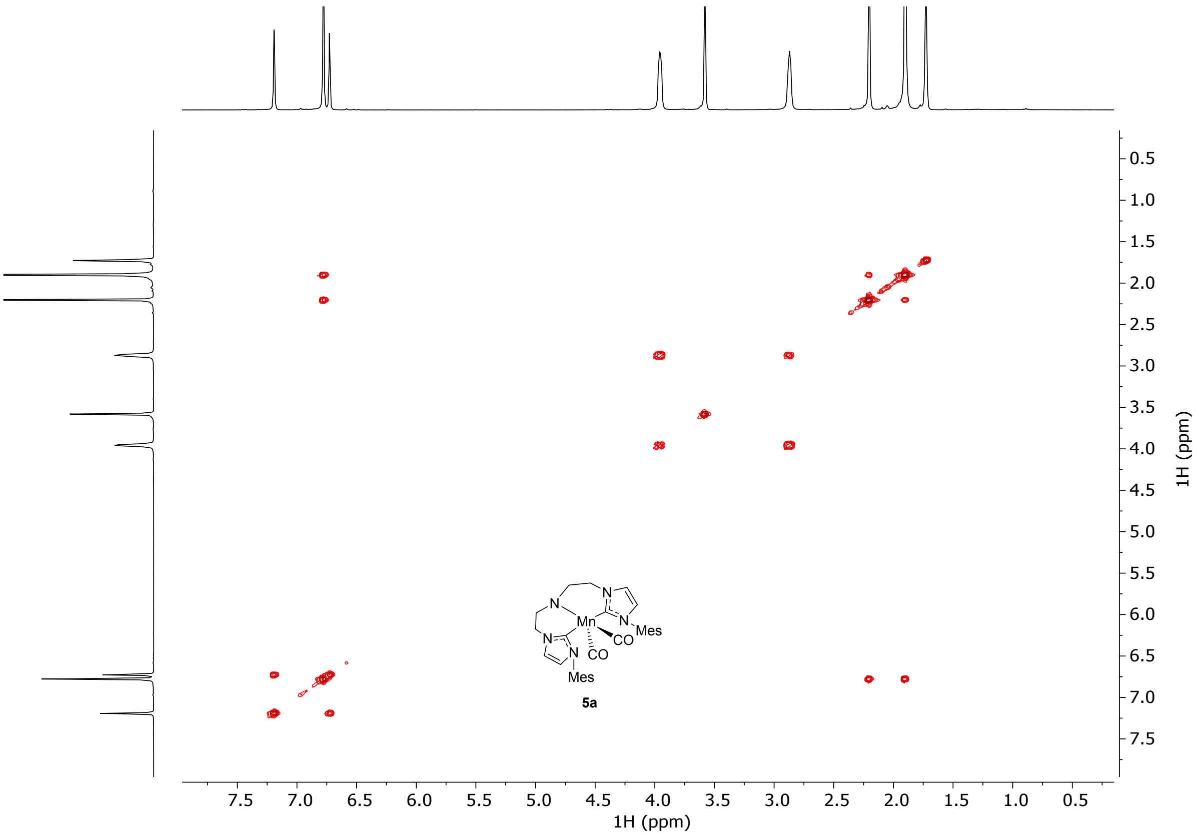

Figure S17. gCOSY spectrum of complex **5a** in THF-d_8.

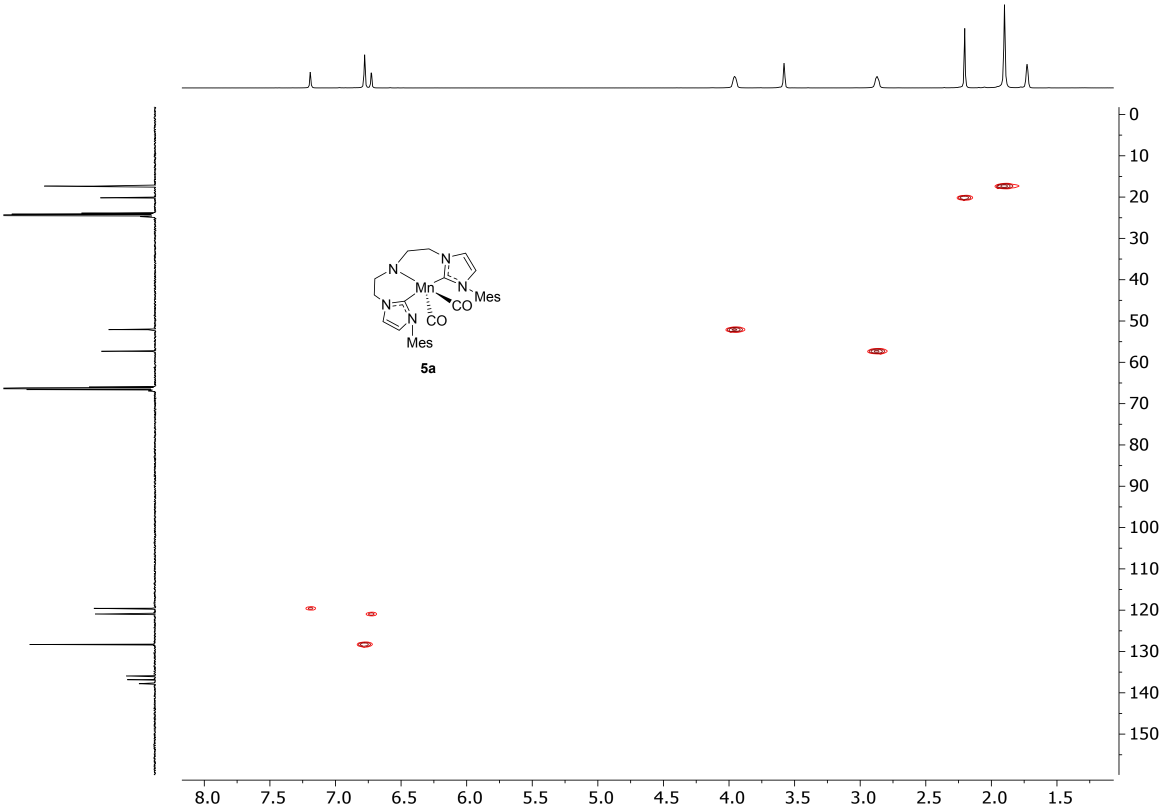

Figure S18. HMQC spectrum of complex **5a** in THF-d_8.

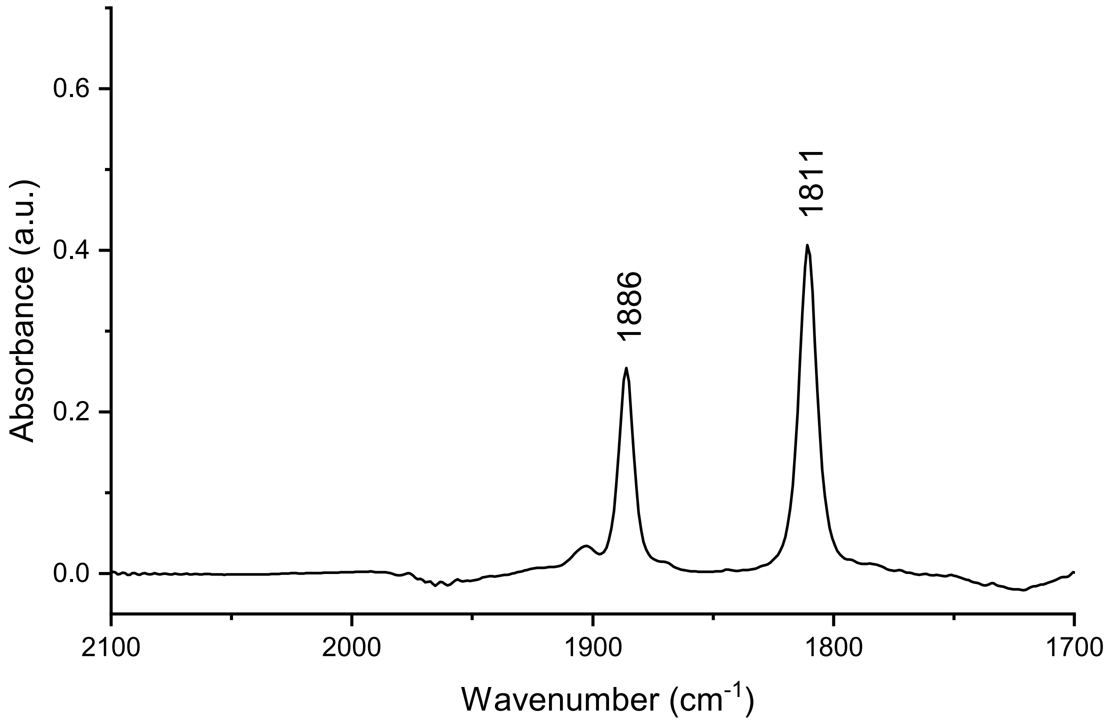

Figure S19. IR spectrum of THF solution of complex **5a.**

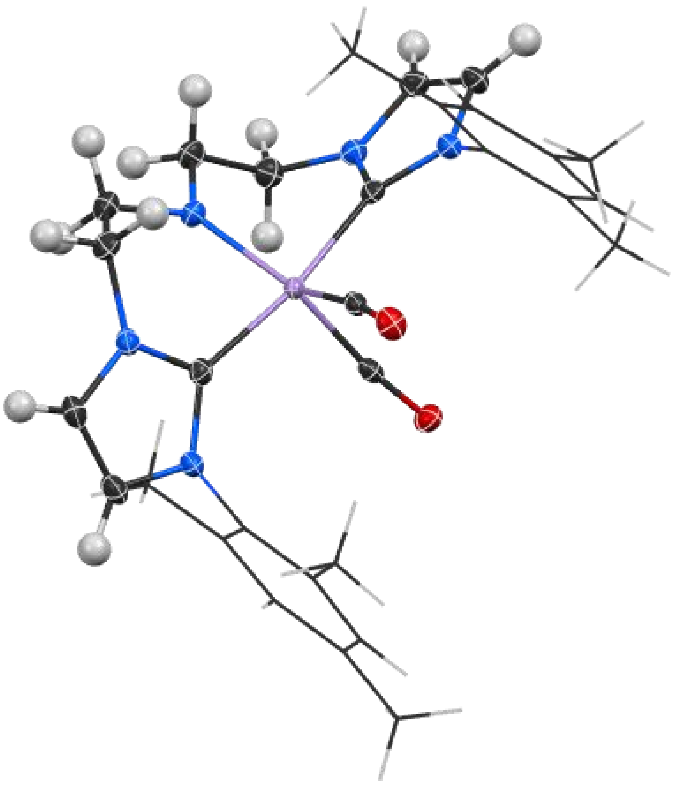

Figure S20. Molecular structure of complex **5a** in crystal with thermal ellipsoids drawn at 50% probability.

Synthesis of MnPNP complex 7 and 8:

The complex **7** and **8** were synthesized according to the procedure reported by Beller and co-workers.[2] To the orange suspension of [MnBr(CO)$_5$] (1 mmol) in toluene (5 mL) was added bis(2-diethylphosphinoethyl)amines (1 mmol) and stirred at 100 ^{o}C for 24 h under Ar atmosphere. The resulting reaction mixture was then cooled down to room temperature and evaporated to dryness. The yellow solid residue was washed with toluene several times and them dried under vacuum to afford complex **7** as pale yellow solid.

^{1}H NMR (400 MHz, 297 K, DMSO-d$_6$) δ 6.57 (s, 1H, N*H*), 2.84 – 2.55 (m, 4H, NCH_2), 2.24 – 1.82 (m, 12H, NCH$_2$CH_2P, PCH_2CH$_3$), 1.20 – 1.09 (m, 12H, PCH$_2$CH_3); ^{31}P{^{1}H} NMR (162 MHz, 297 K, DMSO-d$_6$) δ 62.5. The spectroscopic data consistent with literature.[2]

To the yellow solution of complex **7** in benzene was added KO^{t}Bu (3 eq.) and stirred at room temperature for 2 h under Ar atmosphere. The resulting red solution was then filtered and evaporated under vacuum to afford complex **8**.

^{1}H NMR (400 MHz, 297 K, C$_6$D$_6$) δ 3.14-3.08 (m, 4H, NCH_2), 1.69-1.61 (m, 8H, PCH_2CH$_3$), 1.56-1.51 (m, 4H, NCH$_2$CH_2P), 1.04-0.96 (m, 12H, PCH$_2$CH_3); ^{31}P{^{1}H} NMR (162 MHz, 297 K, C$_6$D$_6$) δ 90.6. The spectroscopic data consistent with literature.[2]

S3 –Activation Study.

Base activation of complexes 2 and 3:

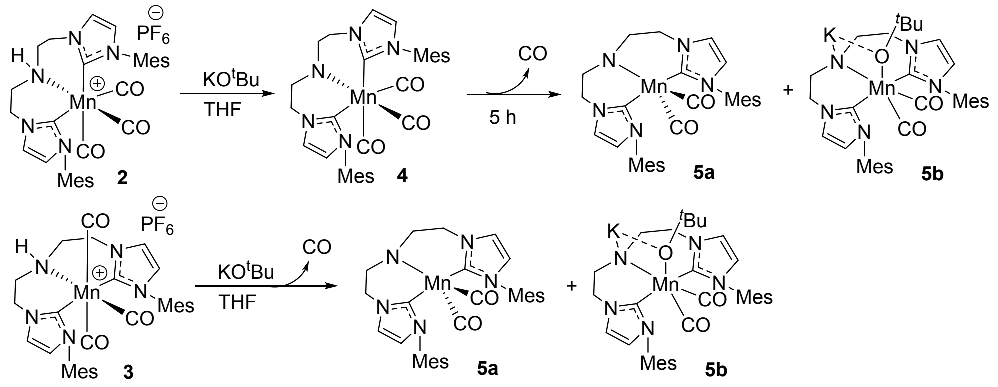

Treating complex **2** (4.0 mg, 1 equiv.) with KO^{t}Bu (0.9 mg, 1.5 equiv.) in THF (0.6 mL) immediately produced **4** as yellow solution as follows from IR measurements. The solution later turned blue, and species **4** was meanwhile observed to be fully converted to the mixture of **5a** and **5b** within 5 h. It was noted that the reaction mixture need to be kept dark, as **5b** was sensitive to light and would decompose with the upon light exposure. The assignments of **5b** were done with support of DFT calculation and IR spectroscopy (Figure S56).

The activation of complex **3** was performed through same procedure, but led immediately to **5**.

4 (*in situ*): IR (solution in THF, 297 K): ῡ [cm^{-1}] 1987 (s, ῡ CO), 1896 (s, ῡ CO), 1865 (s, ῡ CO). **5a** (*in situ*): IR (solution in THF, 297 K): ῡ [cm^{-1}] 1886 (s, ῡ CO), 1811 (s, ῡ CO). **5b** (*in situ*): IR (solution in THF, 297 K): ῡ [cm^{-1}] 1858 (s, ῡ CO), 1771 (s, ῡ CO).

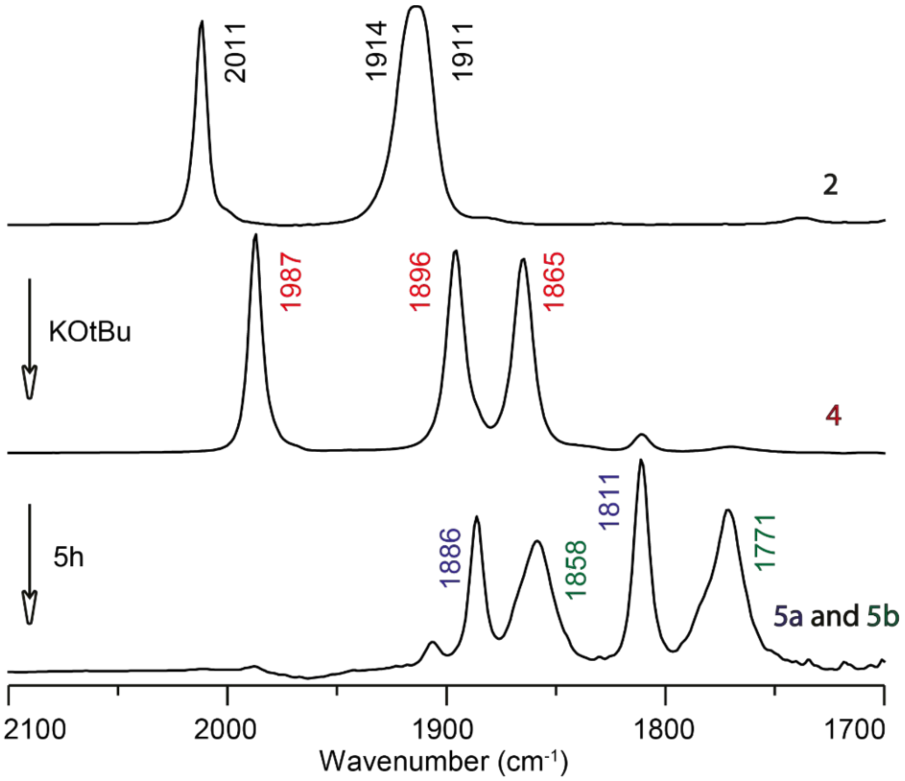

Figure S21. IR spectrum for *in situ* activation of **2** with KO^{t}Bu in THF.

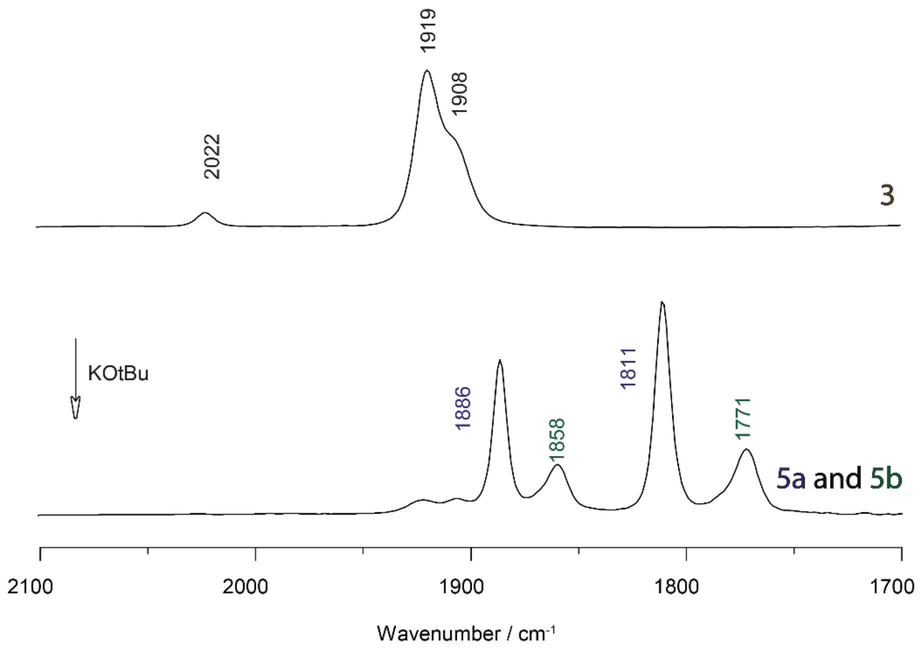

Figure S22. IR spectrum for *in situ* activation of **3** with KO^{t}Bu in THF.

H_2 promoted conversion of 5b to 5a:

The mixture of **5a** and **5b** obtained from complex **2** (Figure S21) was pressurized with 5 bar H_2 and dwelled at 25°C for 4 h. The solution was monitored by IR at 30, 150, 180 and 210 min.

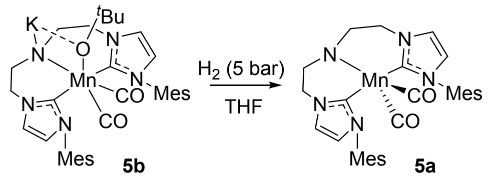

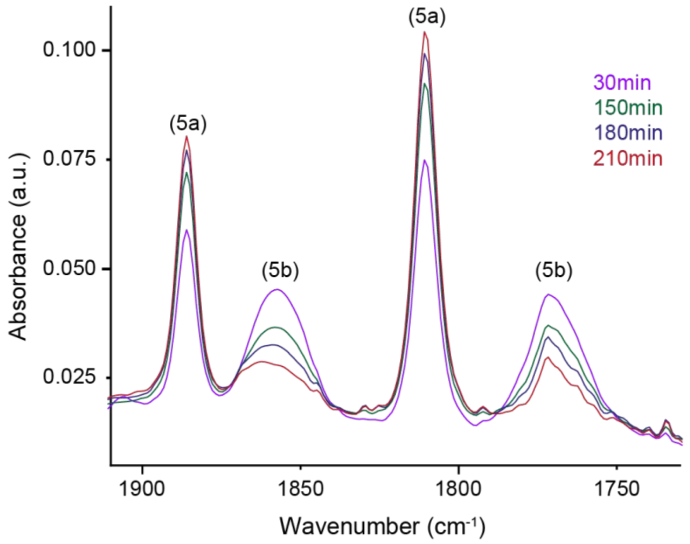

Figure S23. Evolution of IR spectrum of **5** mixture under H_2 for 30 min, 150 min, 180 min, and 210 min reaction times. Note the decrease of IR bands of complex **5b** along with the increase of bands of complex **5a** indicating conversion of **5b** to **5a**.

***In situ*-generation of Mn-alkoxide complex 6:**

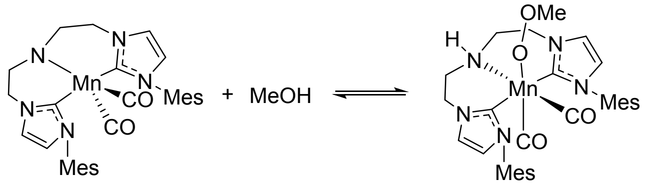

5a **6-OMe**

Upon treatment of complex **5a** (5.5 mg, 0.01 mmol) with MeOH (4.05 µL, 0.1 mmol) in THF-d_8 (0.6 mL) the solution immediately turned to green from blue, partially producing complex **6-OMe**. Since the equilibrium was exothermic (see S9), the samples were cooled to -40 °C and hold for 10 min. As NMR showed, complex **5a** was nearly fully converted to **6-OMe** featured as an yellow solution. We then run the full NMR characterization at this temperature.

Corresponding IR analysis was performed through the same procedure with 100 eq. MeOH in protic THF.

6-OMe (in situ): ^{1}H NMR (400 MHz, THF-d_8, 233K) δ 7.25 (s, 2H), 6.81 – 6.76 (m, 6H), 4.48 – 4.32 (m, 2H), 4.32 – 4.27 (m, 2H), 3.78 (s, 1H), 3.26– 3.17 (m, 2H), 2.93 (s, 3H), 2.76– 2.68 (m, 2H), 2.22 (m, 12H), 1.88 (s, 6H); ^{13}C NMR (101 MHz, THF-d_8, 233K) δ 232.9, 230.2, 207.8, 139.6, 138.7, 138.5, 138.3, 124.2, 123.8, 58.6, 53.0, 50.8, 49.4, 22.2, 19.8, 19.7; IR (solution in THF, 297 K): ῡ [cm^{-1}] 1886 (s, ῡ CO), 1811 (s, ῡ CO).

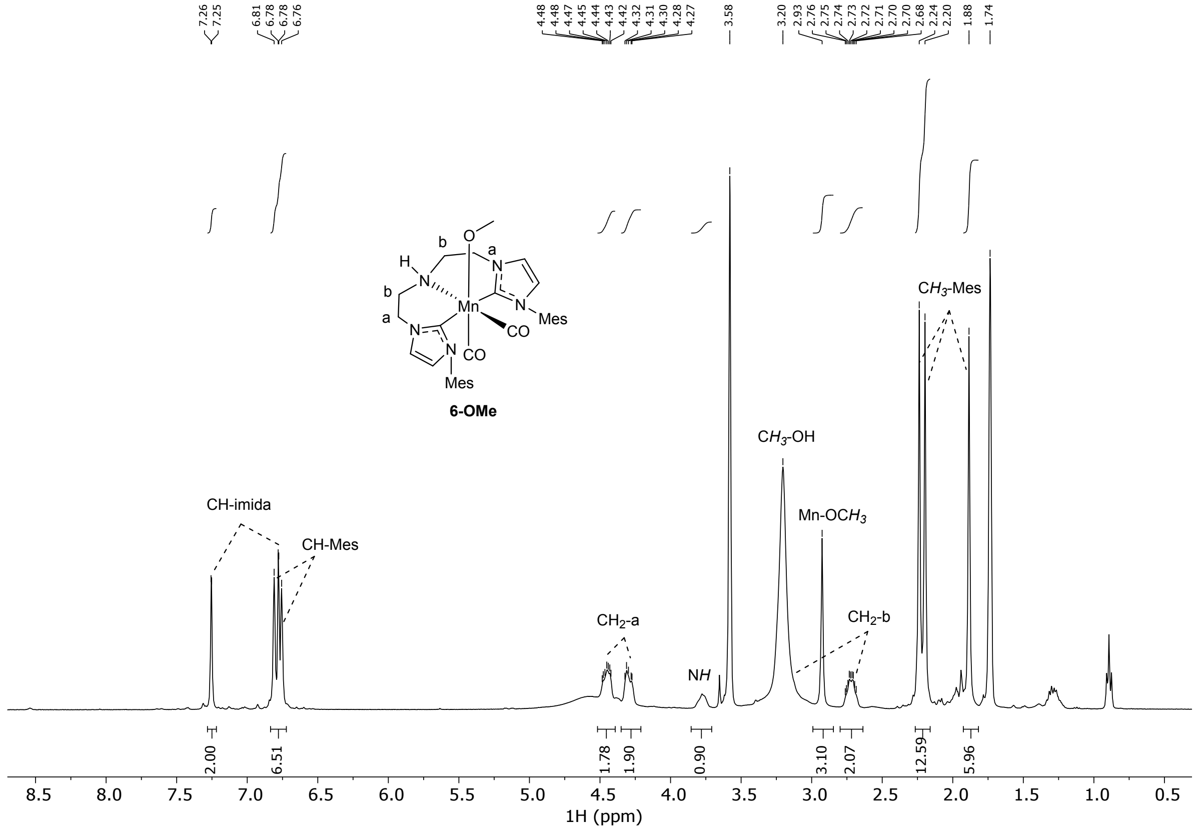

Figure S24. ^{1}H-NMR spectrum of complex **6-OMe** (*in-situ*) in THF-d$_8$ (400 MHz, 233K).

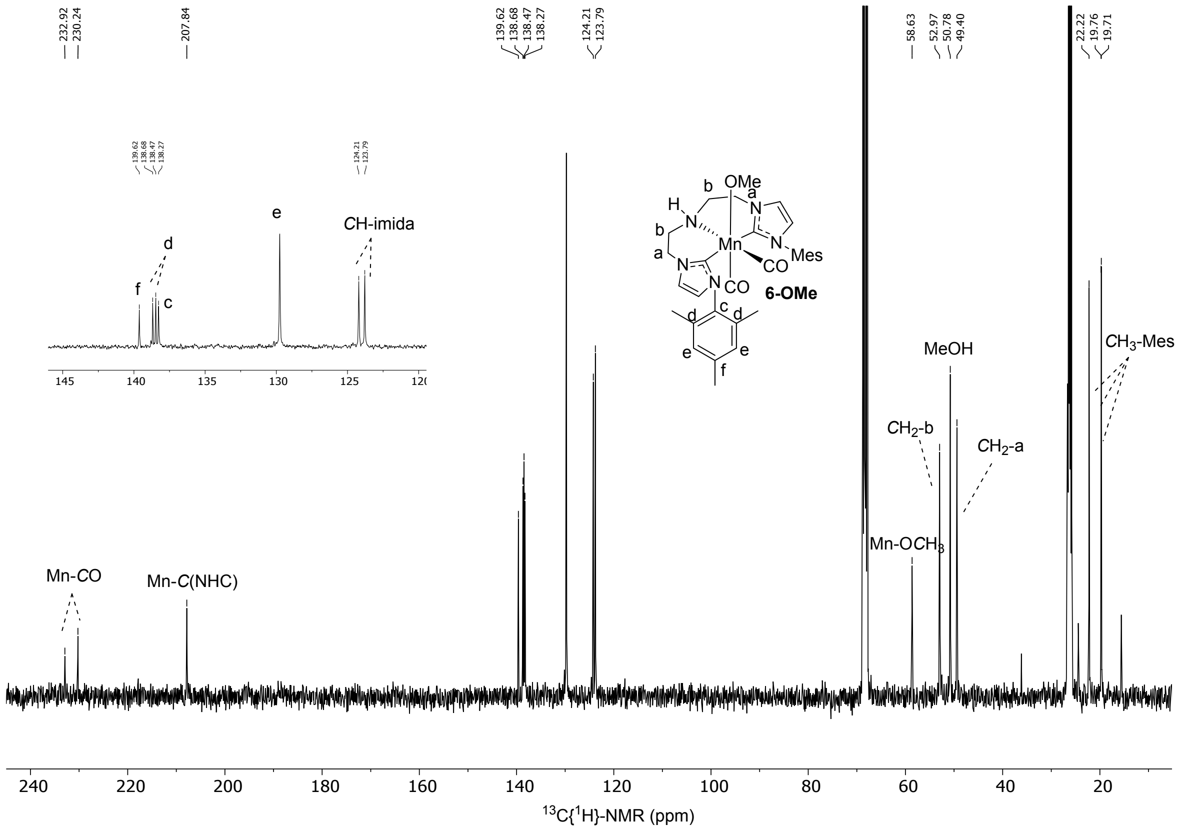

Figure S25. ^{13}C {^{1}H}NMR spectrum of complex **6-OMe** (*in-situ*) in THF-d$_8$ (400 MHz, 233K).

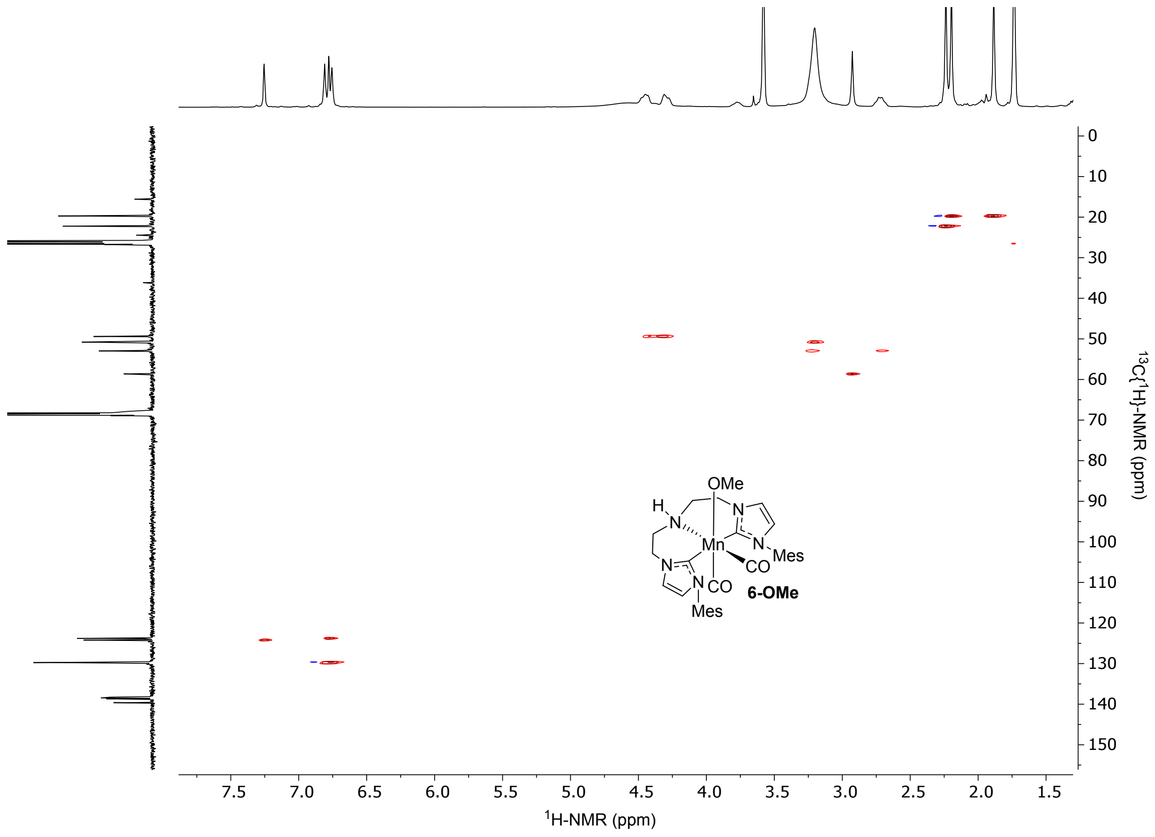

Figure S26. HMQC spectrum of complex **6-OMe** (*in-situ*) in THF-d$_8$ (400 MHz, 233K).

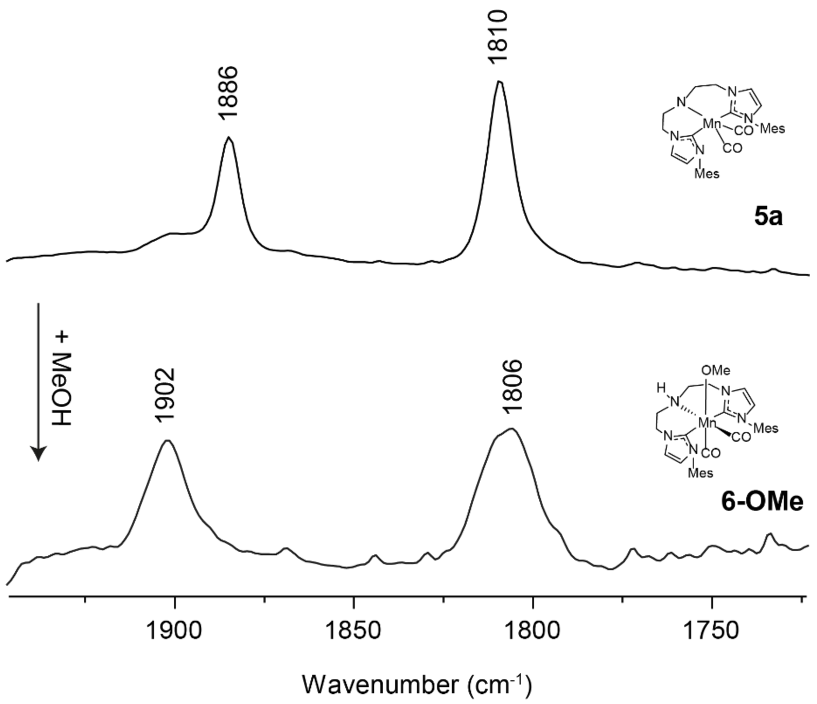

Figure S27. IR spectrum for *in situ* formation of **6-OMe** in THF.

Base effect on the equilibrium of 5a-6:

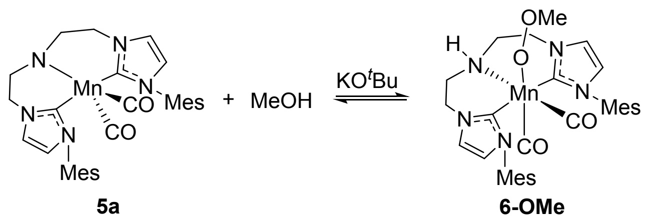

A NMR study was carried to confirm that excessive amount of base used in UV-vis study (see section S9) do not cause chemical change to complex **6**. To the THF-d_8 (0.6 mL) solution of complex **5a** (5.5 mg, 0.01 mmol) was added MeOH (12.2 µL, 0.3 mmol), and KO^{t}Bu (5.6 mg, 0.05 mmol). The resulting mixture was then characterized by ^{1}H NMR at -40 °C. The results (Figures S28 - 30) confirm the formation of Mn-alkoxide species and exclude further transformation caused by excessive KO^{t}Bu. In other words, the acquired thermodynamic parameters in Figure 6 of manuscript correspond to **5a-6** equilibrium.

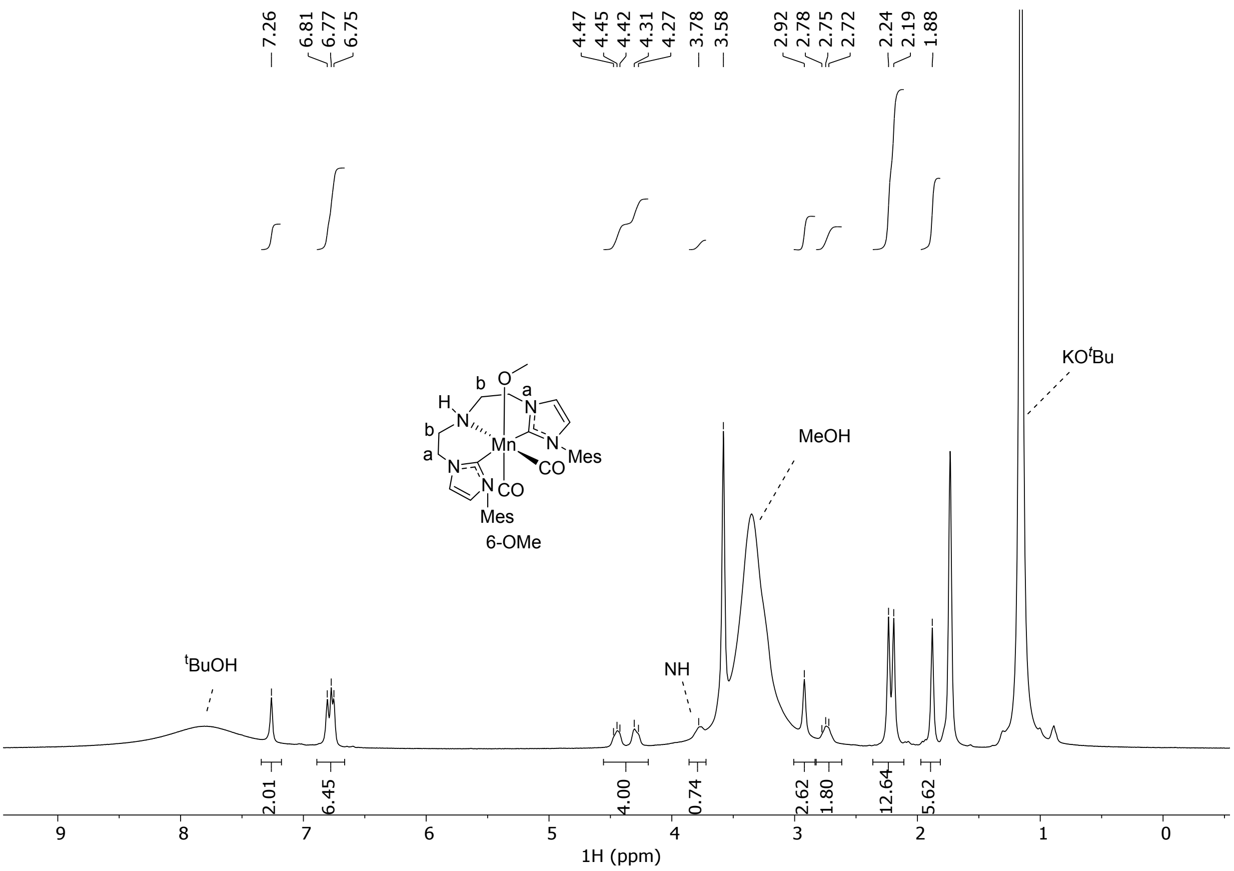

Figure S28. ^{1}H-NMR spectrum for the mixture of 5a, MeOH and KO^{t}Bu in THF-d_8 at -40 °C.

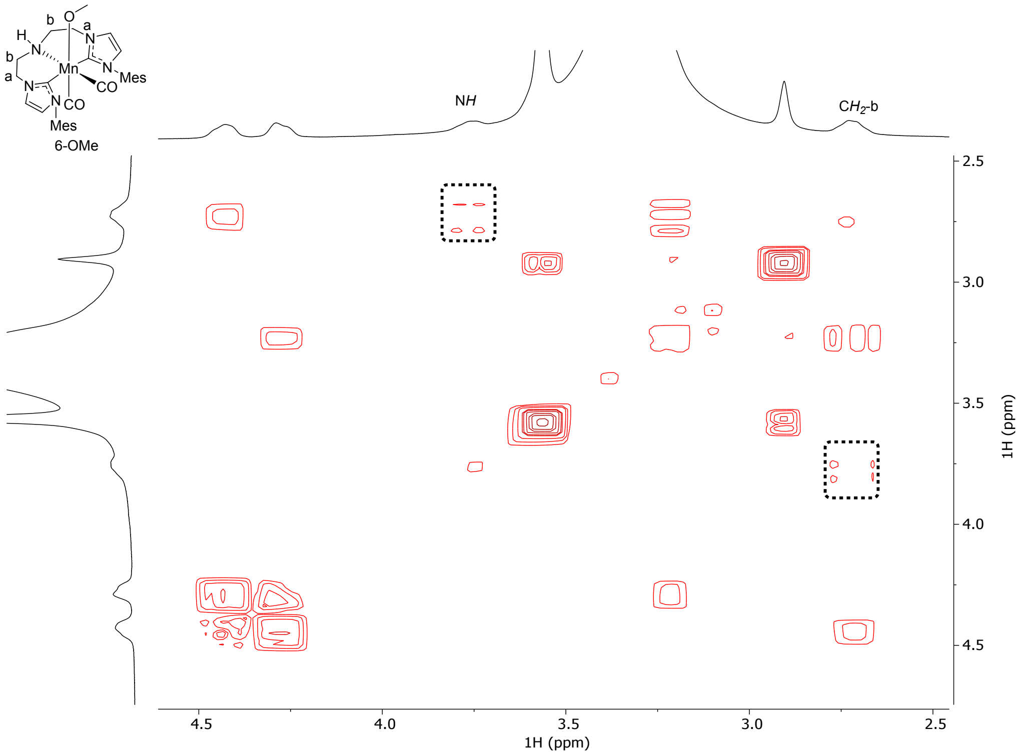

Figure S29. gCOSY spectrum for the mixture of 5a, MeOH and KO^{t}Bu in THF-d$_8$ at -40 °C. The cross-peaks for NH and CH$_2$-b are marked with dashed rectangle, confirming the presence of N-H group from complex **6** albeit with excessive base.

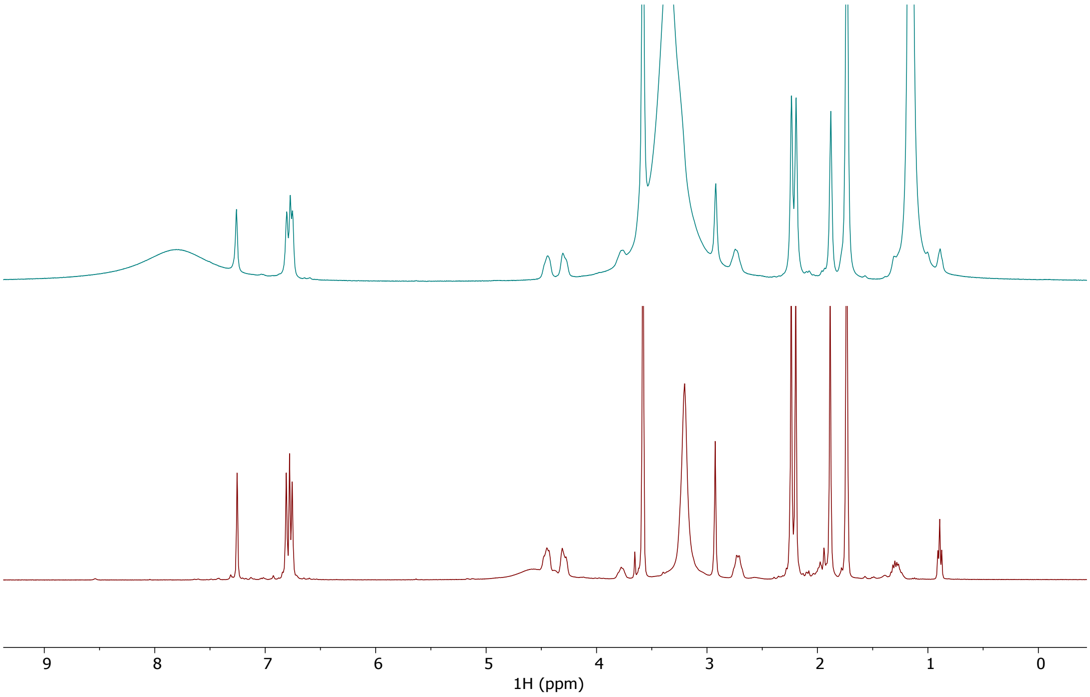

Figure 30. The stack ^{1}H NMR spectrums of the mixture of **5a** and MeOH in the presence (up) and absence (down, see Figure S24) KO^{t}Bu at -40 °C.

S4 – Catalytic Hydrogenation Details.

General procedure:

All the liquid substrates and internal standard regents (dodecane and 1-methyl naphthalene) were passed through a plug of neutral alumina, degassed and stored over molecular sieves in the glove box. Mn catalysts, KO^{t}Bu, substrates and solvents were handled in the glovebox. For initial experiments and optimisation, products were analyzed on an Agilent 7890 gas chromatograph equipped with an FID detector. Method details: Restek-Stabilwax column (30 m, 0.32 mm ID, 0.25 μm film thickness) and temperature profile from 50 °C (hold 1 min) up to 230 °C with ramp of 20 °C/min . Mass balances were verified to be within 90 % – 110 % for all experiments. Products were identified using retention times and peak areas from analytically pure reference samples.

Conditions screening with Mn catalyst 2 or 3:

Into a 4 mL brown glass vial was added Mn complex **2** or **3** (0.9, 1.8 mg for 0.1, 0.2 mol% respectively), KOtBu (2 mg), and solvent (0.2 mL). The solution was stirred in glove box at room temperature for 10 mins followed by the addition of ethyl hexanoate (207 μL, 1.25 mmol), KO^{t}Bu (12 mg, in total 10 mol%), solvent (0.3 mL), and dodecane (56.8 μL, 0.25 mmol). The vial was then transferred into a stainless steel autoclave and connected to gas line. The system was purged with N_2 (3×8 bar) and H_2 (1×30 bar), pressurized with H_2 to specified pressure, and heated to specified temperature. After the reaction, resulting mixture was quenched with H_2O (50 μL) and then GC samples were prepared by dilution of the reaction mixture in THF (20 μL into 1 mL THF).

Catalytic hydrogenation of esters with Mn catalyst 2:

Inside glovebox, solution of **2** (1.8 mg, 0.2 mol%) was prepared in 0.2 mL THF and activated with 2 mg KO^{t}Bu for 10 min. Substrate (1.25 mmol), KO^{t}Bu (12 mg, in total 10 mol%), solvent (0.3 mL), and 1-methyl naphthalene (89.0 μL, 0.627 mmol) were then added into the vial and transferred into a stainless steel autoclave. 10 μL reaction mixture was taken and dissolved in $CDCl_3$ or $(CD_3)_2SO$ as the reference for yield analysis. The system was purged with N_2 (3×8 bar) and H_2 (1×30 bar), pressurized with H_2 to specified pressure, and heated to specified temperature. After the reaction, resulting mixture was quenched with H_2O (50 μL) and then NMR and GC-MS samples were prepared by dilution of the reaction mixture in $CDCl_3$ or $(CD_3)_2SO$ (20 μL into 0.5 mL deuterium solvent) and THF (20 μL into 1 mL THF), respectively. The products were identified by GC-MS, and then quantified by NMR. GC-MS measurements were performed on an Agilent 7890B gas chromatograph equipped with FID and MS detectors. Method details: Agilent HP-5 column (30 m, 0.25 mm ID, 0.25 μm film thickness) and temperature profile from 60 °C (hold 2 min) up to 300 °C with ramp of 20 °C/min and hold at 300 °C for 5 minutes.

Catalytic hydrogenation of esters with Mn PNP catalyst 7:

Into a 4 mL brown glass vial was added Mn complex **7** (2.9 mg), KO^{t}Bu (2.8, 7 and 14 mg for 2, 5 and 10 mol% respectively), and THF (0.2 mL). The solution was stirred in glove box at room temperature for 10 mins followed by the addition of ethyl benzoate (178.6 μL, 1.25 mmol), KO^{t}Bu (12 mg, in total 10 mol%), THF (0.3 mL), and dodecane (56.8 μL, 0.25 mmol). The vial was then transferred into a stainless steel autoclave and connected to gas line. The system was purged with N_2 (3×8 bar) and H_2 (1×30 bar), pressurized with H_2 to 50 bar, and heated to 90 °C. After the reaction, resulting mixture was quenched with H_2O (50 μL) and then GC samples were prepared by dilution of the reaction mixture in THF (20 μL into 1 mL THF).

Replications studies:

To confirm the consistency of catalytic data for high temperature hydrogenation (100 and 110 °C) of ethyl hexanoate with different base loading were replicated. The results are shown below:

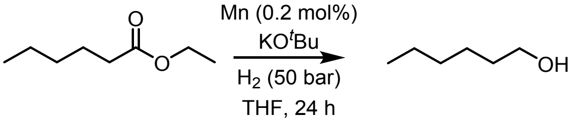

Replication	entries	T (°C)	KO^{t}Bu (mol%)	Conv. (%)	Yield (%)
Group 1	1	100	10	93	76
	2	100	20	96	96
	3	110	10	92	76
	4	110	20	93	88
Group 2	5	100	10	93	73
	6	100	20	96	96
	7	110	10	93	72
	8	110	20	93	80

Conditions: ethyl hexanoate (1.25 mmol), Mn catalyst **2**, KO^{t}Bu, THF (0.5 mL), P = 50 bar H_2, t = 24 h. Conversion and yield determined by GC analysis with dodecane as internal standard.

S5 – Optimization of Reaction Conditions.

Table S1. Condition screening for ethyl hexanoate hydrogenation with **Mn** catalysts.[a]

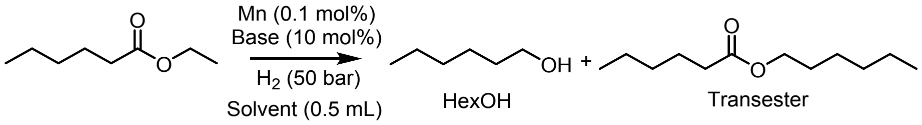

Entry	T/°C	Base	Cat.	solvent	t/h	HexOH/%	Transester/%	Yield/%
1[b]	70	KO^{t}Bu	**2**	THF	14	4	9	13
2[b]	70	KO^{t}Bu	**3**	THF	14	4	10	14
3	70	KO^{t}Bu	**2**	THF	14	14	18	32
4	70	KOEt	**2**	THF	14	12	16	28
5	70	NaH	**2**	THF	14	7	15	22
6	70	KHMDS	**2**	THF	14	4	13	17
7	70	KO^{t}Bu	**2**	dioxane	24	22	20	42
8	70	KO^{t}Bu	**2**	Toluene	24	12	19	31
9	70	KO^{t}Bu	**2**	DMSO	24	10	8	18
10	70	KO^{t}Bu	**2**	THF	24	29	20	49
11	60	KO^{t}Bu	**2**	THF	24	18	20	38
12	80	KO^{t}Bu	**2**	THF	24	53	16	68

[a] Reactions were conducted with ethyl hexanoate (1.25 mmol), Mn catalyst (0.1 mol%), KO^{t}Bu in solvent (0.5 mL) under 50 bar H_2. Yield of each component was determined by GC with dodecane as internal standard. [b] 2.5 mL solvent was used instead of 0.5 mL.

S6 – Substrate scope

Table S2. Substrate scope for esters hydrogenations with 2.[a]

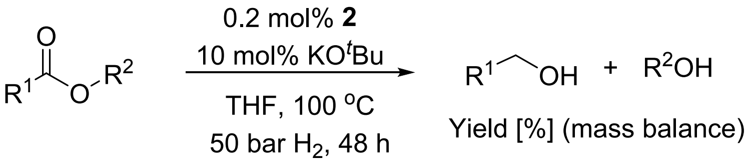

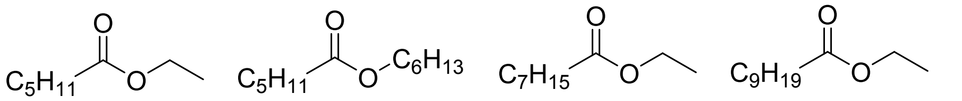

A1, 90% (1.01) **A2**, 99% (0.93) **A3**, 80% (0.99) **A4**, >99% (0.99)

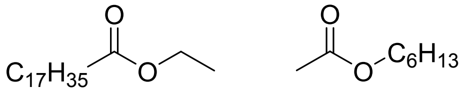

A5, 96% (1.13) **A6**, >99% (0.90)

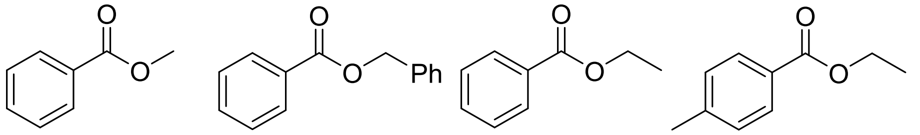

A7, 61% (1.08) **A8**, 66% (1.08) **A9**, 86% (0.99) **A10**, 70% (1.08)

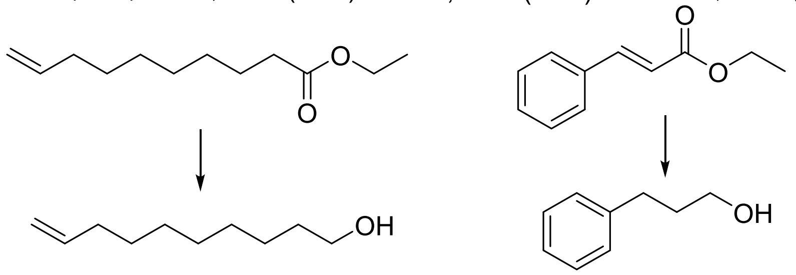

A11, >99% (1.07) **A12**, 91% (1.01)

[a] Reactions were conducted with ethyl hexanoate (1.25 mmol), Mn catalyst (0.1 mol%), KO^{t}Bu in solvent (0.5 mL) under 50 bar H_2. Yield of each component was determined by GC-MS and ^{1}HNMR with 1-methyl naphthalene as internal standard. The mass balance was given in the brackets.

S7 – *Operando* spectroscopy studies.

Device:

The high pressure operando IR measurements were done using a 50 NmL stainless steel jacketed autoclave (Parr 4597) equipped with mechanical stirrer and a pressure compensation system (Parr A2280HC). The reactor was thermostated with an external circulation thermostat. To initiate the reaction under target temperature and hydrogen pressure the catalyst solution was injected using a manual home-built liquid injector. To collect kinetic and spectroscopy data under the reaction conditions a CyberHydra™ (https://www.cyberhydra.nl/) integrated solution was used. The system included a closed-loop thermostated circuit allowing to fluidically couple the reactor with an IR transmittance flow-through cell and a sampling system and to circulate the reaction mixture.[Sampling system: doi.org/10.1016/j.inv.2021.100002, CyberHydra] The IR cell (Harrick Scientific, DLC-S13 or CyberHydra IR cell 1) was equipped with CaF_2 windows as well as PTFE wedged spacers, which provided a 0.15 mm optical path length. The spectra were collected with a Bruker Alpha II spectrometer with a spectral resolution of 2 cm^{-1}.

Experimental procedures: Inside glovebox, a catalyst solution of **2** (8.7 mg, 0.125 mmol) was prepared in 0.7 mL THF and activated with 10 mg KO^{t}Bu for 10 min. The resulting solution was then loaded in a 1mL syringe. A 20 mL syringe was loaded with ester substrate (12.5 mmol) and dodecane (568 μL, 2.5 mmol) in 7.5 mL THF (total volume 10 mL). Under N_2 flow, the substrate syringe was first injected into high pressure stainless steel reactor in which a glass beaker was inserted in advance. In an injection port the dissolved catalyst mixture was then placed and activated for 2 h under 40 bar (H_2). The reaction was started by injecting the catalyst mixture at 120 °C with stirring at 500 r.p.m, and the FTIR measurements and the sampling procedure were started.
FTIR spectra were recorded from 4000 to 400 cm^{-1} with resolution of 2 cm^{-1}. Per spectrum were collected with 16 scans. Intervals between two measurements were 5 min (for the first 12 h) and after that 10 min.
FTIR data processing: The raw data were processed by baseline subtraction within metal-carbonyl wavenumber region (2100–1700 cm^{-1}) in OriginPro 2019. 10 anchor points snapped to the spectrum were selected (1763.7, 1767.7, 1781.8, 1789.8, 1829.9, 1835.5, 1839.9, 1867.3, 1916.6, 2020.1, 2052.2 cm^{-1}, can be modified slightly depending on the real spectrum) to fit the base line via 2nd derivative mode with 0.05 threshold. Beta spline was used as the interpolation method. The obtained base line-subtracted data were then used directly for spectrum plotting or integral analysis for all the Mn species. In the processed IR spectrum, one of CO vibration bands of complex **5a** (1810 cm^{-1}) overlapped with that of **6** (1806 cm^{-1}), thus these two peaks should be deconvoluted for integration analysis. Therefore, the integral of the band of **5a** was directly calculated based on that of the second band (1886 cm^{-1}), as the proportion between these two bands could be assumed constant. We got the average coefficient β_{5a}

$= \frac{Intergal_{1810}}{Intergal_{1886}}$ as 1.4835 based on the IR results depicted in Figures S19, S21, S22, S23, S27. Thus the integral of 1806 cm^{-1} band of **6** was obtained by subtracting the total integral of the peaks in 1825–1775 cm^{-1} by that of 1810 cm^{-1} band of **5a**. Similarly, one band of complex **5b** (1771 cm^{-1}) was fully overlapped with the carbonyl of ester substrates with high concentration. The coefficient β_{5b} for this band was thus obtained as 1.334 through the same method. The total integral of all Mn species observed in the spectrum were shown to be nearly constant (Figures S32, 34, 36, 38, 40). Therefore we conclude the extinction coefficient for all the Mn compounds is identical and no deactivation through other pathway (e.g. decarbonylation) occurs.

Screening *operando* data depicted in Figure 4 of the manuscript for ethyl hexanoate substrate:

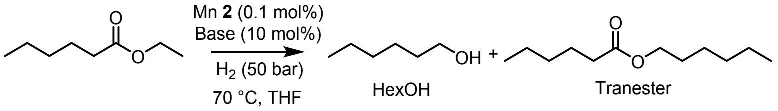

Conditions: Reactions were conducted with ethyl hexanoate (1.25 M), Mn catalyst **2** (0.1 mol%), KO^{t}Bu (10 mol%) in THF (8.2 mL, total volume 10 mL) at 70°C under 50 bar H_2.

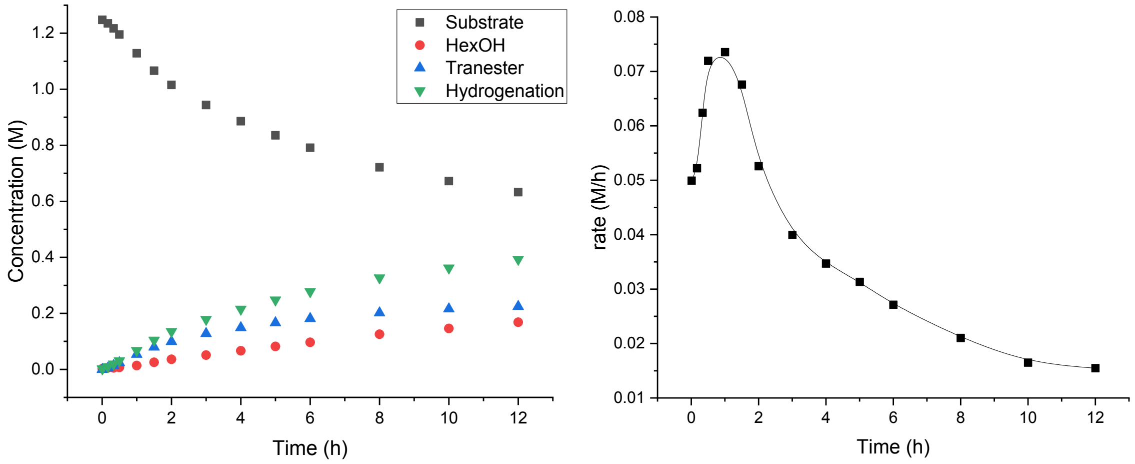

Figure S31. Concentration profile (left) and catalytic rate profile (right) for preliminary *operando* study of hydrogenation of ethyl hexanoate obtained from GC analysis.

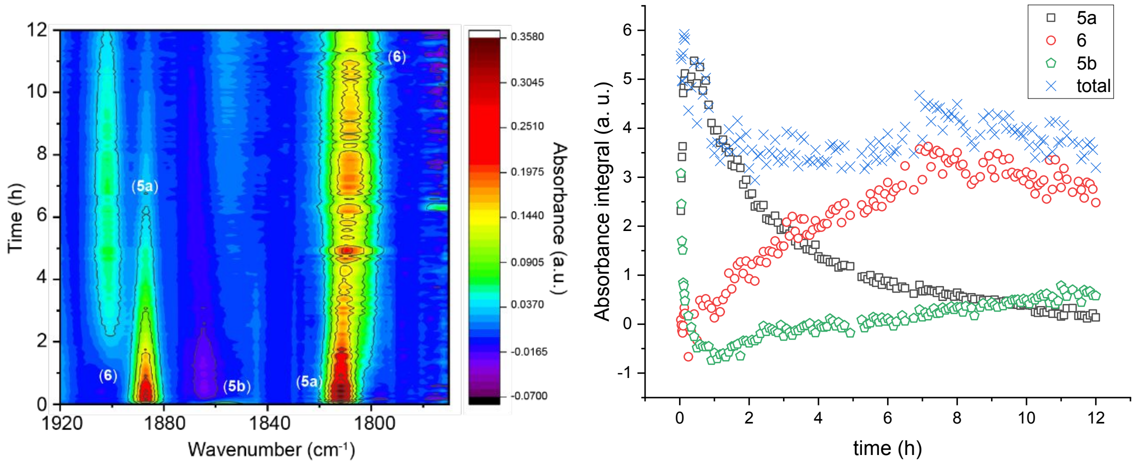

Figure S32. Real-time IR spectrum (left) and kinetic IR profile (right) for screening *operando* study on hydrogenation of ethyl hexanoate.

***Operando* data for hydrogenation of hexyl hexanoate under standard conditions**:

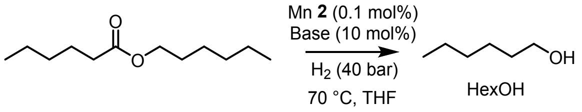

Conditions: Reactions were conducted with hexyl hexanoate (1.25 M), Mn catalyst **2** (0.1 mol%), KO^{t}Bu (10 mol%) in THF (8.2 mL, total volume 10 mL) at 70°C under 40 bar H_2.

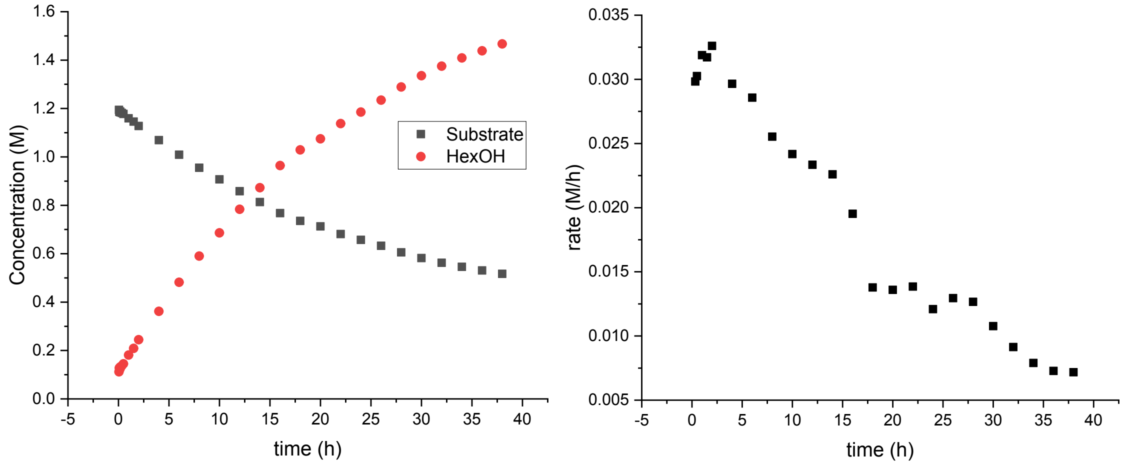

Figure S33. Concentration profile (left) and catalytic rate profile (right) for *operando* study on hydrogenation of hexyl hexanoate under standard conditions obtained from GC analysis.

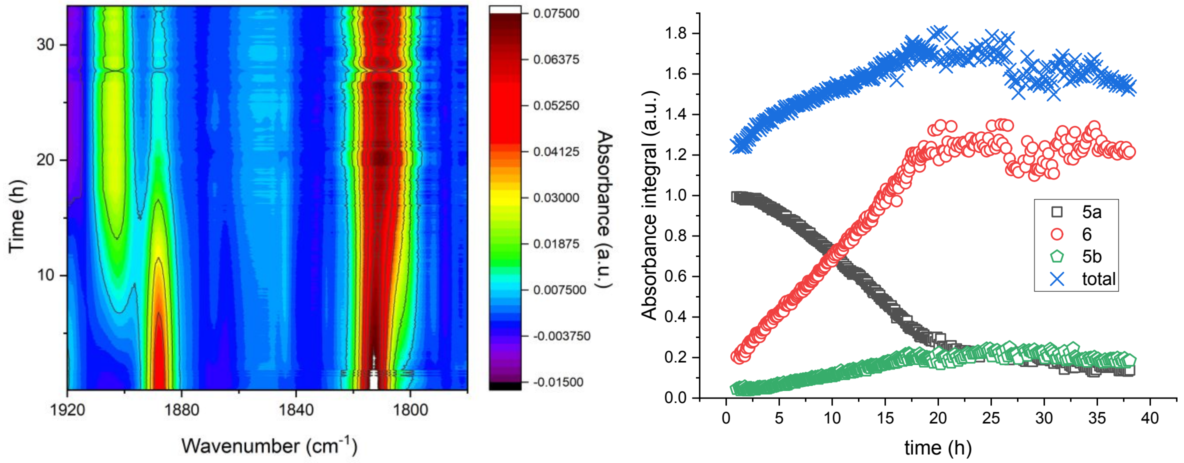

Figure S34. Real-time IR spectrum (left) and kinetic IR profile (right) for *operando* study on hydrogenation of hexyl hexanoate under standard conditions. Since the signal of species **5b** was negligible, an internal normalization was applied to **5a** and **6** (see manuscript, Figure 5D) so that the kinetic IR trends could be easily compared with those of other control studies.

Operando data for hydrogenation of hexyl hexanoate under hexanol addition conditions:

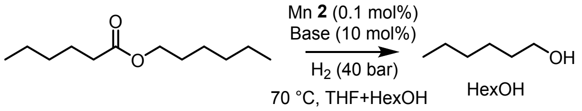

Conditions: Reactions were conducted with hexyl hexanoate (1.25 M), hexanol (1.25 M), Mn catalyst **2** (0.1 mol%), KO^{t}Bu (10 mol%) in THF (5.7 mL, total volume 10 mL) at 70°C under 40 bar H_2

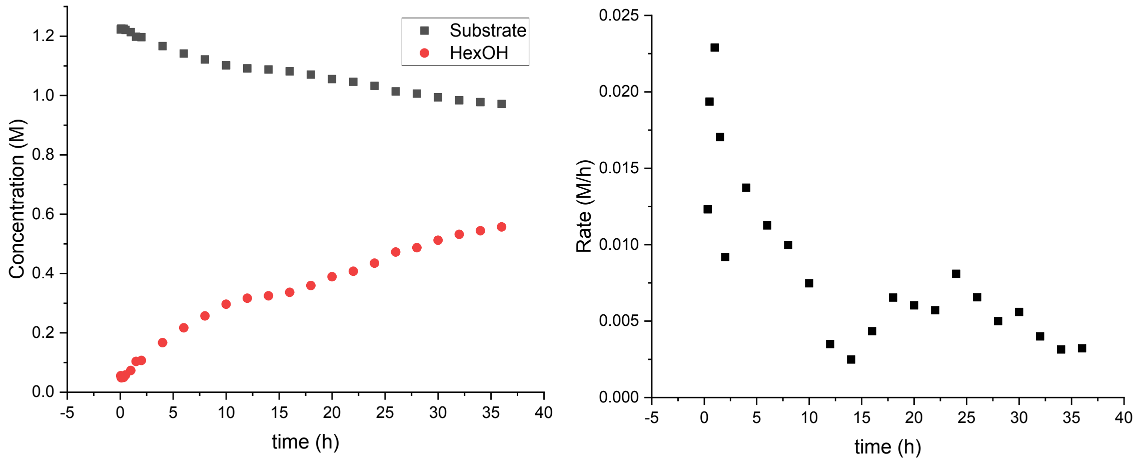

Figure S35. Concentration profile (left) and catalytic rate profile (right) for *operando* study on hydrogenation of hexyl hexanoate with hexanol addition obtained from GC analysis.

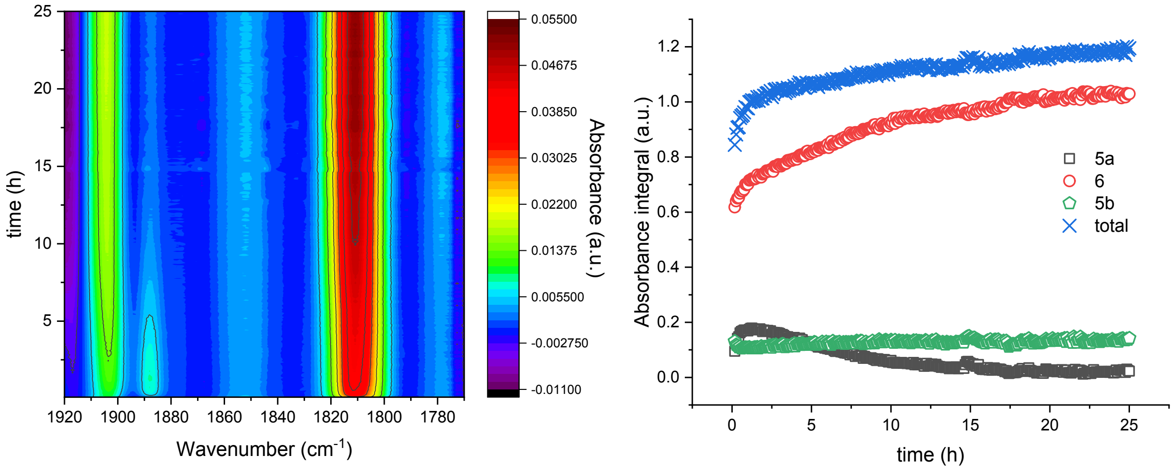

Figure S36. Real-time IR spectrum (left) and kinetic IR profile (right) for *operando* study on hydrogenation of hexyl hexanoate with hexanol addition. Since the signal of species **5b** was negligible, an internal normalization was applied to **5a** and **6** (see manuscript, Figure 5F) so that the kinetic IR trends could be easily compared with those of other control studies.

***Operando* data for hydrogenation of hexyl hexanoate under *tert*-butanol addition conditions**:

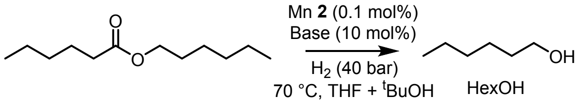

Conditions: Reactions were conducted with hexyl hexanoate (1.25 M), *tert*-butanol (1.25 M), Mn catalyst **2** (0.1 mol%), KO*t*Bu (10 mol%) in THF (5.7 mL, total volume 10 mL) at 70°C under 40 bar H_2.

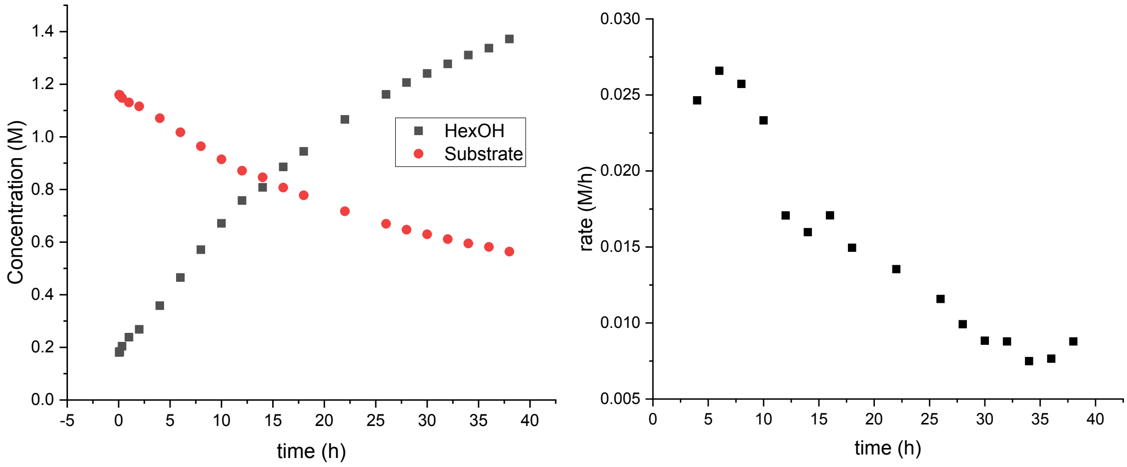

Figure S37. Concentration profile (left) and catalytic rate profile (right) for *operando* study on hydrogenation of hexyl hexanoate with *tert*-butanol addition obtained from GC analysis.

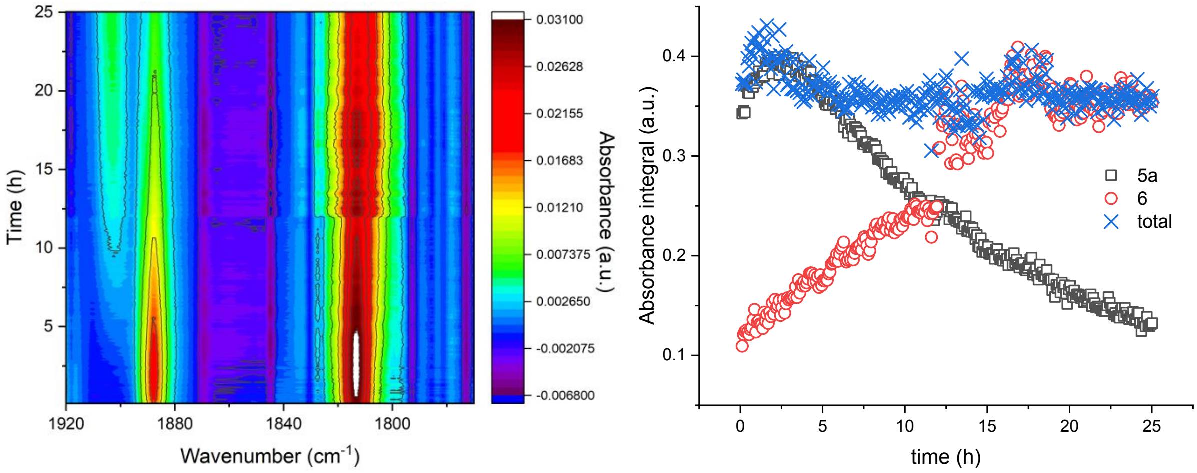

Figure S38. Real-time IR spectrum (left) and kinetic IR profile (right) for *operando* study on hydrogenation of hexyl hexanoate with *tert*-butanol addition. The species **5a** were not observed at all.

***Operando* data for hydrogenation of hexyl hexanoate under reduced base loading conditions**:

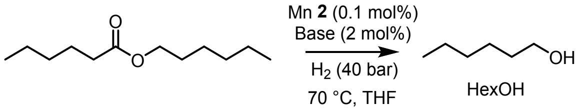

Conditions: Reactions were conducted with hexyl hexanoate (1.25 M), Mn catalyst **2** (0.1 mol%), KO^{t}Bu (2 mol%) in THF (5.7 mL, total volume 10 mL) at 70°C under 40 bar H_2.

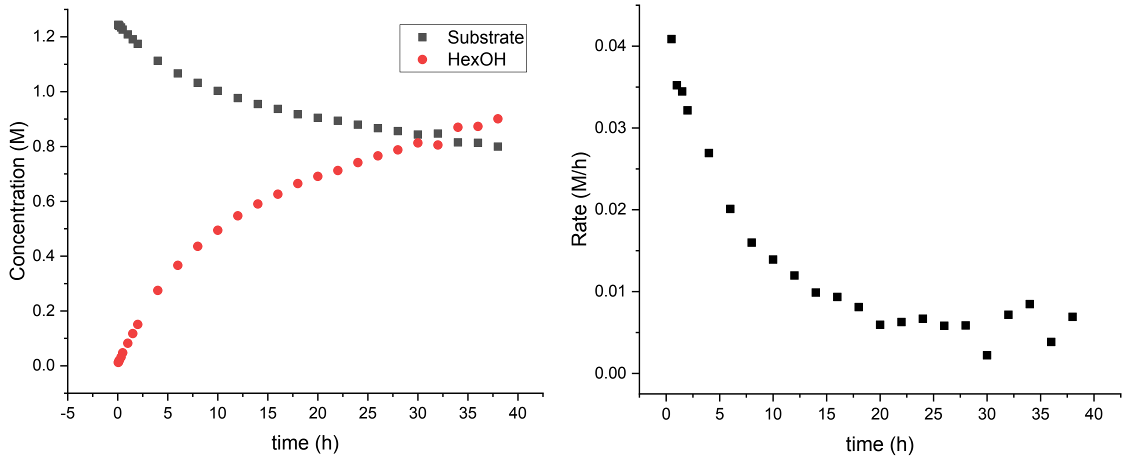

Figure S39. Concentration profile (left) and catalytic rate profile (right) for *operando* study on hydrogenation of hexyl hexanoate with low base loading obtained from GC analysis.

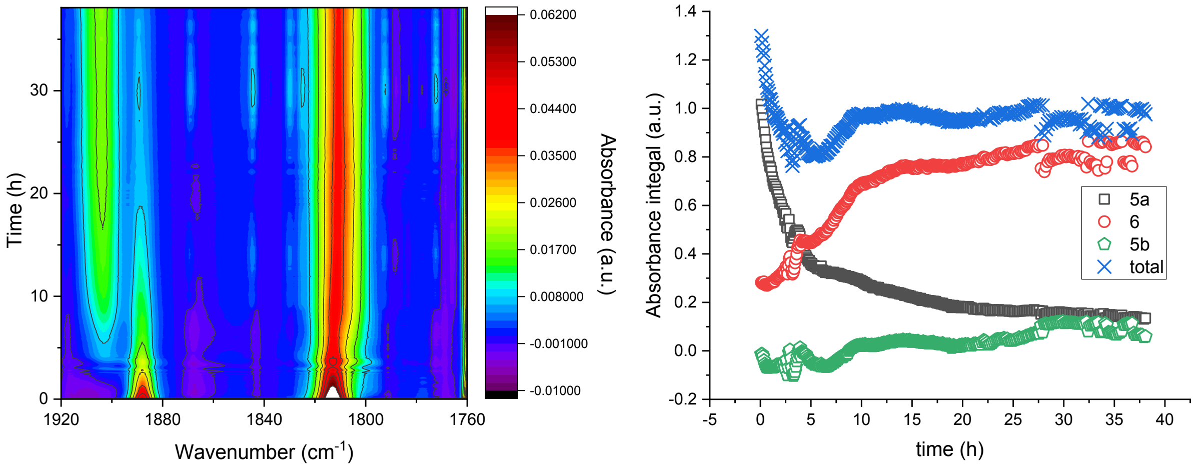

Figure S40. Real-time IR spectrum (left) and kinetic IR profile (right) for *operando* study on hydrogenation of hexyl hexanoate with low base loading. Since the signal of species **5b** was negligible, an internal normalization was applied to **5a** and **6** (see manuscript, Figure 5E) so that the kinetic IR trends could be easily compared with that of other control studies.

S8- UV-vis Studies

Analysis of the equilibrium of complex 5a with different alcohols

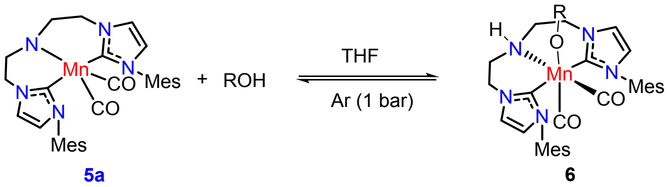

1.6 mL of 0.567 mM solution of **Mn-5a** (0.5 mg) in THF were added to two identical cuvettes inside glovebox. The first sample was pure complex solution as reference. To the second sample was added corresponding amount of alcohol. For base effect study, base was mixed with the stock solution before adding to cuvette. The cuvettes were capped with Teflon seals, removed from glovebox, and loaded into UV-vis spectrometer and measured through transmission channel. The samples were heated or cooled to specified temperature and hold for 5 min before spectra were collected. All the transmission data were converted to absorbance for further analysis. The concentration of Mn species was determined by the absorbance at 583 nm. The temperature dependence of K_{eq} was assessed via van't Hoff plots.

$$\text{At equilibrium:} \quad \Delta G° = \Delta H° - T\Delta S° = -RTln(K_{eq})$$

$$ln(K_{eq}) = \frac{-\Delta H°}{RT} + \frac{\Delta S°}{R}$$

Thus for the plots of $\ln(K_{eq})$–1/RT:

$$-\Delta H° = slope; \frac{\Delta S°}{R} = intercept.$$

Temperature dependence of the extinction coefficient of 5a:

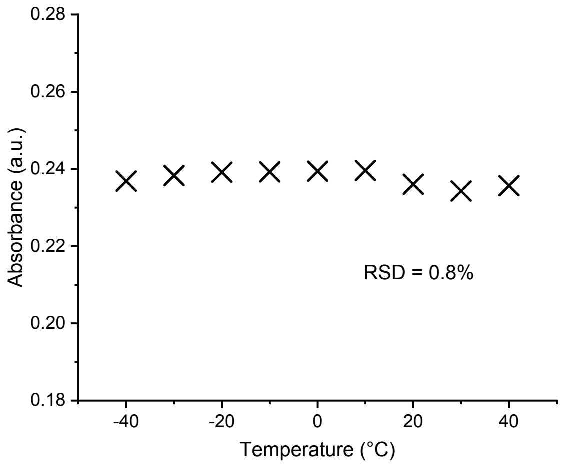

Figure S41. The UV-vis absorbance at 583 nm of the solution of **Mn-5a** (0.567mM) in THF at different temperatures. The relative standard deviation (RSD) of measured values is 0.8%. We concluded that the extinction coefficient of **5a** was temperature independent within the examined range. The following absorbance data used for equilibrium constant calculation thus do not require additional corrections.

Equilibrium in Mn-5a-methanol mixtures:

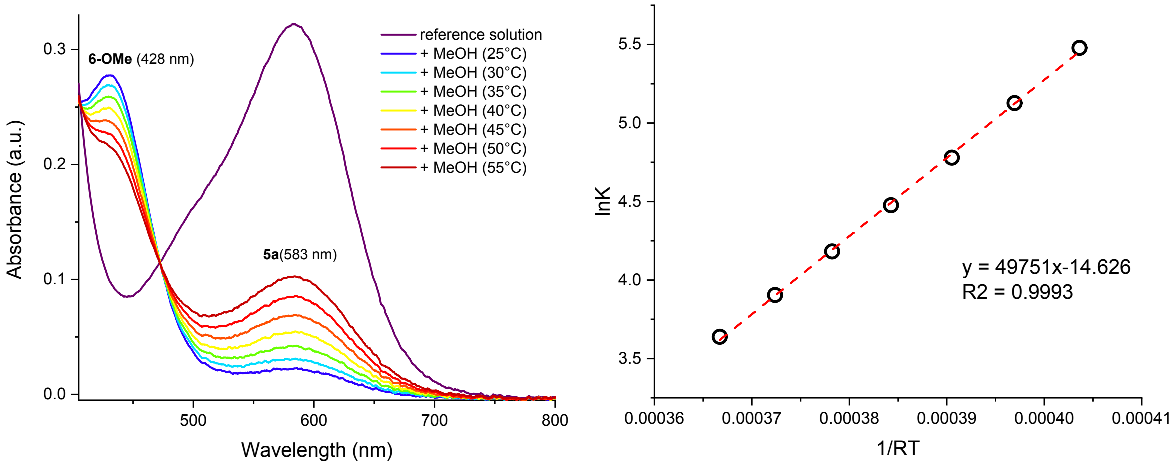

Figure S42. The UV-vis spectra and resulting Van't Hoff plots of the mixture of Mn-**5a** (0.567mM) with methanol (55.6 mM) at different temperatures. According to the plots, equilibrium parameters were calculated out as: $\Delta H° =$ -49.8 ± 0.6 kJ·mol^{-1}; $\Delta S° =$ -121.6 ± 1.9 J·mol^{-1}·K^{-1} $\Delta G° =$ -13.5 ± 0.8 kJ·mol^{-1} (298 K).

Table S3. Equilibrium constant output from UV-vis spectrum Mn-**5a** (0.567mM) with methanol (55.6 mM).

Entry	T (K)	1/RT (mol·J^{-1})	Absorbance (583nm, a.u.)	[**5a**](mM)	[**6-OMe**](mM)	K_{eq} (M^{-1})	ln (K_{eq})
1	298	0.0004036	0.02228	0.03924	0.52791	239.44567	5.47833
2	303	0.000397	0.03077	0.05419	0.51296	168.42276	5.12648
3	308	0.0003905	0.04187	0.07374	0.49341	119.01441	4.77924
4	313	0.0003843	0.05419	0.09544	0.47171	87.87900	4.47596
5	318	0.0003782	0.06875	0.12108	0.44607	65.47252	4.18163
6	323	0.0003724	0.08486	0.14945	0.41770	49.64422	3.90488
7	328	0.0003667	0.10248	0.18049	0.38667	38.03356	3.63847
8	Reference solution		0.32203				

Equilibrium in Mn-5a-hexanol mixtures:

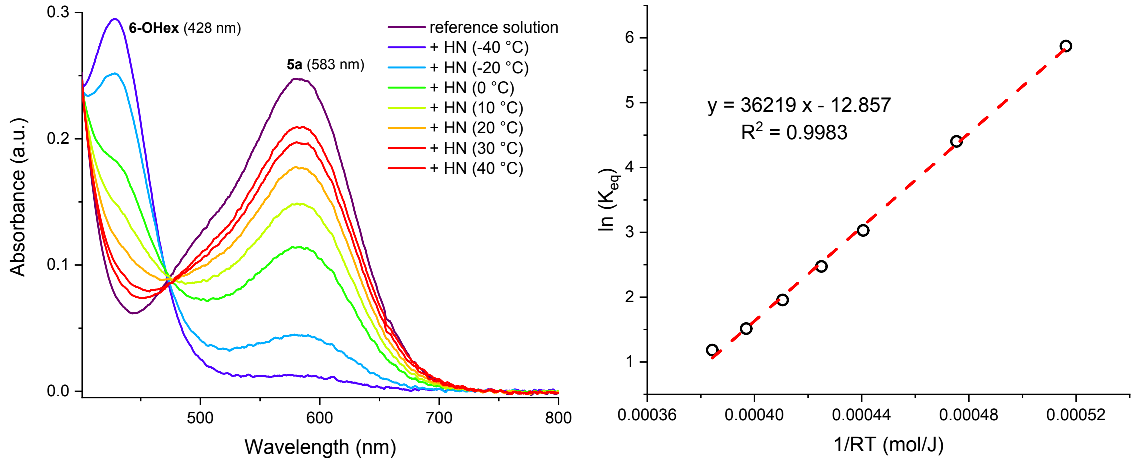

Figure S43. The UV-vis spectra and resulting Van't Hoff plots of the mixture of Mn-**5a** (0.567mM) with hexanol (55.6 mM) at different temperatures. According to the plots, equilibrium parameters were calculated out as: $\Delta H° =$ -36.2 ± 0.7 kJ·mol^{-1}; $\Delta S° =$ -106.9 ± 2.5 J·mol^{-1}·K^{-1}; $\Delta G° =$ -4.4 ± 1.0 kJ·mol^{-1} (298 K).

Table S4. Equilibrium constant output from UV-vis spectrum of the mixture of Mn-**5a** (0.567mM) with hexanol (55.6 mM).

Entry	T (K)	1/RT (mol·J^{-1})	Absorbance (583nm, a.u.)	[**5a**](mM)	[**6-OHex**](mM)	K_{eq} (M^{-1})	ln (K_{eq})
1	233	0.000516	0.01178	0.02704	0.54011	355.59233	5.87378
2	253	0.000475	0.04420	0.10145	0.46570	81.60575	4.40190
3	273	0.000441	0.11407	0.26183	0.30532	20.67242	3.02880
4	283	0.000425	0.14790	0.33948	0.22767	11.87266	2.47424
5	293	0.000411	0.17653	0.40519	0.16196	7.06779	1.95555
6	303	0.000397	0.19651	0.45105	0.11610	4.54764	1.51461
7	313	0.000384	0.20855	0.47869	0.08846	3.26348	1.18279
8	Reference solution		0.24709				

Equilibrium in Mn-5a-benzyl alcohol mixtures:

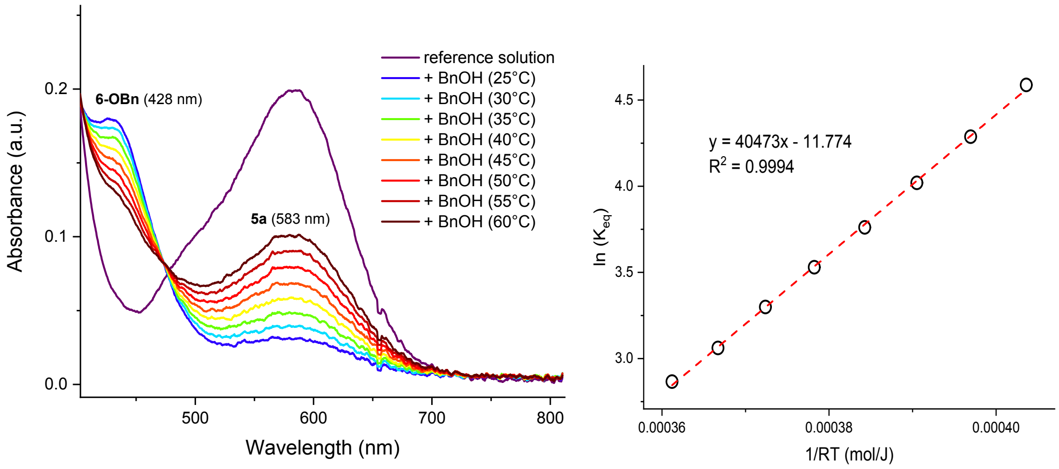

Figure S44. The UV-vis spectra and resulting Van't Hoff plots of the mixture of Mn-**5a** (0.567mM) with benzyl alcohol (55.6 mM) at different temperatures. According to the plots, equilibrium parameters were calculated out as: $\Delta H° =$ -40.5 ± 0.4 kJ·mol^{-1}; $\Delta S° =$ -97.9 ± 1.3 J·mol^{-1}·K^{-1}; $\Delta G° =$ -11.3 ± 0.6 kJ·mol^{-1} (298 K).

Table S5. Equilibrium constant output from UV-vis spectrum Mn-**5a** (0.567mM) with benzyl alcohol (55.6 mM).

Entry	T (K)	1/RT (mol·J^{-1})	Absorbance (583nm, a.u.)	[**5a**](mM)	[**6-OBn**](mM)	K_{eq} (M^{-1})	ln (K_{eq})
1	298	0.0004036	0.03049	0.08692	0.48023	98.24448	4.58746
2	303	0.0003970	0.03905	0.11133	0.45582	72.77905	4.28743
3	308	0.0003905	0.04813	0.13721	0.42994	55.66993	4.01944
4	313	0.0003843	0.05814	0.16575	0.40140	43.00450	3.76130
5	318	0.0003782	0.06808	0.19409	0.37306	34.11576	3.52976
6	323	0.0003724	0.07873	0.22445	0.34270	27.08533	3.29899
7	328	0.0003667	0.09023	0.25723	0.30992	21.35993	3.06152
8	333	0.0003612	0.09991	0.28483	0.28232	17.56413	2.86586
9	Reference solution		0.19894				

Base effects on the equilibrium in Mn-5a-hexanol mixtures:

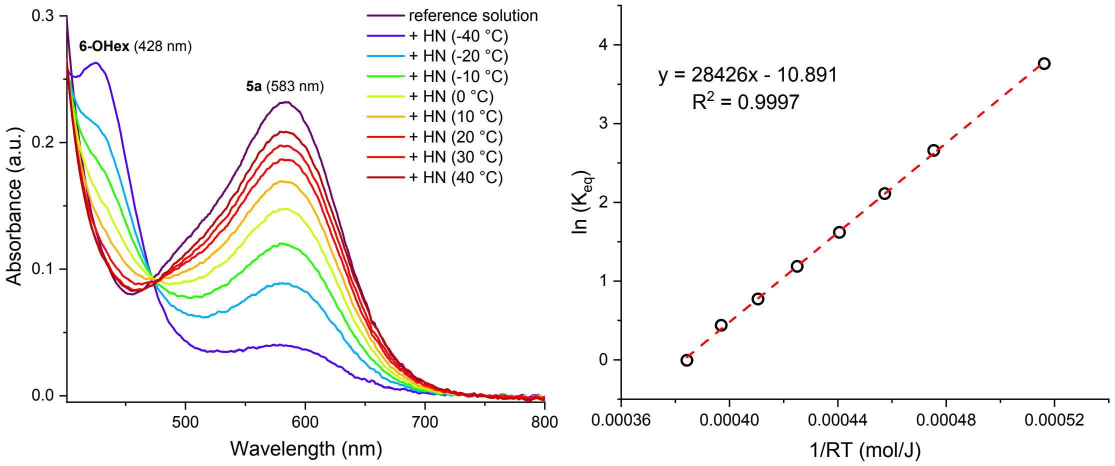

Figure S45. The UV-vis spectra and resulting Van't Hoff plots of the mixture of Mn-**5a** (0.567mM), hexanol (113.4 mM), and KO^{t}Bu (28.35 mM) in THF at different temperatures. According to the plots, equilibrium parameters were calculated out as: $\Delta H° =$ -28.4 ± 2.4 kJ·mol^{-1}; $\Delta S° =$ -90.5 ± 0.9 J·mol^{-1}·K^{-1}; $\Delta G° =$ -1.4 ± 0.4 kJ·mol^{-1} (298 K).

Table S6. Equilibrium constant output from UV-vis spectrum of the mixture of Mn-**5a** (0.567mM), hexanol (113.4 mM), and KO^{t}Bu (28.35 mM) in THF.

Entry	T (K)	1/RT (mol·J^{-1})	Absorbance (583nm, a.u.)	[**5a**](mM)	[**6-OHex**](mM)	K_{eq} (M^{-1})	ln (K_{eq})
1	233	0.0005162	0.03958	0.09683	0.47032	42.99758	3.76114
2	253	0.0004754	0.08867	0.21693	0.35022	14.27674	2.65863
3	263	0.0004573	0.11982	0.29314	0.27401	8.26058	2.11150
4	273	0.0004406	0.14751	0.36089	0.20627	5.04800	1.61899
5	283	0.000425	0.16907	0.41363	0.15352	3.27648	1.18677
6	293	0.0004105	0.18613	0.45537	0.11178	2.16623	0.77299
7	303	0.000397	0.19718	0.48240	0.08475	1.54993	0.43821
8	313	0.0003843	0.20833	0.50968	0.05747	0.99454	-0.00547
9	Reference solution		0.23182				

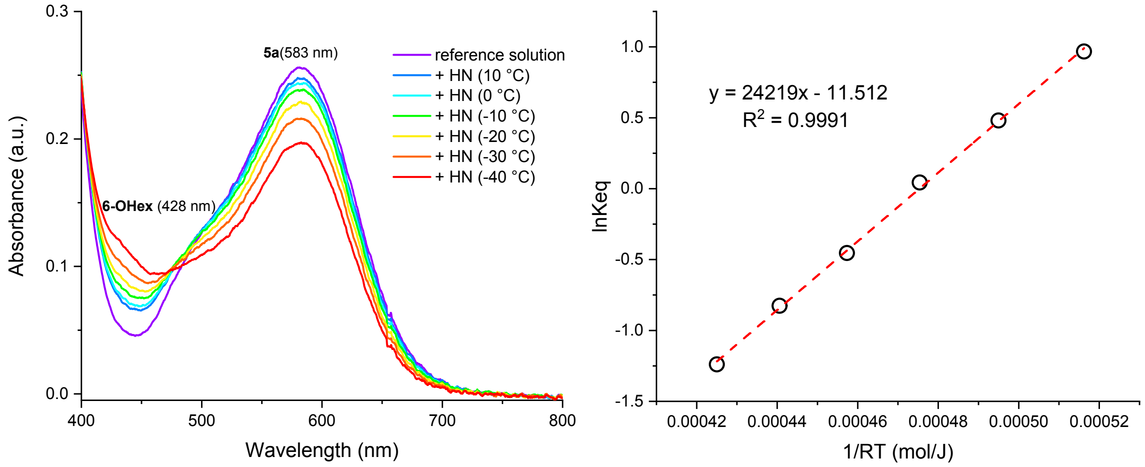

Figure S46. The UV-vis spectra and resulting Van't Hoff plots of the mixture of Mn-**5a** (0.567mM), hexanol (113.4 mM), and KO^{t}Bu (56.7 mM) in THF at different temperatures. According to the plots, equilibrium parameters were calculated out as: $\Delta H° =$ -24.2 ± 0.4 kJ·mol^{-1}; $\Delta S° =$ -95.7 ± 1.4 J·mol^{-1}·K^{-1}; $\Delta G° =$ 4.3 ± 0.6 kJ·mol^{-1} (298 K).

Table S7. Equilibrium constant output from UV-vis spectrum of the mixture of Mn-**5a** (0.567mM), hexanol (113.4 mM), and KO^{t}Bu (56.7 mM) in THF.

Entry	T (K)	1/RT (mol·J^{-1})	Absorbance (583nm, a.u.)	[**5a**](mM)	[**6-OHex**](mM)	K_{eq} (M^{-1})	ln (K_{eq})
1	283	0.000425	0.24714	0.54911	0.01804	0.28970	-1.23890
2	273	0.0004406	0.24319	0.54033	0.02682	0.43766	-0.82632
3	263	0.0004573	0.23811	0.52905	0.03810	0.63519	-0.45383
4	253	0.0004754	0.22824	0.50712	0.06003	1.04423	0.04328
5	243	0.000495	0.21570	0.47925	0.08790	1.61813	0.48127
6	233	0.0005162	0.19664	0.43691	0.13025	2.63114	0.96742
7	Reference solution		0.25526				

Table S8. Thermodynamic parameters for the addition of different alcohols to Mn amido complex **5a**.

Alcohol	ΔG°_{298K} (kJ·mol^{-1})	$\Delta H°$ (kJ·mol^{-1})	$\Delta S°$ (J·mol^{-1}·K^{-1})
MeOH	-13.5±0.8[a]	-49.8±0.6	-121.6±1.9
HexOH	-4.4±1.0[a]	-36.2±0.7	-106.9±2.5
BnOH	-11.3±0.6[a]	-40.5±0.4	-97.9±1.3

[a] Error from propagation of the error of $\Delta H°$ and $\Delta S°$, $\sigma_{\Delta G} = \sqrt{(\sigma_{\Delta H})^2 + (|T|\sigma_{\Delta S})^2}$. Errors in the enthalpy and entropy are from errors in the slope and intercept, respectively.

Table S9. Thermodynamic parameters associated with the coordination of hexanol to Mn amido complex **5a** in presence of base.

Equiv. of **5a**	Equiv. of HexOH	Equiv. of KOtBu	ΔG°_{298K} (kJ·mol^{-1})	ΔH° (kJ·mol^{-1})	ΔS° (J·mol^{-1}·K^{-1})
1	200	0	-4.4±1.0	-36.2±0.7	-106.9±2.5
1	200	50	-1.4 ± 0.4	-28.4 ± 0.3	-90.5 ± 0.9
1	200	100	4.3 ± 0.6	-24.2 ± 0.4	-95.7 ± 1.4

[a] Error from propagation of the error of ΔH° and ΔS°, $\sigma_{\Delta G} = \sqrt{(\sigma_{\Delta H})^2 + (|T|\sigma_{\Delta S})^2}$. Errors in the enthalpy and entropy are from errors in the slope and intercept, respectively.

Base effects on the equilibrium in Mn-8-benzyl alcohol mixtures:

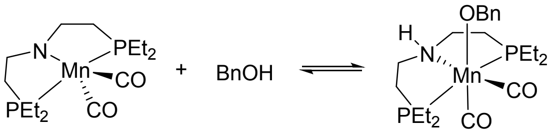

8 **9**

1.6 mL of 1.875 mM solution of **Mn-8** in THF were added to two identical cuvettes inside glovebox. The first sample was pure complex solution as reference. To the second sample was added corresponding amount of alcohol. For base effect study, base was mixed with the stock solution before adding to cuvette. The cuvettes were capped with Teflon seals, removed from glovebox, and loaded into UV-vis spectrometer and measured through transmission channel. The samples were heated or cooled to specified temperature and hold for 5 min before spectra were collected. All the transmission data were converted to absorbance for further analysis. The concentration of Mn species was determined by the absorbance at 491 nm.

0 eq. base experiment

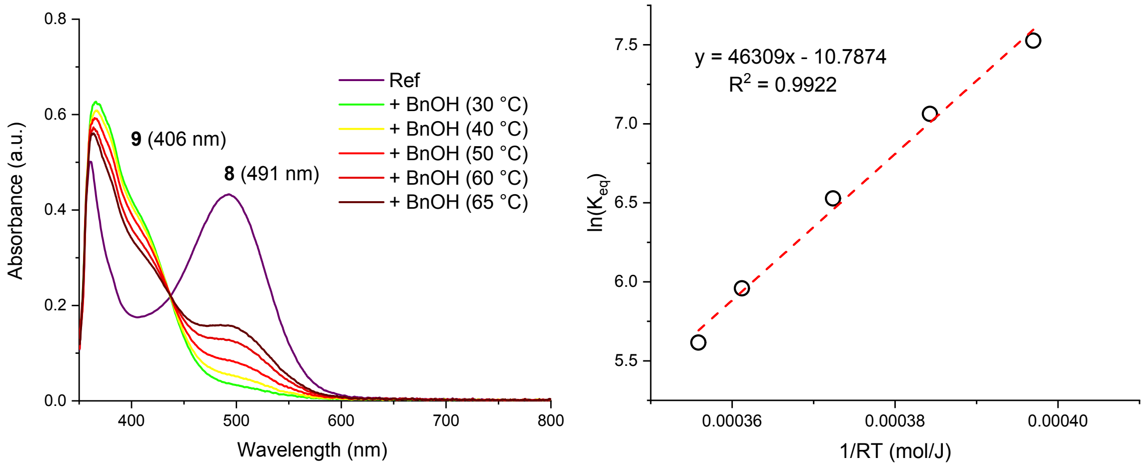

Figure S47. The UV-vis spectra and resulting Van't Hoff plots of the mixture of Mn-**8** (1.875 mM), and benzyl alcohol (7.5 mM) in THF at different temperatures. According to the plots, equilibrium parameters were calculated out as: $\Delta H^{\circ} =$ -46.3 ± 2.3 kJ·mol^{-1}; $\Delta S^{\circ} =$ -89.7 ± 7.3 J·mol^{-1}·K^{-1}; $\Delta G^{\circ} =$ -19.6 ± 3.1 kJ·mol^{-1} (298 K).

Table S10. Equilibrium constant output from UV-vis spectrum of the mixture of Mn-**8** (1.875 mM), and benzyl alcohol (7.5 mM) in THF.

Entry	T (K)	1/RT (mol·J^{-1})	Absorbance (583nm, a.u.)	[**8**](mM)	[**6-OHex**](mM)	K_{eq} (M^{-1})	ln (K_{eq})
1	303	0.000411	0.0368	0.1597	1.7153	1856.94	7.5267
2	313	0.000397	0.05505	0.2389	1.6361	1168.03	7.0631
3	323	0.000384	0.08476	0.3678	1.5072	683.80	6.5277
4	333	0.000372	0.12735	0.5526	1.3224	387.36	5.9594
5	338	0.000361	0.15808	0.6860	1.1890	274.66	5.6156
6	Reference solution		0.4321				

0.5 eq. base experiment

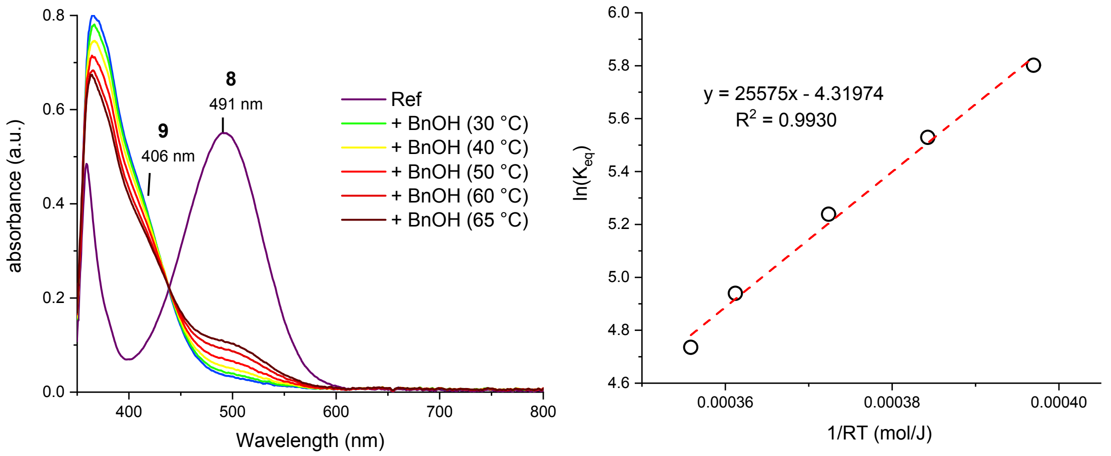

Figure S48. The UV-vis spectra and resulting Van't Hoff plots of the mixture of Mn-**8** (1.875 mM), KO^{t}Bu (18.7 mM), and benzyl alcohol (37.5 mM) in THF at different temperatures. According to the plots, equilibrium parameters were calculated out as: $\Delta H° =$ -25.6 ± 1.2 kJ·mol^{-1}; $\Delta S° =$ -35.9 ± 3.8 J·mol^{-1}·K^{-1}; $\Delta G° =$ -14.9 ± 1.6 kJ·mol^{-1} (298 K).

Table S11. Equilibrium constant output from UV-vis spectrum of the mixture of Mn-**8** (1.875 mM), KO^{t}Bu (18.7 mM), and benzyl alcohol (37.5 mM) in THF.

Entry	T (K)	1/RT (mol·J^{-1})	Absorbance (583nm, a.u.)	[**8**](mM)	[**6-OHex**](mM)	K_{eq} (M^{-1})	ln (K_{eq})
1	303	0.000396961	0.0428	0.1461	1.7289	330.90	5.8018
2	313	0.000384278	0.0548	0.1871	1.6879	251.91	5.5291
3	323	0.000372381	0.0708	0.2417	1.6333	188.43	5.2387
4	333	0.000361198	0.0912	0.3114	1.5636	139.74	4.9398
5	338	0.000355855	0.1077	0.3676	1.5074	113.94	4.7356
6	Reference solution		0.5497				

0.75 eq. base experiment

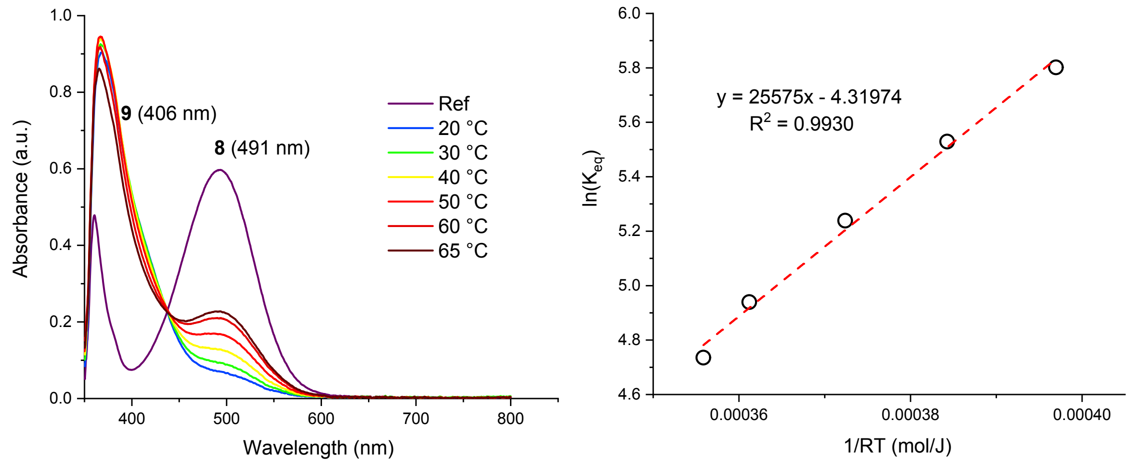

Figure S49. The UV-vis spectra and resulting Van't Hoff plots of the mixture of Mn-**8** (1.875 mM), KO^{t}Bu (28.1 mM), and benzyl alcohol (37.5 mM) in THF at different temperatures. According to the plots, equilibrium parameters were calculated out as: $\Delta H° =$ -28.6 ± 0.6 kJ·mol^{-1}; $\Delta S° =$ -53.2 ± 1.9 J·mol^{-1}·K^{-1}; $\Delta G° =$ -12.8 ± 0.8 kJ·mol^{-1} (298 K).

Table S12. Equilibrium constant output from UV-vis spectrum of the mixture of Mn-**8** (1.875 mM), KO^{t}Bu (28.1 mM), and benzyl alcohol (37.5 mM) in THF.

Entry	T (K)	1/RT (mol·J^{-1})	Absorbance (583nm, a.u.)	[**8**](mM)	[**6-OHex**](mM)	K_{eq} (M^{-1})	ln (K_{eq})
1	293	0.000410509	0.0712	0.2239	1.6511	205.66	5.3262
2	303	0.000396961	0.0932	0.2932	1.5818	150.18	5.0118
3	313	0.000384278	0.1281	0.4030	1.4720	101.39	4.6190
4	323	0.000372381	0.1681	0.5290	1.3460	70.38	4.2539
5	333	0.000361198	0.2095	0.6590	1.2160	50.85	3.9290
6	338	0.000355855	0.2268	0.7136	1.1614	44.78	3.8019
6	Reference solution		0.59613				

S9 – Computational Studies.

All quantum chemical calculations were performed using Gaussian16 software.[3] Geometries, relative stabilities and IR spectra were calculated at PBE0-D3/6-311+G(d,p) level of theory with a continuous description of the THF solvent using the SMD model.[4] The calculated IR frequencies were scaled with the factor f = 0.9576, which was chosen for best agreement of experimental and calculated data. It is applied to minimize the error of the harmonic approach for the vibration frequency calculation. The Half width of the plotted calculated spectra is 5 cm^{-1}. Time-dependent DFT calculations at the same level of theory (60 excitations) were carried out to simulate UV-vis spectra of selected complexes.

S9.1 Interconversion of polycarbonyl Mn(CNC) complexes

Additional computational analysis was performed to study the formation and interconversion in complexes **2**-**5**. Figure S50 summarizes the results of DFT calculations. The presented results point to the substantial flexibility of the coordination environment of the considered complexes. To eliminate uncertainty regarding the relative location of the counteranion, the tris-carbonyl Mn-CNC complexes **2**$^+$ and **3**$^+$ were represented as free cations. The very close thermodynamic stability of the two respective fac- and mer- isomers (**2**$^+$ → **3**$^+$, ΔG°298K = 3 kJ/mol) suggests a high flexibility of the ligand backbone. Their deprotonation yields the deprotonated tris-carbonyl amido configuration **4**, for which three distinct isomers featuring a *facial* coordination (**4**$_{\mathbf{fac}}$) as well as two types of *meridional* coordination (**4** and **4'**) of the CNC ligand were identified. The latter two complexes differ in the conformation of the -$CH_2CH_2NCH_2HC_2$- ligand backbone that affects also the coordination of the CO ligands. Despite relative close stabilities of the different configuration, the meridional symmetric configuration **4'** is ca. 12 kJ/mol stable than the other isomers. Furthermore, CO elimination is slightly endergonic and it yields complex bis-carbonyl deprotonated Mn-CNC complex **5**, represented in Figure S50 by the most stable configuration **5a**. The detailed description and summary of the conformational analysis of these species is given in the next sub-section.

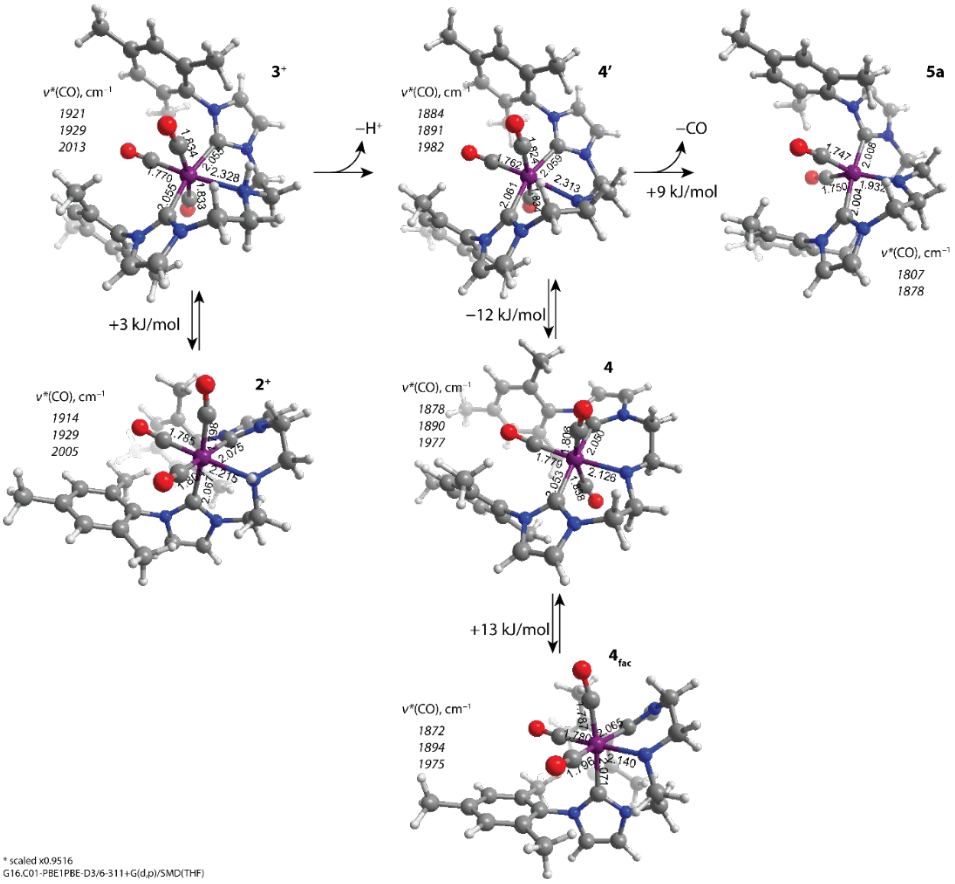

Figure S50. Optimized structures of Mn-CNC carbonyl complexes, calculated scaled frequencies of the respective carbonyl stretching vibrations, and the standard free energy changes for their interconversion.

S9.2 Conformational screening and assignment of the deprotonated $Mn(CO)_2CNC$ isomers

The analysis of the isomers of the deprotonated Mn-CNC complexes was carried out using the exhaustive conformational search for deprotonated $Mn(CO)_2CNC$ stoichiometry enabled by the in-house MACE workflow[5] followed by DFT calculations at the PBE1PBE-D3/6-311+G(d,p)/SMD(THF) level of theory. The MACE protocol automatically constructs all allowed configurations for the hypothetical 5-coordinated complexes without using any *a priori* information. The workflow starts with reading the ChemAxon[6] SMILES string of the octahedral complex and then generates all possible configurations of the system, considering both the stereochemistry of the octahedral center and the ligands. The next step is the filtration of the identical configurations, enantiomers, and impossible structures. For the selected stereomers 3D coordinates are generated using the distance geometry approach followed by an MM relaxation. For each configuration of the given complex 50 conformers are generated with the following pruning of similar conformers using a 0.5 RMSD threshold. MACE algorithm produced 4 distinct coordination geometries at the Mn center (2 *facial* and 2 *meridional*) and for each coordination environment 5 isomers with different conformations of the ligand backbone and, accordingly, relative conformation of the imidazole and amino-ligand moieties giving it total 20 distinct configurations.

The resulting configurations were further fully relaxed following by frequency calculations using Gaussian 16 revision C01 program package. Some of the starting configurations were converged upon the geometry optimization to identical isomers as confirmed by negligible RMSD and identical spectroscopic characteristics for the respective optimized structures. To confirm the exhaustive nature of the configurational search by MACE, additional configurations were manually constructed based on the optimized geometries, which all converged to the structures identified by the automated algorithm. The distinct configurations along with their relative stabilities (in terms of ZPE-corrected energies (ΔE_{ZPE}) and Gibbs free energies ($\Delta G^{o}_{298K,SMD(THF)}$) as well as the computed harmonic frequencies for the symmetric and asymmetric stretching vibration of the carbonyl moieties (scaling factor 0.9516 was uniformly applied) are summarized in Figures S51-52 for the *facial* and *meridional* $Mn(CO)_2CNC$ complexes, respectively. Both (distorted) trigonal bipyramidal and square pyramidal configurations of the Mn center were identified.

The results show a strong preference for the formation of the *mer*-configuration for $Mn(CO)_2CNC$ complexes with higher stabilities. Complex **2_B**, being the most stable conformer, with fully symmetric anti configuration of the ligand backbone is assigned to **5a** based on the agreements of configurations (see XRD details) and ν(CO). However, none of the conformers considered herein exhibited the red-shifted ν(CO) observed for **5b** in the experiment.

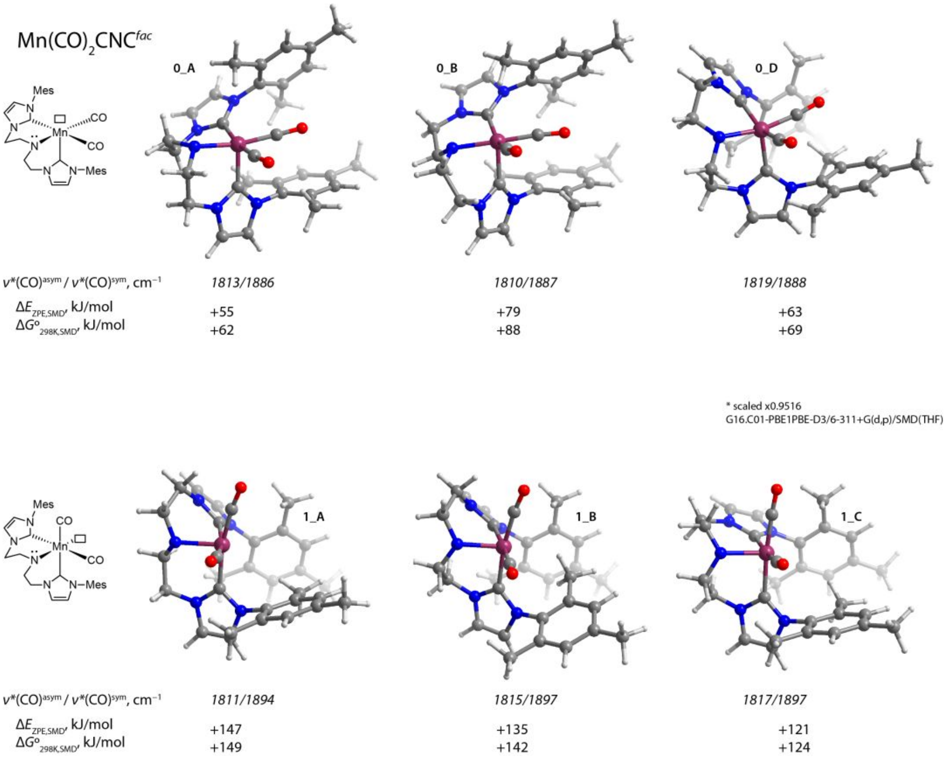

Figure S51. DFT-calculated structures, CO ligand vibration frequencies and relative stabilities for *fac* isomers of deprotonated $Mn(CO)_2CNC$.

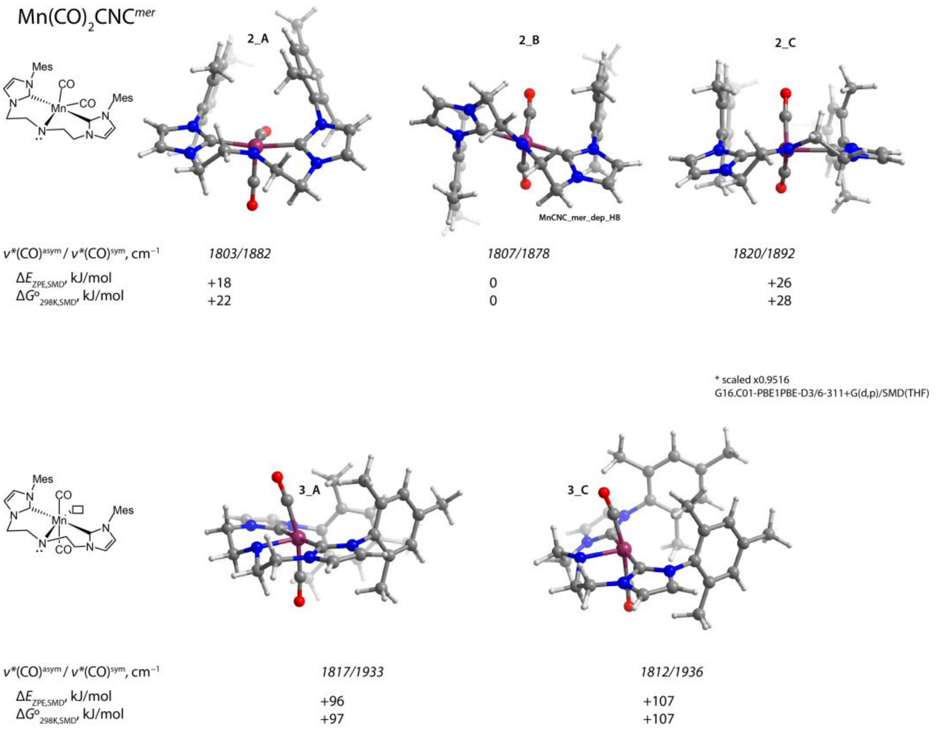

Figure S52. DFT-calculated structures, CO ligand vibration frequencies and relative stabilities for *mer* isomers of deprotonated $Mn(CO)_2CNC$. Conformer **2_B** is assigned to complex **5a** based on the agreements of ligand configurations (see XRD details in section S11) and *v*(CO) (Figure S55).

S9.3 The interaction of deprotonated $Mn(CO)_2CNC$ with alkoxide base and assignment of 5b

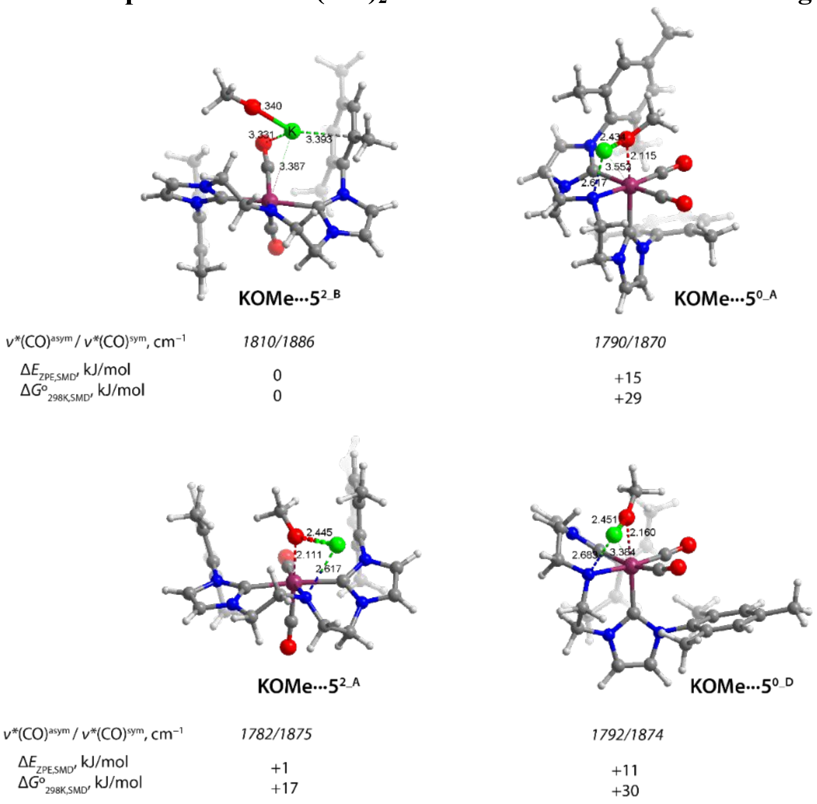

Figure 53. Relative stabilities and computed IR frequencies in the carbonyl region of KOMe adducts with $Mn(CO)_2CNC$.

We hypothesized that **5b** could be a metastable product of the KOR alkoxide base interaction with complex deprotonated $Mn(CO)_2CNC$. Therefore, the influence of the interaction with the alkoxide base on the vibrational characteristics of $Mn(CO)_2CNC$ were further analysed by considering a variety of coordination modes of a model KOMe alkoxide base with different (meta)stable conformers of $Mn(CO)_2CNC$. The direct coordination of K with the N of the deprotonated ligand as well as the stabilization of the resulting cationic complex by the methoxide anion in the second coordination sphere of Mn were considered. The most stable configurations, their relative stabilities and computed ν(CO) frequencies are summarized in Figure S53. Indeed, the deprotonation of reaction of $\mathbf{2}^+$ and $\mathbf{3}^+$ with excess alkoxide base can transiently produce either pure *fac*-configuration (Figure S51) or a distorted meridional (Figure 52) $Mn(CO)_2CNC$ featuring basic tetrahedral N center of the amido group, which could potentially coordinate K^+ species to the N center with the simultaneous coordination of the OR^- moiety to Mn. Such coordination is not allowed for the most stable fully symmetric trigonal bipyramidal complex **2_B** (also assigned to **5a**, see Figure S52, 55), confirmed by the nearly identical *ν*(CO) of its adduct complex **KOMe···5^{2}_B** to those of the parent complex **5a**. The other coordination modes considered resulted in a successful coordination of the KOMe moiety across the Mn-N bond resulting in slightly less thermodynamically stable adducts **KOMe···5^{0}_A**, **KOMe···5^{0}_D** and **KOMe···5^{2}_A**, among which the interconversion is expected to be kinetically hampered in the absence of proton donors or H_2 molecule, which could facilitate the pseudo-rotation of the N center of the backbone, giving rise

to the substantial metastability of these configurations. These KOMe adducts of deprotonated $Mn(CO)_2CNC$ are the only Mn carbonyl complexes considered here featuring the red-shifted ν(CO), suggesting they are **5b** observed in the experiment. This assignment is further supported by the higher apparent yield of these species upon the deprotonation of the facially-coordinated complex **2** than in the case of mer-complex **3**.

S9.4 Comparison of experimental and calculated spectra.

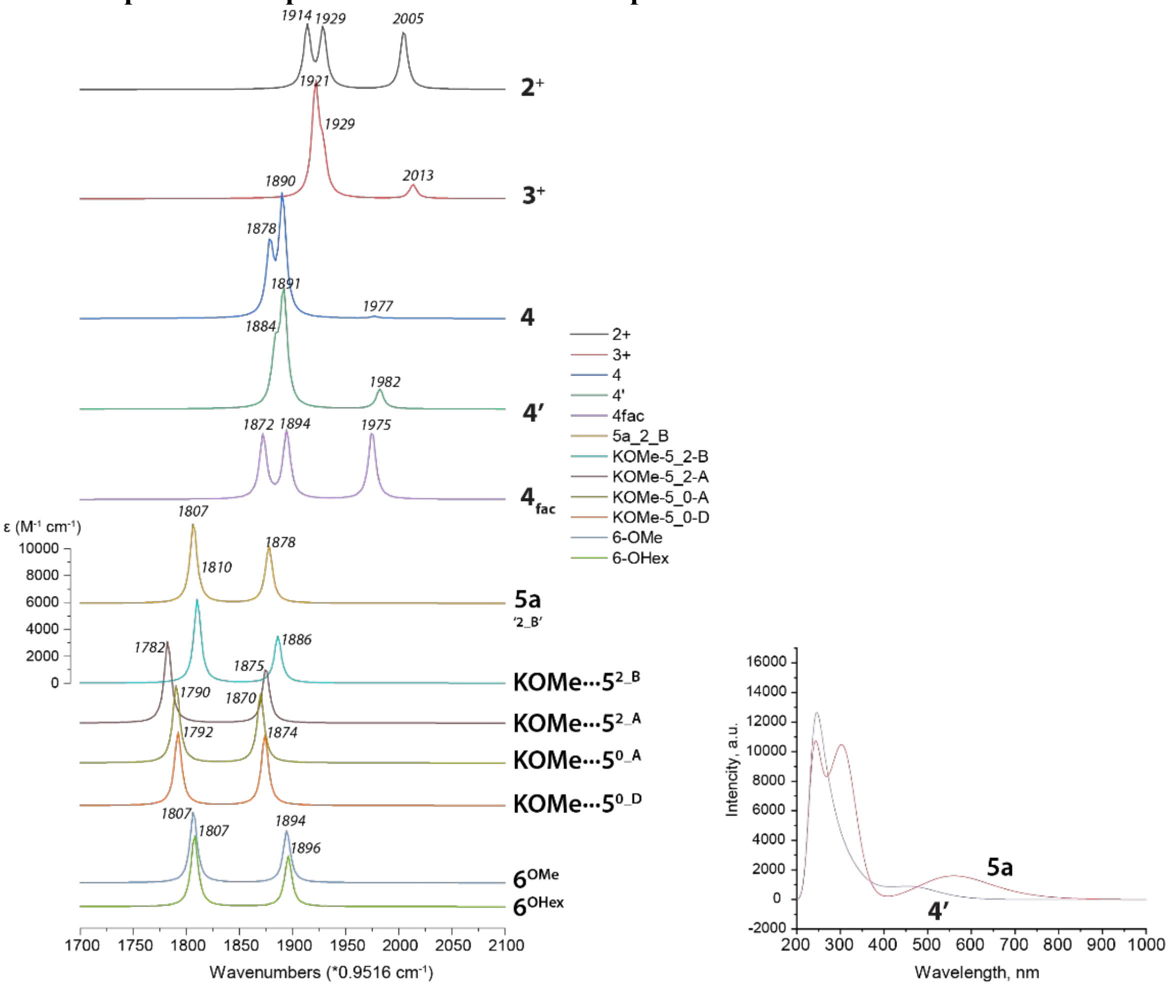

Figure S54 Summary of calculated FTIR spectra in CO stretching region for various MnCNC carbonyl complexes (left) and the DFT computed UV-vis spectra for complexes **4'** and **5a**.

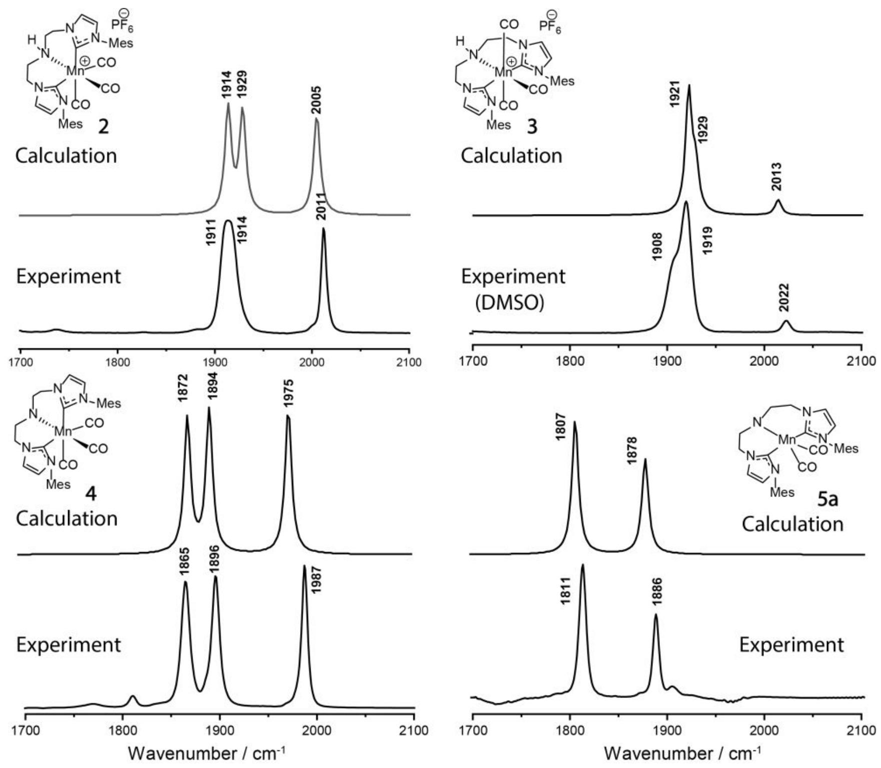

Figure S55. DFT-calculated and experimental IR spectra of **2** (top left), **3** (top right), **4** (bottom left) and **5a** (bottom right) . The results matched well, providing a reference for the set of scaling factor and also supporting the whole computational analysis.

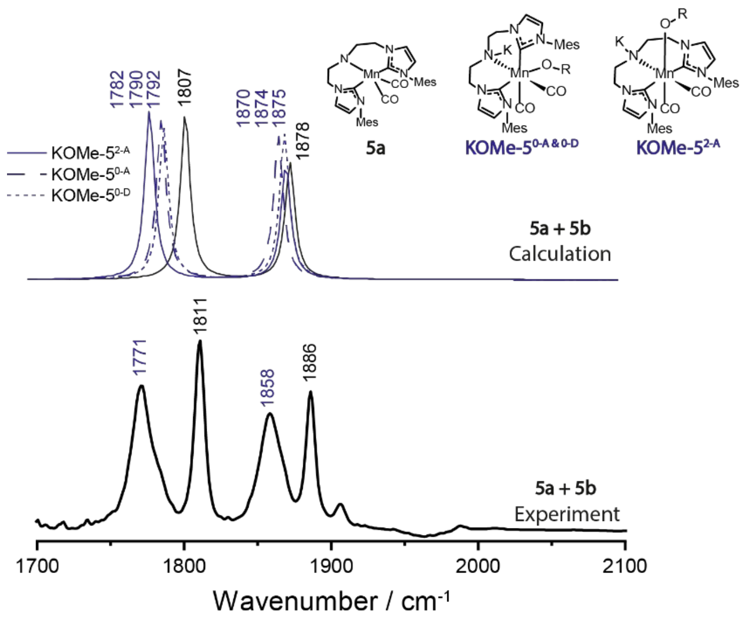

Figure S56. Experimental (bottom) and DFT-calculated IR spectra (top) of the mixture of **5a** (black) and **5b** (blue) in THF. The experimental broad band of 1858, 1771 cm^{-1} should be the mixture of adducts **KOR-5** with the backbone in different geometries.

S9.5 Analysis of base effects on chemical potentials of components of inhibitory equilibrium

COSMO-RS calculations of condition-dependent reaction free energies

Condition-dependent free energies of the formation of Mn-alkoxide were calculated using COSMO-RS model[15].

Methodology and terms: This is a hybrid statistical solvation model which is used to calculate the chemical potentials of molecular liquids of any composition. It describes intermolecular interactions as interaction of molecular surfaces based on COSMO polarization charge densities. A key part of the method is an empirical formula for the interaction energy between two surface segments, which considers electrostatic interactions, hydrogen bonding, and dispersion. Combination of this formula and the statistical thermodynamics gives a possibility to calculate condition-dependent chemical potentials in a few seconds and thus to easily model condition-dependent free energies of liquid-phase chemical processes.

The algorithm for calculating the condition-dependent free energy based on DFT computations consists of five steps:

1. Compute free energy of the considered molecule at DFT level of theory with any implicit solvation model (PCM, COSMO, SMD, CPCM), ΔG_{solv}^{QM}.
2. Compute free energy of the considered molecule at DFT level of theory in a gas phase, ΔG_{gas}^{QM}.
3. Optimize the geometry of all particles forming the solution (frequency computations are not required).
4. For the optimized geometries calculate COSMO-files (one SCF cycle with solvent described using the COSMO model). Obtained COSMO-files contain all information about surface segments that are required for COSMO-RS calculations.
5. Compute COSMO-RS free energy of solvation, $\Delta G_{solv}^{COSMO-RS}$, for the required solution composition which is given by COSMO-files and the corresponding mole fractions.
6. Compute resulting condition-dependent free energy of the considered molecule as: $\Delta G = \Delta G_{solv}^{QM} - \Delta G_{gas}^{QM} + \Delta G_{solv}^{COSMO-RS}$.

Calculations:

Gas-phase single-point energies which are necessary to get the SMD solvation energies were calculated with Gaussian16 at the PBE1PBE-D3/6-311+G(d,p) level of theory. COSMO files were generated for the optimized geometries with the Turbomole suite[7] using standard BP_TZVP parametrization. Free energies of solvation were computed in the COSMOtherm package[8] using BP_TZVP parametrization in the molar frame. It should be noted that when calculating $\Delta G_{solv}^{COSMO-RS}$, the COSMO-RS solvation energy of reagents and products was obtained for solutions corresponding to 0% and 100% conversion,

into amide or alkoxide complexes respectively. Three experimental conditions were considered, including pure THF (#1) and two KO^{t}Bu/THF solutions (#2 and 3) (Table S13).

Table S13. Composition of model solutions employed to analyze condition-dependencies of Mn-alkoxide formations represented by molar fractions of all compounds in THF as the primary solvent; identical to those used in UV-Vis study.

Solution	Conversion	$Mn(CO)_2CNC$	ROH	$MnOR(CO)_2CN^HC$	K^+	$^tBuO^-$	THF
#1	0%	0.000046	0.004500	0.000000	0.000000	0.000000	0.995454
	100%	0.000000	0.004454	0.000046	0.000000	0.000000	0.995500
#2	0%	0.000045	0.008919	0.000000	0.002238	0.002238	0.986560
	100%	0.000000	0.008874	0.000045	0.002238	0.002238	0.986605
#3	0%	0.000045	0.008879	0.000000	0.004456	0.004456	0.982164
	100%	0.000000	0.008874	0.000045	0.004456	0.004456	0.986605

Additional calculations on alcohol binding to **5a** were performed to confirm the effect of base promotion on the free energies of the reaction between complex **5a** (most stable isomer **2_B** as identified earlier, see Figure S52) and several alcohols, namely methanol, hexanol, and benzyl alcohol. For each manganese alkoxide (**$MnOR(CO)_2CN^HC$**), several conformers were generated (optimized structures for most stable conformations are shown in Figure S57; Cartesian coordinates for all optimized structures are provided as the supporting information) and their relative stability was analyzed with SMD and COSMO-RS free energies (Table S14). The relative stabilities of low-energy conformers are similar for both solvation models, and some differences appear from the 15 kJ/mol cut-off. Thermodynamic parameters of the reaction were calculated for the most stable conformers of the initial and final manganese complexes (Table S15). The substantial mismatch between the experimental and calculated thermodynamic parameters for the methoxide is attributed by a systematic error in the theoretical solvation energies for short-chain alcohols. For example, earlier studies reported two-fold higher errors in predicted energy of solvation of ethanol compared to butanol with the SMD and COSMO-RS models.[9] This is due to the imperfect description of hydrogen bonding interactions, of which the importance increases with decreasing alcohol size and increasing solute hydrophilicity[10], which is the case for the polar manganese alkoxide.

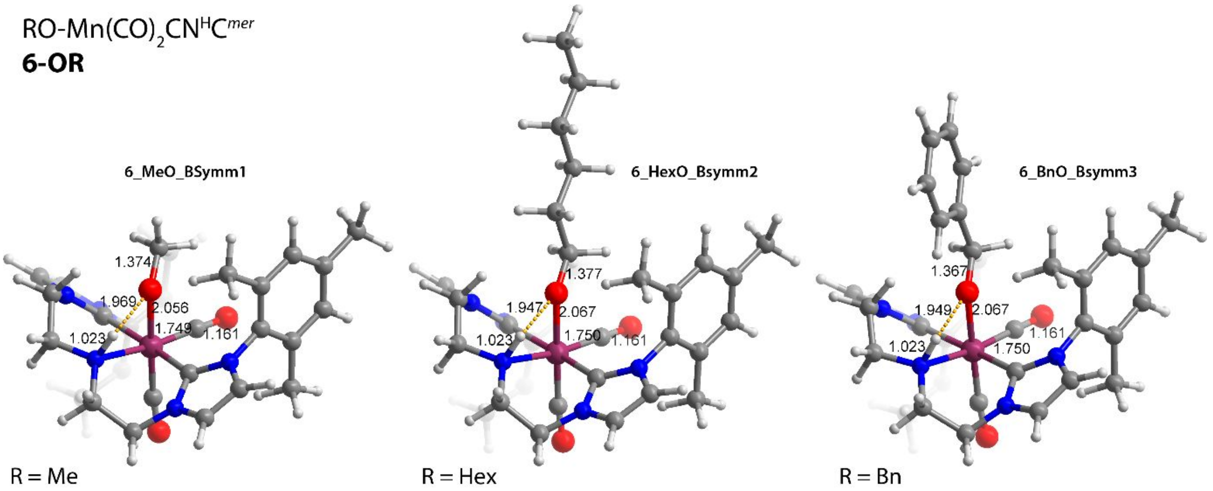

Figure S57.The most stable conformers of $Mn(CO)_2CNC$ alkoxide adducts ($MnOR(CO)_2CNHC$) with (a) methanol, (b) hexanol, and (c) benzyl alcohol. Other structures can be found in supplementary information in XYZ format.

Table S14. Results of conformational analysis - the relative free energy (kJ·mol^{-1}) of Mn alkoxide conformers.[a]

Alcohol	Conformer	$\Delta G^{SMD\ a)}$	$\Delta G^{s1\ b)}$	$\Delta G^{s2\ b)}$	$\Delta G^{s3\ b)}$
MeOH	Bsymm1	0.0	0.0	0.0	0.0
	A5	10.8	17.4	17.8	17.9
	A1	14.2	22.7	23.0	23.0
	A3	33.6	42.0	43.5	43.9
HexOH	Bsymm2	0.0	0.0	0.0	0.0
	Bsymm1	3.5	4.2	4.2	4.2
	A2	9.9	15.1	15.3	15.3
	A5	11.0	15.2	15.2	15.2
	Bsymm3	15.4	14.3	14.3	14.3
	A1	17.6	24.5	24.6	24.6
	A4	20.1	21.0	21.5	21.6
	A3	21.1	22.2	22.2	22.2
BnOH	Bsymm3	0.0	0.0	0.0	0.0
	Bsymm2	0.0	0.0	0.0	0.0
	A3	30.1	27.1	27.2	27.2
	A4	30.8	38.2	38.2	38.2

a) ΔG^{SMD} is a free energy for the ideal THF solution, obtained with the SMD solvation model. b) ΔG^{s1}, ΔG^{s2}, and ΔG^{s3} are free energies for the real solutions #1-3 (**Table S13**), obtained with the COSMO-RS solvation model. All values are relative to that of the most stable conformer which is taken as zero.

Analysis:

Having identified the appropriate conformers (Figure S57, Table S14) the cosmo-RS analysis was performed for reaction "**r1** + **r2** = **p**" where **r1** and **r2** are reagents and **p** is an Mn alkoxide product. We find that alcohol reagent (**r2**) is largely affected by varying base concentrations. Both enthalpy and entropy for **r2** change significantly, while these changes for Mn complexes (**r1** and **p**) are negligible. The data for the whole reaction and individual reagents and products shown below in Table S15:

Table S15. Impact of COSMO-RS solvation to theoretical thermodynamic parameters of Mn alkoxide formation.[a]

Alcohol	MeOH			HexOH			BnOH		
**Solution**	**#1**	**#2**	**#3**	**#1**	**#2**	**#3**	**#1**	**#2**	**#3**
Overall reaction: **r1** + **r2** = **p**									
ΔG	-0.2	4.2	6.9	4.0	8.3	10.7	-12.0	-7.5	-4.6
ΔH	-32.9	-15.5	-3.8	-29.1	-12.0	-1.1	-45.4	-28.5	-15.3
ΔS	-113	-68	-37	-114.5	-70.1	-41.0	-115.4	-72.6	-36.9
Solvation contributions for overall reaction and individual components: Mn amide (**r1**) , alcohol (**r2**) and Mn alkoxide (**p**)									
ΔG_{solv}	9.2	13.6	16.2	16.0	20.3	22.7	21.2	25.7	28.6
G_{solv}(r1)	-102.7	-102.5	-102.3	-102.6	-102.4	-102.2	-102.6	-102.4	-102.2
**G_{solv}(r2)**	**-19.3**	**-23.8**	**-26.4**	**-33.0**	**-37.2**	**-39.6**	**-41.2**	**-45.6**	**-48.5**
G_{solv}(p)	-112.8	-112.6	-112.5	-119.6	-119.3	-119.1	-122.6	-122.3	-122.2
ΔH_{solv}	35.3	52.7	64.3	45.0	62.0	72.9	52.2	69.0	82.3
H_{solv}(r1)	-143.7	-143.3	-143.2	-143.6	-143.2	-143.1	-143.6	-143.2	-143.0
**H_{solv}(r2)**	**-48.7**	**-66.0**	**-77.6**	**-68.6**	**-85.6**	**-96.4**	**-78.7**	**-95.5**	**-108.6**
H_{solv}(p)	-157.0	-156.6	-156.5	-167.3	-166.8	-166.6	-170.1	-169.6	-169.4
ΔS_{solv}	87.7	131.2	161.3	97.0	140.1	168.4	104.0	145.6	180.2
S_{solv}(r1)	-137.6	-136.9	-137.0	-137.7	-137.1	-137.2	-137.6	-136.9	-137.0
**S_{solv}(r2)**	**-98.4**	**-141.8**	**-171.9**	**-119.5**	**-162.5**	**-190.6**	**-125.9**	**-167.3**	**-201.8**
S_{solv}(p)	-148.3	-147.5	-147.5	-160.1	-159.4	-159.4	-159.5	-158.6	-158.6

Free energies and enthalpies are given in $kJ\cdot mol^{-1}$, and entropies in $J\cdot mol^{-1}\cdot K^{-1}$. (r1), (r2), and (p) indexes correspond to $Mn(CO)_2CNC$, alcohol, and manganese alkoxide complex.

ΔG, ΔH, and ΔS are the total thermodynamic parameters of the reaction obtained by a combination of DFT and COSMO-RS, and other quantities with the "solv" subscript are the thermodynamic parameters of solvation obtained with the COSMO-RS model. Bold text indicates the solvation parameters which significantly depend on the solution composition.

S10 – Crystal Structure Analysis Details.

X-ray crystal structure determination of Mn complex 2:

Crystals suitable for X-ray diffraction were obtained by slow methanol vapour diffusion into mixture acetonitrile and acetone solution of **2**. Crystallographic data: $C_{31}H_{35}F_6MnN_5O_3P$, C_2H_3N, *Fw* =766.60 g mol^{-1}, light yellow block, 0.33x0.26x0.09 mm^3 (crystal size), monoclinic, *P2$_1$*, a = 8.4244(1) Å, b = 12.9639(2) Å, c = 16.0201(2) Å, α = 90 °, β = 99.0762(5) °, γ = 90 °, V = 1727.70(4) Å^3, Z = 2, Dx = 1.474 g cm^{-3}, μ = 0.504 mm^{-1}. 44314 reflections were measured by a Bruker D8-Venture Photon area detector (MoKα radiation, λ = 0.71073 Å^{-1}) up to a resolution of (sin(Θ)/λ)max = 0.61 Å^{-1} at a temperature of 100 K.

Data analysis:

Reflections were corrected for adsorption and scaled on the basis of multiple measured reflections using the SADABS program (0.723 – 0.770 correction range).[11] 6435 Reflections were unique (*R*int = 0.030). Using ShelXle[12], the structures were solved with SHELXS-14[13] by using direct methods and refined with SHELXL-2018[13] on *F*2 for all reflections. Non-hydrogen atoms were refined by using anisotropic displacement parameters. Positions of hydrogen atoms were calculated for idealized positions. 462 Parameters were refined without restraints. $R1$ = 0.022 for 6435 reflections with $I > 2\sigma(I)$ and $wR2$ = 0.056 for 6473 reflections. S = 1.070. Residual electron density was between 0.25 and -0.21 eÅ-3. Geometry calculations and checks for higher symmetry were performed with the PLATON program.[14]

X-ray crystal structure determination of Mn complex 3:

Crystals suitable for X-ray diffraction were obtained by slow diethyl ether vapour diffusion into DMSO solution of **3**. Crystallographic data: $C_{31}H_{34}MnN_5O_3$, P F_6, *Fw* =724.54 g mol^{-1}, yellow chunk, 0.27x0.23x0.12 mm^3 (crystal size), monoclinic, *P2$_1$/c*, a = 8.1392(1) Å, b = 11.1681(2) Å, c = 35.7486(6) Å, α = 90 °, β = 96.1631(5) °, γ = 90 °, V = 3230.75(9) Å^3, Z = 4, Dx = 1.490 g cm^{-3}, μ = 0.534 mm^{-1}. 54520 reflections were measured by a Bruker D8-Venture Photon area detector (MoKα radiation, λ = 0.71073 Å^{-1}) up to a resolution of (sin(Θ)/λ)max = 0.61 Å^{-1} at a temperature of 100 K.

Data analysis:

Reflections were corrected for adsorption and scaled on the basis of multiple measured reflections using the SADABS program (0.848 – 0.914 correction range).[10] 6045 Reflections were unique (*R*int = 0.029). Using ShelXle[11], the structures were solved with SHELXS-14[12] by using direct methods and refined with SHELXL-2018[12] on *F*2 for all reflections. Non-hydrogen atoms were refined by using anisotropic displacement parameters. Positions of hydrogen atoms were calculated for idealized positions. No

hydrogen could be determined at N1 in the X-ray refinement. 430 Parameters were refined without restraints. $R1 = 0.059$ for 6045 reflections with $I > 2\sigma(I)$ and $wR2 = 0.123$ for 6094 reflections. $S = 1.297$. Residual electron density was between 0.88 and -0.65 eÅ-3. Geometry calculations and checks for higher symmetry were performed with the PLATON program.[14]

X-ray crystal structure determination of Mn complex 5a:

Crystals suitable for X-ray diffraction were obtained by slow pentane vapour diffusion into tetrahydrofuran solution of **5a**. Crystallographic data: $C_{30}H_{34}MnN_5O_2$, Fw =551.56 g mol^{-1}, blue platelet, 0.37x0.13x0.11 mm^3 (crystal size), monoclinic, $P2_1/c$, a = 11.4131(2) Å, b = 16.5258(3) Å, c = 15.3650(3) Å, α = 90 °, β = 103.7359(7) °, γ = 90 °, V = 2815.12(9) Å^3, Z = 4, Dx = 1.301 g cm^{-3}, μ = 0.504 mm^{-1}. 49556 reflections were measured by a Bruker D8-Venture Photon area detector (MoKα radiation, λ = 0.71073 Å^{-1}) up to a resolution of (sin(Θ)/λ)max = 0.61 Å^{-1} at a temperature of 100 K.

Data analysis:

Reflections were corrected for adsorption and scaled on the basis of multiple measured reflections using the SADABS program (0.883 – 0.928 correction range).[10] 5033 Reflections were unique (Rint = 0.029). Using ShelXle[11], the structures were solved with SHELXS-14[12] by using direct methods and refined with SHELXL-2018[12] on $F2$ for all reflections. Non-hydrogen atoms were refined by using anisotropic displacement parameters. Positions of hydrogen atoms were calculated for idealized positions. 349 Parameters were refined without restraints. $R1 = 0.029$ for 5033 reflections with $I > 2\sigma(I)$ and $wR2$ = 0.073 for 5354 reflections. $S = 1.085$. Residual electron density was between 0.31 and -0.31 eÅ-3. Geometry calculations and checks for higher symmetry were performed with the PLATON program.[14]

CCDC-2099208: complex **2**, CCDC-2099206: complex **3**, CCDC-2099207: complex **5a** contains the supplementary crystallographic data for this paper. These data can be obtained free of charge from The Cambridge Crystallographic Data Centre *via http://www.ccdc.cam.ac.uk/data_request/cif*

S11 – References.

1. Filonenko, G. A.; Aguila, M. J. B.; Schulpen, E. N.; van Putten, R.; Wiecko, J.; Muller, C.; Lefort, L.; Hensen, E. J. M.; Pidko, E. A., Bis-N-heterocyclic Carbene Aminopincer Ligands Enable High Activity in Ru-Catalyzed Ester Hydrogenation. *J. Am. Chem. Soc.* **2015,** *137*, 7620-7623.

2. S. Elangovan, M. Garbe, H. Jiao, A. Spannenberg, K. Junge, M. Beller, *Angew. Chem. Int. Ed.* **2016**, *49*, 15364-15368.

3. Frisch, M. J.; Trucks, G. W.; Schlegel, H. B.; Scuseria, G. E.; Robb, M. A.; Cheeseman, J. R.; Scalmani, G.; Barone, V.; Petersson, G. A.; Nakatsuji, H.; Li, X.; Caricato, M.; Marenich, A. V; Bloino, J.; Janesko, B. G.; Gomperts, R.; Mennucci, B.; Hratchian, H. P.; Ortiz, J. V; Izmaylov, A. F.; Sonnenberg, J. L.; Williams-Young, D.; Ding, F.; Lipparini, F.; Egidi, F.; Goings, J.; Peng, B.; Petrone, A.; Henderson, T.; Ranasinghe, D.; Zakrzewski, V. G.; Gao, J.; Rega, N.; Zheng, G.; Liang, W.; Hada, M.; Ehara, M.; Toyota, K.; Fukuda, R.; Hasegawa, J.; Ishida, M.; Nakajima, T.; Honda, Y.; Kitao, O.; Nakai, H.; Vreven, T.; Throssell, K.; Montgomery Jr., J. A.; Peralta, J. E.; Ogliaro, F.; Bearpark, M. J.; Heyd, J. J.; Brothers, E. N.; Kudin, K. N.; Staroverov, V. N.; Keith, T. A.; Kobayashi, R.; Normand, J.; Raghavachari, K.; Rendell, A. P.; Burant, J. C.; Iyengar, S. S.; Tomasi, J.; Cossi, M.; Millam, J. M.; Klene, M.; Adamo, C.; Cammi, R.; Ochterski, J. W.; Martin, R. L.; Morokuma, K.; Farkas, O.; Foresman, J. B.; Fox, D. J. Gaussian16 Revision C.01. 2016.

4. Marenich, A. V.; Cramer, C. J.; Truhlar, D. G., Universal solvation model based on solute electron density and on a continuum model of the solvent defined by the bulk dielectric constant and atomic surface tensions. *The Journal of Physical Chemistry B* **2009,** *113*, 6378-6396.

5. Chernyshov, I.V.; Pidko, E.A. in preparation.

6. http://www.chemaxon.com/

7. TURBOMOLE V7.3 2018, a development of University of Karlsruhe and Forschungszentrum Karlsruhe GmbH, 1989-2007, TURBOMOLE GmbH, since 2007; available from http://www.turbomole.com/

8. COSMOtherm, Version C3.0, Release 17.01; COSMOlogic GmbH & Co. KG, http://www.cosmologic.de/

9. Marenich, A. V.; Cramer, C. J.; Truhlar, D. G., Universal solvation model based on solute electron density and on a continuum model of the solvent defined by the bulk dielectric constant and atomic surface tensions. *The Journal of Physical Chemistry B* **2009,** *113*, 6378-6396.

10. Freire, M. G.; Ventura, S. P.; Santos, L. M.; Marrucho, I. M.; Coutinho, J. A., Evaluation of COSMO-RS for the prediction of LLE and VLE of water and ionic liquids binary systems. *Fluid Phase Equilib.* **2008,** *268*, 74-84.

11. Bruker **(2013)**. *APEX2*, *SAINT, XPREP* and *SADABS*. Bruker AXS Inc., Madison, Wisconsin USA.

12. B. Hübschle, G. M. Sheldrick, D Dittrich, ShelXle: a Qt graphical user interface for SHELXL. *J. Appl. Cryst.* **2011**, *44*, 1281 – 1284.

13. G. M. Sheldrick, SHELXT - Integrated space-group and crystal-structure determination. *Acta Cryst.* **2015**, *C71*, 3-8.

14. *PLATON*. A. L. Spek, Structure validation in chemical crystallography. *Acta Cryst.* **2009**, *D65*, 148–155.

15. (a) Klamt, A., Eckert, F., Arlt, W. COSMO-RS: An Alternative to Simulation for Calculating Thermodynamic Properties of Liquid Mixtures, Ann. Rev. Chem. Biomol. Eng. **2010**, *1*, 101-122; (b) Klamt, A. The COSMO and COSMO-RS solvation models, *WIREs Comput Mol Sci,* **2017**, *8: e1338*
